# Enhancing stress resilience in rice (*Oryza sativa* L.) through profiling early-stage morpho-physiological and molecular responses to multiple abiotic stress tolerance

**DOI:** 10.3389/fpls.2024.1342441

**Published:** 2024-02-08

**Authors:** Kathiresan Pravin Kumar, Ramamoorthy Pushpam, Swaminathan Manonmani, Muthurajan Raveendran, Subramanian Santhiya, Alagarsamy Senthil

**Affiliations:** ^1^ Centre for Plant Breeding and Genetics, Tamil Nadu Agricultural University (TNAU), Coimbatore, India; ^2^ Directorate of Research, Tamil Nadu Agricultural University (TNAU), Coimbatore, India; ^3^ Department of Crop Physiology, Directorate of Crop Management, Tamil Nadu Agricultural University (TNAU), Coimbatore, India

**Keywords:** rice, early-stage, multiple abiotic stress, morpho-physiological characters, molecular profiling, marker-trait association

## Abstract

Under changing climatic conditions, crop plants are more adversely affected by a combination of various abiotic stresses than by a single abiotic stress. Therefore, it is essential to identify potential donors to multiple abiotic stresses for developing climate-resilient crop varieties. Hence, the present study was undertaken with 41 germplasm accessions comprising native landraces of Tamil Nadu, Prerelease lines and cultivars were screened independently for drought, salinity, and submergence at the seedling stage during Kharif and Rabi 2022–2023. Stress was imposed separately for these three abiotic stresses on 21-day-old seedlings and was maintained for 10 days. The studied genotypes showed a significant reduction in plant biomass (PB), Relative Growth Index (RGI), relative water content (RWC), leaf photosynthesis, chlorophyll fluorescence, and Chlorophyll Concentration Index (CCI) under drought followed by salinity and submergence. Stress-tolerant indices for drought, salinity, and submergence revealed significant variation for plant biomass. Furthermore, a set of 30 SSR markers linked to drought, salinity, and submergence QTLs has been used to characterize 41 rice germplasm accessions. Our analysis suggests a significantly high polymorphism, with 28 polymorphic markers having a 93.40% in 76 loci. The mean values of polymorphic information content (PIC), heterozygosity index (HI), marker index (MI), and resolving power (RP) were 0.369, 0.433, 1.140, and 2.877, respectively. Jaccard clustering grouped all the genotypes into two major and six subclusters. According to STRUCTURE analysis, all genotypes were grouped into two major clusters, which are concurrent with a very broad genetic base (*K* = 2). Statistically significant marker-trait associations for biomass were observed for five polymorphic markers, *viz.*, RM211, RM212 (drought), RM10694 (salinity), RM219, and RM21 (submergence). Similarly, significant markers for relative shoot length were observed for RM551 (drought), RM10694 (salinity), and ART5 (submergence). Notably, the genotypes Mattaikar, Varigarudan samba, Arupatham samba, and APD19002 were identified as potential donors for multiple abiotic stress tolerance. Thus, identifying the genetic potential of germplasm could be useful for enhancing stress resilience in rice.

## Introduction

Changing climate and growing global population are significant concerns for rice (*Oryza sativa* L) production (Manasa et al., 2023). Climate change disturbs the consistency and intensity of hydrological occurrences, threatening crop yield and food security. Primary regions experiencing these effects include South and Southeast Asia, Sub-Saharan Africa, and Latin America, covering both uplands and shallow rainfed lowlands (Radha et al., 2023). In order to address the challenge of feeding an anticipated global population of nine billion by 2050, we must substantially enhance rice production. To put this into perspective, the current yield stands at 104 million tons, and our target is to generate an additional 160 million tons of rice (www.FAO.org). Amid the changing climate, almost half of the rice cultivation regions are impacted, leading to a 42% decrease in yield worldwide due to various abiotic stresses (Muthu et al., 2020).

Rice is life for millions of farmers and is considered a staple food for over a billion people in the world. Rice cultivation primarily thrives in tropical and subtropical climates, which are mainly affected by drought, salinity, and submergence. On a global scale, 90% of rice production and consumption takes place in Asia (Adhikari et al., 2019). When subjected to drought, crops face a significant reduction in yield, particularly when the stress coincides with the reproductive stage (Garrity and O'Toole, 1994; Oladosu et al., 2020; Verma et al., 2014). Despite Asia being the major producer, approximately 34 Mha of rainfed lowland and 8 Mha of upland rice cultivable area are annually impacted by drought resulting in a yield reduction of 13% to 35% (Panda et al., 2022). Enhancing drought tolerance poses a challenge due to the unpredictable nature of drought stress and the complex response mechanisms of plants (Takahashi et al., 2020). Likewise, salinity is a widespread problem in both coastal and marginal inland environments, limiting rice production in 30% of rice growing area, encompassing 45 million hectares of irrigated land and 32 million hectares of dry land worldwide (Singh et al., 2016; FAO, 2018; Ravikiran et al., 2018). The elevated salt levels disrupt water and nutrient absorption by the roots, causing an imbalance in the plant’s metabolism, ultimately resulting in decreased plant growth, leading to a yield loss of up to 35% (Farooq et al., 2015). Although rice is a semi-aquatic plant, it is well-suited for stagnant conditions due to its well-established aerenchyma, enabling oxygen transportation through the roots. However, recurrent flooding in lowland and deep-water rice regions, impacts an area of 12–14 million hectares in India, leading to a yield loss of up to 32% (Oladosu et al., 2020). The ability of tolerance to endure submergence relies on its carbohydrate levels (Alpuerto et al., 2016).

Utilizing natural genetic resources is the primary avenue for advancing stress resilience in rice cultivation (Marone et al., 2021). Studying natural variation not only helps us comprehend the genetic mechanisms underpinning tolerance during crucial growth stages but also advances our understanding of the associated physiological process (Ismail and Horie, 2017). Traditional rice landraces possess a robust genetic foundation that offers enhanced adaptability and protection against various biotic and abiotic stresses (Binodh et al., 2023). Therefore, landraces play a vital role as integral plant genetic resources, holding immense potential as donors for developing multiple stress tolerance. Significant advancements have been made in the past few decades leading to the identification of recognized donors, such as N22 for drought (Vikram et al., 2016), Pokkali for salinity (Singh and Sengar, 2014), and FR13A for submergence (Xu et al., 2006). Currently, due to changing climatic conditions, crops in stress-affected areas are expected to face a combination of abiotic stresses rather than single stresses (Slama et al., 2015; Wani et al., 2016). Thus, breeding for tolerance to a single abiotic stress may be risky, as plant responses to combined stresses differ from individual stress responses. When rice seedlings encounter various abiotic stresses during their initial growth phase, issues may arise due to suboptimal crop establishment, decreased length of roots and shoots, reduced leaf area, and early seedling death (Krishnamurthy et al., 2016). Hence, seedling stage tolerance to multiple abiotic stresses is crucial for better crop establishment, robust vegetative growth, and ultimately higher yield. To tackle this, a trait-based breeding approach in crop improvement programs is more effective than focusing on yield-based breeding (Paleari et al., 2017). Numerous studies have indicated the use of PEG-6000 for drought tolerance (Binodh et al., 2023), sodium chloride (NaCl) for salinity adaptation (Kakar et al., 2019), and the maintenance of a water level ranging from 90 cm to 120 cm for submergence tolerance (Samanta et al., 2022) were employed for quick screening during the seedling stage to identify promising candidates with stress tolerance.

Recent advancements in crop genetics have led to the creation of molecular tools that assist in faster and more efficient breeding techniques. Examining genetic diversity and population structure is valuable for identifying superior breeding materials and enhancing breeding efficiency. The use of DNA-based markers resulted in the identification of markers linked to QTLs or genic regions of particular traits, making the breeding process quicker and more precise to select preferred plants more rapidly (Singh et al., 2015). Several studies have highlighted specific quantitative trait loci (QTLs) linked to stress tolerance. During the reproductive stage of drought stress, several yield QTLs such as *qDTY 1.1*, *qDTY 2.1*, *qDTY 3.1*, and *qDTY 12.1* were found to be closely associated with particular markers, including RM104 (Ghimire et al., 2012), RM2634 (Muthu et al., 2020), RM168 (Dixit et al., 2014), and RM28166 (Mishra et al., 2013). Moreover, RM3412 is found to be associated with the *saltol* region of seedling stage salinity (Krishnamurthy et al., 2016), and ART5 is found to be associated with the *sub1* region for submergence (Sarkar and Bhattacharjee, 2011). In the present era, rapid changes in climatic conditions emphasize the need for developing broad-spectrum genetic resistance in rice. This genetic resistance, with its inbuilt tolerance to these stresses, offers an economically viable and sustainable option to improve rice productivity under multiple stress conditions. Despite considerable efforts through traditional breeding approaches, molecular introspection of genetic diversity could reveal more precise information about the genetic variation, which would be helpful for genetic improvement breeding by identifying multiple abiotic stress tolerance. Therefore, this study aimed to evaluate the physiological responses and genetic diversity residing among the landraces, prerelease cultures, and cultivars to multiple abiotic stresses in order to identify potential donors to be employed in climate-resilient breeding programs.

## Materials and methods

In total, 41 diverse rice genotypes, including native landraces of Tamil Nadu, prerelease lines, and cultivars were evaluated independently for three different abiotic stress tolerance involving respective tolerant checks, *viz*., IR64Drt1 for drought possessing *qDTY 2.2* and *qDTY 4.1* QTL (Binodh et al., 2023), FL478 for salinity having *saltol* QTL (Muthu et al., 2020), and FR13A for submergence having *Sub1* QTL (Xu et al., 2006), whereas IR64 for drought and salinity and IR42 for submergence were used as susceptible checks (**Supplementary Table 1**). The present investigation was carried out in the Department of Rice, Centre for Plant Breeding and Genetics, Tamil Nadu Agricultural University, Coimbatore, Tamil Nadu, during Kharif and Rabi 2022–2023.

### Experimental design for screening

**Figure 1:**
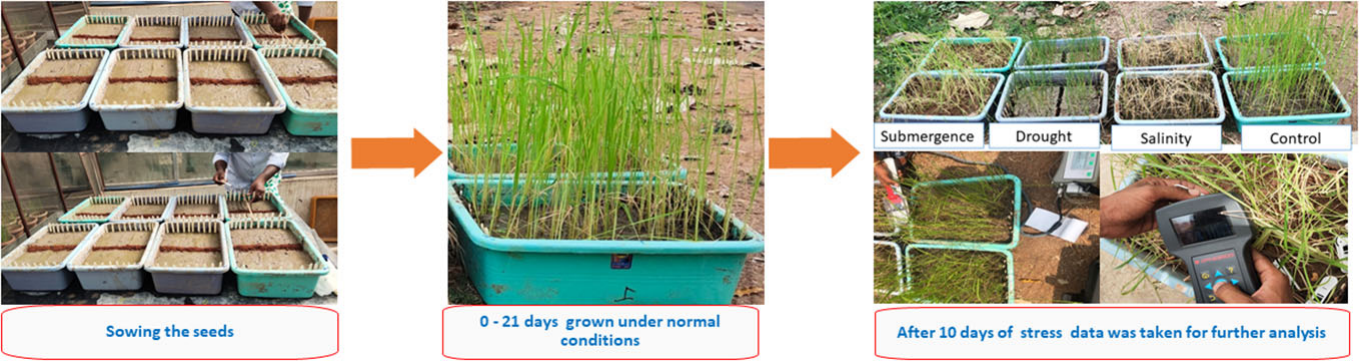
Experimental procedure for screening seedling stage abiotic stress tolerance.

The experiment was carried out in a complete randomized design (CRD) with three independent replications. Uniform-sized seeds were surface sterilized with a 0.1% HgCl_2_ solution for 5 min and thoroughly washed with distilled water for 10 min. The seeds were then placed in a Petri dish (50 seeds in each petri dish) and soaked in distilled water for 24 h under room temperature (25°C ± 2°C). Pregerminated seeds from each genotype were sown in plastic trays (30 cm × 20 cm × 10 cm) filled with field soil and farmyard manure (3:1 proportion). Twelve seedlings were maintained per genotype at a spacing of 1.5 cm × 1.5 cm. In each tray, the recommended dose of fertilizers, *viz*., urea, single super phosphate (SSP), and murate of potash (MOP) was applied 10 days after sowing. The trays were regularly watered. The average solar radiation throughout the growth period measured was 1,011 ± 40 µmol m^−2^ s^−1^ using a digital pyranometer (PMA2145 Class 1, Solar Light Co. Inc., Philadelphia, USA) with a maximum temperature of 33 ± 4 was measured using a digital thermometer (HTC-1, Aptechdeals, Jipvi tools, China), and relative humidity ranging from 67% to 75% was measured using a digital hygrometer (HTC-1, Aptechdeals, Jipvi tools, China). The seedlings were grown under standard conditions and nurtured for a duration of 21 days. Subsequently, stress was imposed separately, adhering to the specified protocol for each stress (Behera et al., 2023). One set of genotypes was maintained under nonstress conditions (control) until the end of the experiment (**Figure 1**).

### Phenotype screening for drought tolerance

Drought stress was induced by withholding irrigation in plastic trays containing a set of 41 genotypes for a period of 10 days. Plant growth and physiological parameters were recorded upon the completion of the stress period.

### Phenotype screening for salinity tolerance

Salinity stress was applied by irrigating the genotypes with saline water with 12 EC dsm^−1^ for a period of 10 days. Plant growth and physiological parameters were recorded upon the completion of the stress period.

### Phenotype screening for submergence tolerance

For 10 days, 21-day old seedlings were subjected to submergence screening by completely submerging the seedlings in 90 cm ± 5 cm of water. Following submergence, the water was drained, and plants were allowed to recover. Plant growth and physiological parameters were recorded 7 days after de-submergence.

### Measurement of plant growth parameter

The measurements of plant growth parameters involved the assessment of shoot length and plant biomass. For biomass determination, whole seedlings were carefully uprooted and oven-dried at 80°C for 48 hours using a hot air oven (LTHOS-4, Labtech, Delhi, India).

The relative shoot length (RSL) was calculated as per Nahar et al. (2018).


RSL = Shoot length under stress / shoot length under control

The Relative Growth Index (RGI), was calculated by Kumar et al. (2014)


RGI = plant biomass under stress/plant biomass under control

Stress-Tolerant Index for drought, salinity, and bubmergence were calculated following Behera et al. (2023).


$$\text{STI =}(\frac{Plant\;biomass\;under\;stress}{Plant\;biomass\;under\;control})/(\frac{Average\;plant\;biomass\;under\;stress}{Average\;plant\;biomass\;under\;control})$$

The Stress Tolerance Index (STI) was used to identify genotypes capable of achieving high yields under both drought stress and non-stress irrigated conditions. A high STI value indicates a greater degree of stress tolerance (Muthuramu and Ragavan, 2020). Based on the values and desirability criteria, various genotypes were categorized as highly tolerant (STI > 1.00), tolerant (STI: 0.76–1.00), moderately tolerant (STI: 0.51–0.75), and susceptible (STI< 0.50).

### Measurement of physiological parameters

Several physiological parameters were recorded in control and stress conditions. Relative water content (RWC) was estimated following the protocol of Barik et al. (2018). The second leaf of each plant was used to measure photosynthetic rate (PN), transpiration rate (*e*), and stomatal conductivity (gs) using an open-system photosynthesis gas analyzer (LCi-SD, ADC, UK). Similarly, chlorophyll fluorescence (Fo, Fm, and Fv/Fm) and chlorophyll content (Chlorophyll Content Index) were also measured on the same leaves using chlorophyll fluorometer OS30p (ADC Bioscientific, Hoddesdon, United Kingdom) and CCM (ADC Bioscientific, Hoddesdon, United Kingdom).

### Microsatellite marker-based molecular analysis

Microsatellite markers (SSR markers) linked with target traits, *viz*., 14 for drought, eight for salinity, and eight for submergence utilized in this study (**Table 1**). Details of the linked markers were obtained from earlier reports by Tabkhkar et al. (2018) and Radha et al. (2023), and their respective chromosomal positions and sequence information were sourced from the Gramene marker database (https://archive.gramene.org/markers/).

**Table 1:** List of SSR markers (associated QTLs, trait, chromosome, marker distance/marker region) used for genotyping in the present study.

Marker	AT (°C)	Chr	QTL	Trait	MD/R (cM)	Forward sequence	Reverse sequence	Reference
RM431	55	1	*qDTY 1.1*	Grain yield	12.13	TCCTGCGAACTGAAGAGTTG	AGAGCAAAACCCTGGTTCAC	Vikram et al. (2011)
RM212	55	1	*qDTY 1.1*	Grain yield	19.1	CCACTTTCAGCTACTACCAG	CACCCATTTGTCTCTCATTATG	Sandhu et al. (2018)
RM3825	55	1	*qDTY 1.1*	Grain yield	10.19	AAAGCCCCCAAAAGCAGTAC	GTGAAACTCTGGGGTGTTCG	Prince et al. (2015)
RM11943	55	1	*qDTY 2.1*	Grain yield	17.96	CTTGTTCGAGGACGAAGATAGGG	CTTGTTCGAGGACGAAGATAGGG	Vikram et al. (2011)
RM3412	55	1	*Saltol*	Na^+^/K^+^ ratio	11.5	AAAGCAGGTTTTCCTCCTCC	CCCATGTGCAATGTGTCTTC	Muthu et al. (2020)
RM8094	55	1	*Saltol*	Na^+^/K^+^ ratio	60.6	AAGTTTGTACACATCGTATACA	CGCGACCAGTACTACTACTA	Thomson et al. (2010)
RM10694	55	1	*Saltol*	Na^+^/K^+^ ratio	11	TTTCCCTGGTTTCAAGCTTACG	AGTACGGTACCTTGATGGTAGAAAGG	Thomson et al. (2010)
RM140	55	1	*Saltol*	Na^+^/K^+^ ratio	10.7	TGCCTCTTCCCTGGCTCCCCTG	GGCATGCCGAATGAAATGCATG	Thomson et al. (2010)
RM7075	55	1	*Saltol*	Na^+^/K^+^ ratio	74.2	TATGGACTGGAGCAAACCTC	GGCACAGCACCAATGTCTC	Thomson et al. (2010)
RM1287	55	1	*Saltol*	Na^+^/K^+^ ratio	58.1	GTGAAGAAAGCATGGTAAATG	CTCAGCTTGCTTGTGGTTAG	Thomson et al. (2010)
RM104	55	1	*Sub1*	Na^+^/K^+^ ratio	8.3	GGAAGAGGAGAGAAAGATGTGTGTCG	TCAACAGACACACCGCCACCGC	Ghimire et al. (2012)
RM211	55	2	*qDTY 2.2*	Grain yield	14.4	CCGATCTCATCAACCAACTG	CTTCACGAGGATCTCAAAGG	Palanog et al. (2014)
RM250	55	2	*qDTY 2.3*	Grain yield	170.1	GGTTCAAACCAAGCTGATCA	GATGAAGGCCTTCCACGCAG	Palanog et al. (2014)
RM2634	55	2	*qDTY 2.3*	Grain yield	80.95	GATTGAAAATTAGAGTTTGCAC	TGCCGAGATTTAGTCAACTA	Muthu et al. (2020)
RM22	55	3	*qDTY 3.2*	Grain yield	7.7	GGTTTGGGAGCCCATAATCT	CTGGGCTTCTTTCACTCGTC	Vikram et al. (2011)
RM168	55	3	*qDTY 3.1*	Grain yield	37.3	TGCTGCTTGCCTGCTTCCTTT	GAAACGAATCAATCCACGGC	Donde et al. (2020)
RM232	55	3	*qDTY 1.1*	Grain yield	76.7	CCGGTATCCTTCGATATTGC	CCGACTTTTCCTCCTGACG	Palanog et al. (2014)
RM520	55	3	*qDTY 3.1*	Grain yield	138.7	AGGAGCAAGAAAAGTTCCCC	GCCAATGTGTGACGCAATAG	Angaji et al. (2010)
RM7097	55	3	*Saltol*	Na^+^/K^+^ ratio	115.6	GGGAGGAGGAGAGGAGATTG	TTAGGCCTGCACTTTTGGAG	Angaji et al. (2010)
RM551	55	4	*qDTY 4.1*	Grain yield	143.6	AGCCCAGACTAGCATGATTG	GAAGGCGAGAAGGATCACAG	Malik et al. (2022)
RM204	55	6	*qDTY 6.1*	Grain yield	25.1	GTGACTGACTTGGTCATAGGG	GCTAGCCATGCTCTCGTACC	Donde et al. (2020)
RM589	55	6	*qDTY 6.1*	Grain yield	2.7	ATCATGGTCGGTGGCTTAAC	CAGGTTCCAACCAGACACTG	Venuprasad et al. (2012)
RM11	55	7	*Saltol*	Na^+^/K^+^ ratio	93.8	TCTCCTCTTCCCCCGATC	ATAGCGGGCGAGGCTTAG	Singh et al. (2021)
RM337	55	8	*Sub1*	Shoot elongation	15.2	GTAGGAAAGGAAGGGCAGAG	CGATAGATAGCTAGATGTGGCC	Xu et al. (2006)
ART5	58	9	*Sub1*	Shoot elongation	6.39	CAGGGAAAGAGATGGTGGA	TTGGCCCTAGGTTGTTTCAG	Muthu et al. (2020)
SUB1AB1	60	9	*Sub1*	Shoot elongation	6.4	CATGTTCCATAGCCATCGACT	GAGCGAAGAGAGCTACCTGAA	Septiningsih et al. (2009)
SUB1BC3	60	9	*Sub1*	Shoot elongation	16.72	CATGGGTAAAATTGCCATCC	GCTTGAGGGTGAGTGGAGAG	Septiningsih et al. (2009)
RM219	55	9	*Sub1*	Shoot elongation	5.5	CGTCGGATGATGTAAAGCCT	CATATCGGCATTCGCCTG	Biswas et al. (2013)
RM316	55	9	*Sub1*	Shoot elongation	1.5	CTAGTTGGGCATACGATGGC	ACGCTTATATGTTACGTCAAC	Xu et al. (2006)
RM21	55	11	*Sub1*	Shoot elongation	73.1	ACAGTATTCCGTAGGCACGG	GCTCCATGAGGGTGGTAGAG	Angaji et al. (2010)

### Genomic DNA isolation and PCR reaction

Genomic DNA was isolated from 14-day-old seedlings using a modified CTAB method (Aboul-Maaty and Oraby, 2019). The purified DNA was dissolved in 50 µl of Tris-EDTA buffer (1×) and stored at −20°C for subsequent handling. The PCR reaction was set up using a Thermal Cycler (Bio-Rad T100). A 10-µl mixture was prepared, comprising 3.5 µl of PCR master mix (2× PCR master Mix-Red, smART Prime), 1 µl of SSR marker, 1 µl of genomic DNA (40 ng/µl), and 4.5 µl of distilled water, following the protocol outlined by Singh et al. (2010).

### Analysis of PCR product and genetic similarity analysis

The amplified PCR products were analyzed in the Bio-Rad gel documentation system (USA). Amplified bands were scored manually for the presence (indicated as “1”) or absence (indicated as “0”) of each SSR marker. The marker traits such as polymorphism information content (PIC), marker index (MI), resolving power (RP), and heterozygosity index (HI) were calculated following Al-Daej et al. (2023). Pairwise genetic similarity between genotypes was calculated using Jaccard’s coefficient employing the R Programme (Jaccard, 1908; Prabakaran et al., 2010).

### Population structure analysis

To analyze the population structure among rice genotypes, we employed the model-based program STRUCTURE (version 2.3.4) (Zhang et al., 2022). The admixture model was used to determine the ancestry of the population. We adjusted the parameter Lambda, which represents the distribution of allelic frequencies, to 1 for result interpretation. To ensure consistency, we performed five independent runs with a long burn-in and Markov Chain Monte Carlo (MCMC) set at 50,000 iterations each. The number of possible populations (*K*) was tested, ranging from 1 to 10. The population number was determined by selecting the *K* value that yielded the highest posterior probability, Pr(*X* = *K*), referred to as LnP(*D*) in the STRUCTURE output. The optimal value of *K* was identified using *ad-hoc* statistics Δ*K*, as proposed by Evanno et al. (2005), and analyzed using ‘*Structure Harvester’* https://taylor0.biology.ucla.edu/structureHarvester/.

### Identification of marker-trait association

The estimation of marker-trait association was done by single marker analysis with the regression method using single factor standard analysis of variance (Ramchander et al., 2022). The marker-trait association with *p*-value< 0.05 was identified as significant, and the proportion of phenotypic variance of the trait accounted by the marker was estimated in percent *R* ^2^.

### Statistical analysis

An analysis of variance (ANOVA) was carried out to assess the variations among plant growth and physiological parameters. Furthermore, for the statistical significance of the parameter’s means, we employed the Fisher’s least significant difference (LSD) test. All statistical analysis was carried out using the package “*doebioresearch*” in the “R” platform.

## Results

### Impact of plant growth parameters

**Figure 2:**
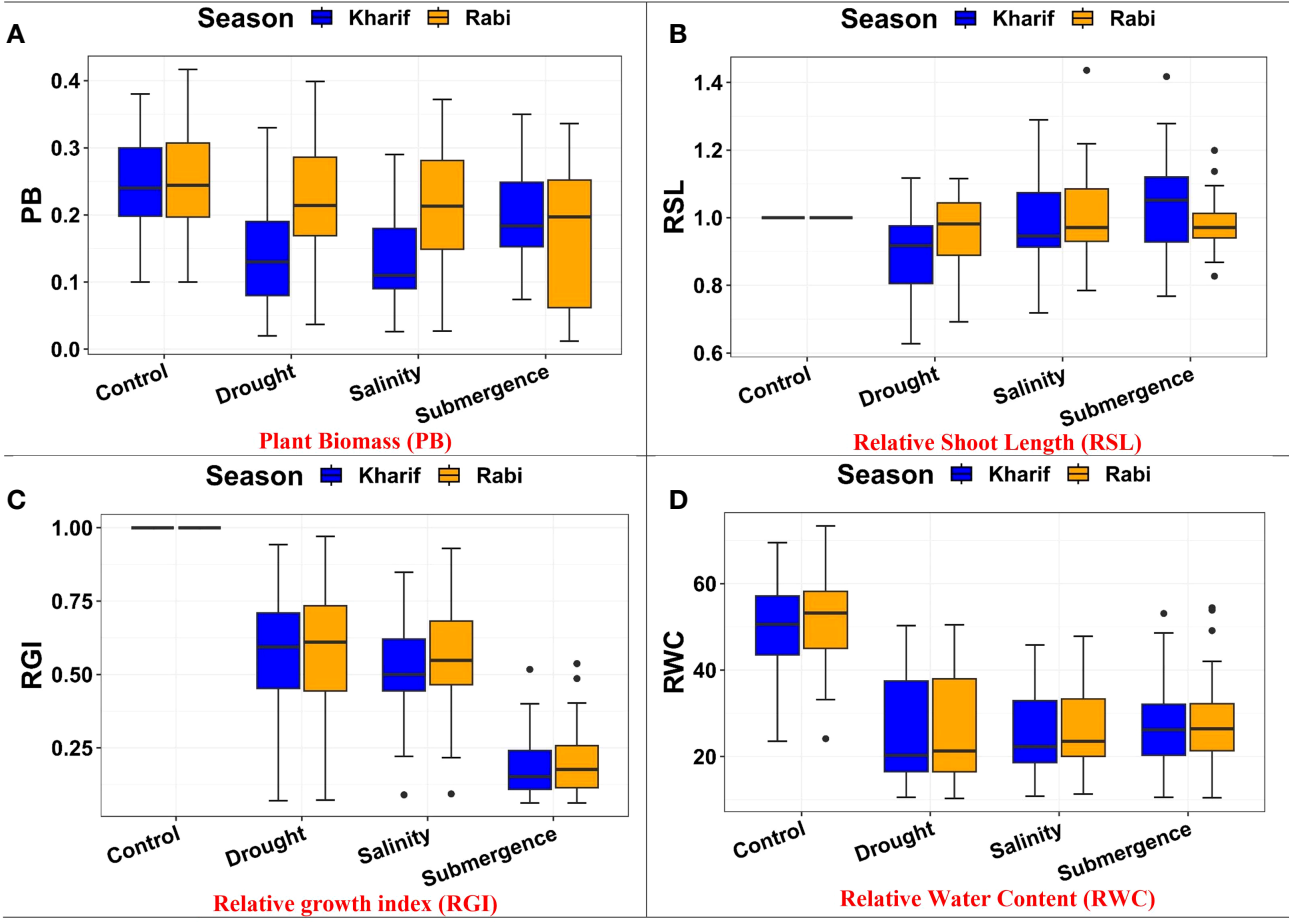
Performance of morphology traits: **(A)** plant biomass, **(B)** relative shoot length, **(C)** relative growth index, and **(D)** relative water content under varied stresses during both seasons.

The ANOVA of growth patterns from both seasons and pooled analysis revealed significant differences among the genotypes (*p<* 0.01) for almost all the traits under study across all stress conditions (**Table 2**). The phenotypic response of individual genotypes under different stress conditions during Kharif and Rabi are shown in **Figure 2**. The impact of multiple abiotic stresses, *viz*., drought, salinity, and submergence, on plant growth parameters, *viz*., relative shoot length, plant biomass, and relative growth index, exhibited substantial reductions within the studied genotypes, as shown in **Supplementary Table 2**. When compared to the control, relative shoot length notably increased by 0.5% under submergence under pooled conditions, whereas in the case of drought and salinity, it was decreased by about 7.400% and 0.300%, respectively. In the case of submergence, FR13A was observed to have a lower RSL under both seasons, and the genotype Mattaikar was found to be on par with FR13A. In all the stress conditions, there was a significant reduction in plant biomass of about 13.253% under drought, 16.466% under salinity, and 26.104% under submergence when compared to the control. Similarly, the relative growth index showed a reduction of about 36.900%, 42.00%, and 75.600% under drought, salinity, and submergence, respectively, under both seasons over pooled conditions. Under each stress condition, plant biomass of the genotype exhibited significant differences under both seasons. Notably, under drought, the genotypes APD19002, Arupatham samba, Mattaikar, Norungan, and Varigarudan samba exhibited higher plant biomass when compared to the tolerant check (IR64Drt1). Likewise, under salinity, the genotypes, *viz*., Kappikar, Arupatham samba, Mattaikar, and Norungan, revealed higher biomass compared to the tolerant check (FL478). In the case of submergence, the plant biomass of Mattaikar was found to be on par with the tolerant check (FR13A).

**Table 2:** Analysis of variance (ANOVA) for morpho-physiological parameters of various stresses during Kharif and Rabi during 2022–2023.

Mean sum of square												
Stress	Control			Drought			Salinity			Submergence		
Season	Kharif	Rabi	Pooled	Kharif	Rabi	Pooled	Kharif	Rabi	Pooled	Kharif	Rabi	Pooled
**Treatment**	41	41	41	41	41	41	41	41	41	41	41	41
**DF**	40	40	40	40	40	40	40	40	40	40	40	40
**PB**	0.015^***^	0.016^***^	0.0103^***^	0.014^***^	0.020^***^	0.010^***^	0.014^***^	0.026^***^	0.011^***^	0.016^**^	0.034^***^	0.0102
**RSL**	0	0	0	0.049^***^	0.032^***^	0.020^**^	0.058^***^	0.047^***^	0.029^***^	0.06^***^	0.015^***^	0.015
**RGI**	0	0	0	0.441^***^	0.19^***^	0.16^***^	0.11^***^	0.094^***^	0.059^***^	0.27^***^	0.045^***^	0.0672
**RWC**	311.26^***^	334.52^***^	213.31^***^	442.01^***^	470.42^***^	303.60^***^	313.98^***^	306.093^***^	205.05^***^	288.57^***^	311.55^***^	199.19^***^
**TR**	1.18^***^	1.39^***^	0.85^***^	0.69^***^	0.81^***^	0.50^***^	2.42^***^	2.68^***^	1.69^***^	0.66^***^	0.68^***^	0.45^***^
**SC**	0.00086^***^	0.00091^***^	0.00041^***^	0.00072^***^	0.00073^***^	0.00048^***^	0.00063^***^	0.00068^***^	0.00058^***^	0.00080^***^	0.00082^***^	0.00053^***^
**PR**	1.57^***^	1.74^***^	1.10^***^	2.31^***^	2.744^***^	1.67^***^	2.23^***^	2.43^***^	1.55^***^	1.44^***^	1.56^***^	1.003^***^
**CCI**	0.10^***^	0.12^***^	0.076^***^	0.633^***^	0.06^***^	0.44^***^	0.20^***^	0.20^***^	0.13^***^	0.32^***^	0.39^***^	0.23^***^
**FO**	777.58^***^	891.55^***^	552.58^***^	821.10^***^	899.58^***^	568.75^***^	946.80^***^	973.27^***^	635.86^***^	274.33^***^	328.33^***^	198.35^***^
**FM**	3739.00^***^	3997.30^***^	2544.64^***^	6093.80^***^	6735.4^***^	4247^***^	5385.90^***^	5499.50^***^	3612.7^***^	4423.00^***^	4866.60^***^	3067.1^***^
**FV/FM**	0.036^***^	0.04^***^	0.024^***^	0.021^***^	0.020^***^	0.013^***^	0.021^***^	0.02^***^	0.013^***^	0.013^**^	0.043^***^	0.014^***^
**FV/FO**	1.37^***^	1.46^***^	0.94^***^	0.767^***^	0.825^***^	0.526^***^	0.663^***^	0.74^***^	0.46^***^	1.51^***^	1.71^***^	1.06^***^

^**^0.05 and ^***^0.01—levels of significance. PB, plant biomass; RSL, relative shoot length; RGI, relative growth index; RWC, relative water content; TR, transpiration rate; SC, stomatal conductance; PR, photosynthetic rate; CCI, chlorophyll content index. Chlorophyll fluorescence: FO, FM, FV/FM, and FV/FO.

**Figure 3:**
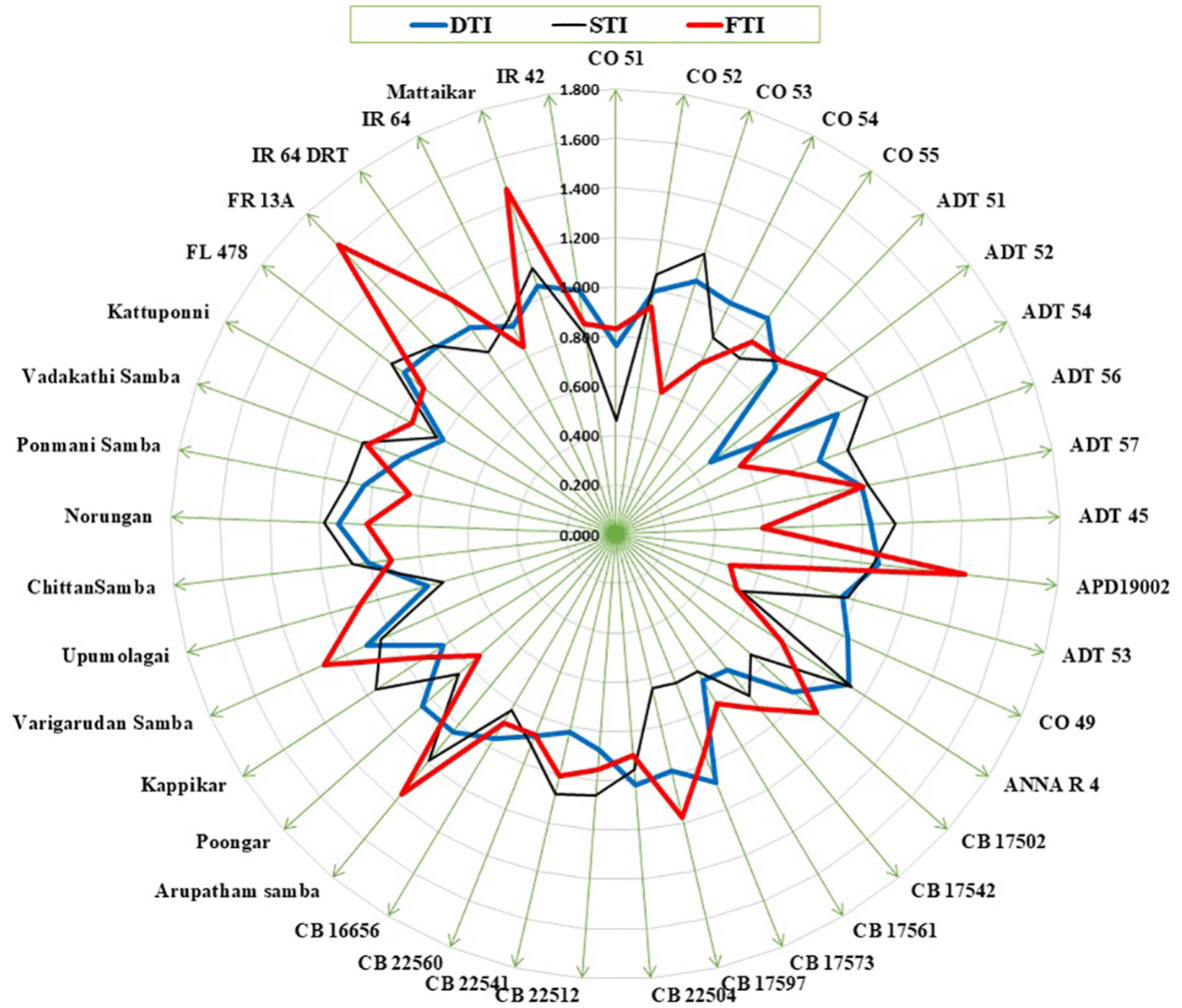
Stress tolerance index for drought, salinity, and submergence under combined seasons.

Plant biomass functioned as the basis for calculating various stress tolerance indices, namely the Drought Tolerance Index (DTI), STI, and Submergence Tolerance Index (FTI). The genotypes with a stress-tolerant index above one over the seasons were considered to be tolerant (**Figure 3**). Over the seasons, nine genotypes (Norungan, APD19002, Anna (R) 4, Arupatham samba, Varigarudan samba, Mattaikar, Ponmani samba, CO53, and Poongar) were found to be superior over the tolerant check IR64Drt1 (1.024), hence these genotypes are grouped as drought tolerant. In the case of salinity, STI varied from 0.460 (CO51) to 1.190 (CO53). Across seasons, the genotypes *viz*., Arupatham samba, CO53, and Norungan exhibited superiority compared to the tolerant check FL478 (1.140), whereas APD19002, Mattaikar, and Varigarudan samba were identified with STI values exceeding 1 in both seasons, classifying them as salinity-tolerant genotypes. Similarly, for submergence, FTI ranged from 0.480 (ADT53) to 1.620 (FR13A) under pooled conditions. Over the seasons, the genotypes, *viz.*, Arupatham samba, Mattaikar, APD19002, and Varigarudan samba, were found to be on par with the tolerant check FR13A (1.620), therefore grouped as submergence tolerant. As per the finding from the Stress-Tolerant Index, the genotypes, *viz.*, Mattaikar, APD19002, Varigarudan samba, and Arupatham samba, were identified to be multiple abiotic stress tolerant.

### Photosystem activities

Photosynthesis activity declined overall under all stress conditions when compared to control. Furthermore, we observed a notable variation among genotypes over the seasons within each stress condition (**Supplementary Table 3**). The RWC and CCI were highly affected under stress conditions, and it was found that tolerant genotypes had higher values of RWC and CCI, indicating the adoptive mechanism to unfavorable conditions.

**Figure 4:**
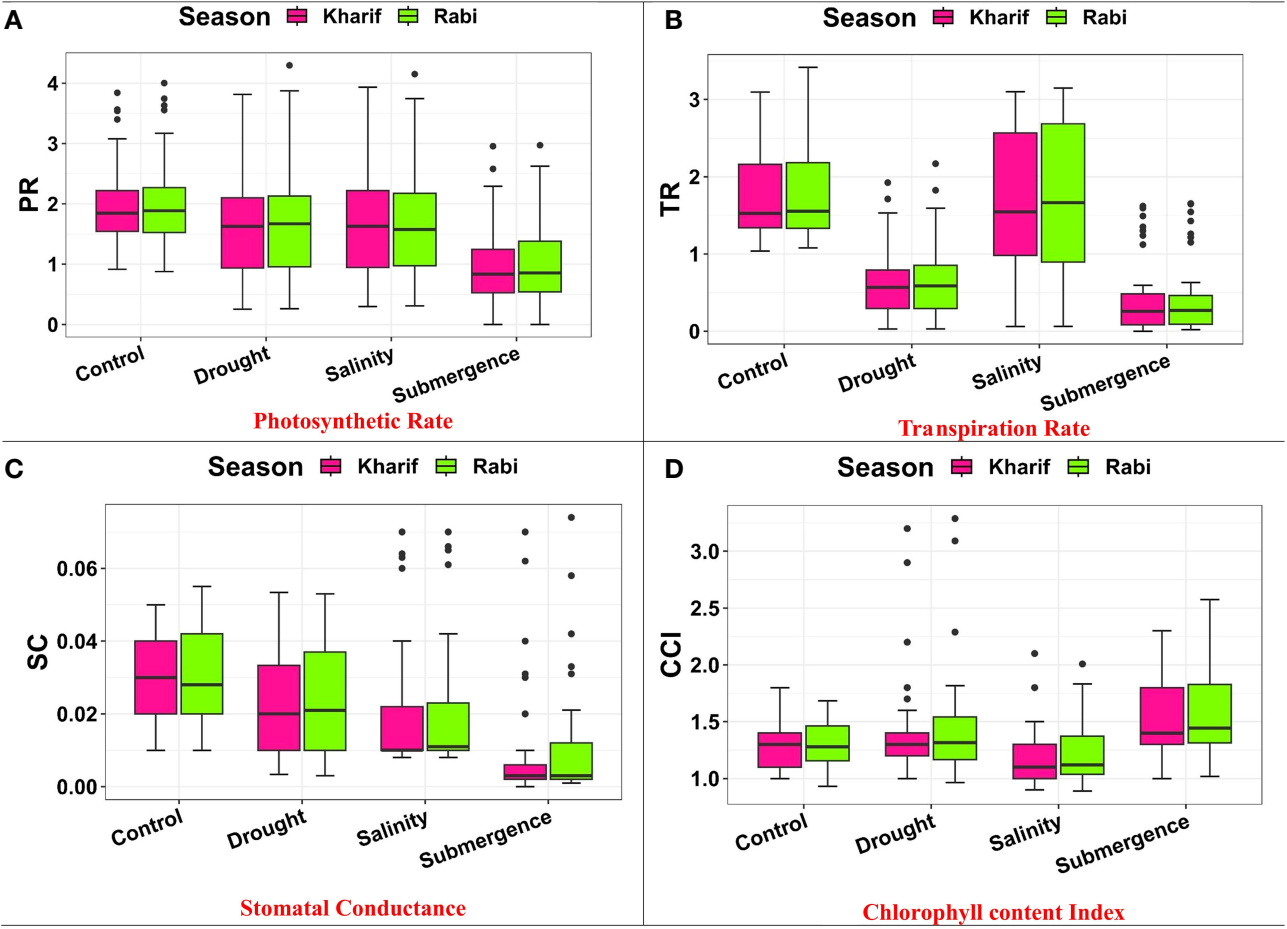
Performance of gas exchange parameters: **(A)** photosynthetic rate, **(B)** transpiration rate, **(C)** stomatal conductance, and **(D)** chlorophyll content index under varied stresses during both seasons.

**Figure 5:**
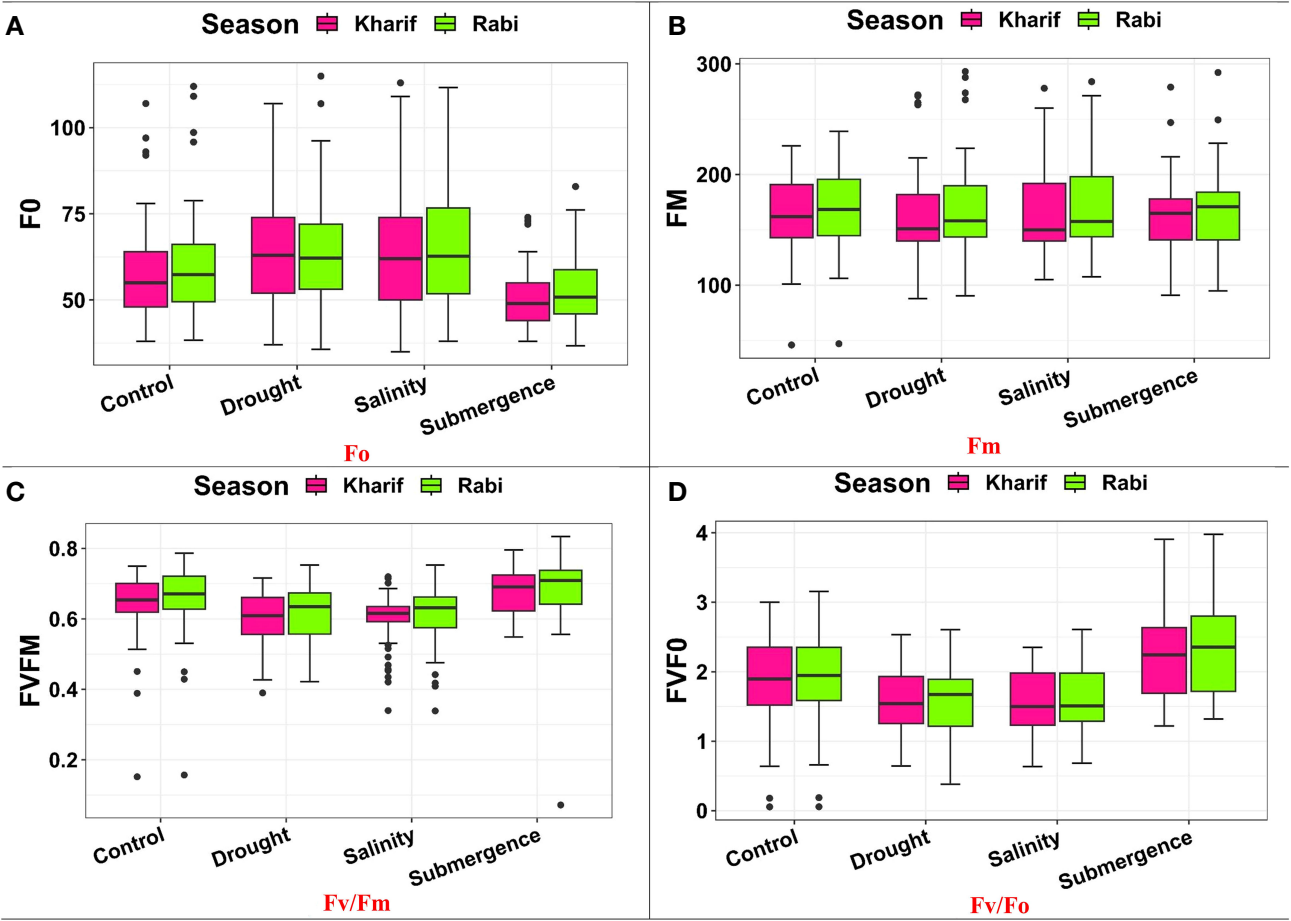
Performance of chlorophyll fluorescence parameters: **(A)** Fo, **(B)** Fm, **(C)** FV/Fm, and **(D)** Fv/Fo under varied stresses during both seasons.

Several leaf gas exchange parameters were measured under all stress conditions. The photosynthetic CO_2_ fixation rate in both seasons decreased under the influence of drought, salinity, and submergence conditions (**Figure 4**). Under pooled conditions, the PN decreased by about 16.394% in drought, 17.434% in salinity, and 50.322% in submergence when compared to the control. The gas exchange parameters, *viz*., gs and *e* were found to be drastically reduced under stress conditions. Stomatal conductance over the season decreased by about 17.241% under drought, 31.034% under salinity, and 62.069% under submergence. Similarly, the *e* also decreased by 63.927% under drought, 5.516% under salinity, and 75.565% under submergence over the seasons. Chlorophyll fluorescence parameters of rice seedlings varied under control and stress conditions. Maximal fluorescence (Fm) and Fv/Fm were significantly (*p*< 0.01) reduced under drought, salinity, and submergence conditions (**Figure 5**). During Kharif, high Fv/Fm was noticed in the genotype Arupatham samba (0.716) under drought, Vadakathi samba (0.720) under salinity, and Mattaikar (0.781) under submergence. Likewise, in Rabi, the genotypes CO51 (0.752), Varigarudan samba (0.753), and FR13A (0.834) exhibited higher Fv/Fm under drought, salinity, and submergence. Likewise, under pooled conditions, the genotypes IR64Drt1 (0.768) under drought, FR13A (0.728) under salinity, and Ponmani samba (0.758) under submergence exhibited higher values for chlorophyll fluorescence, indicating their adoptive mechanisms under unfavorable environment.

### Molecular marker profiling

**Figure 6:**
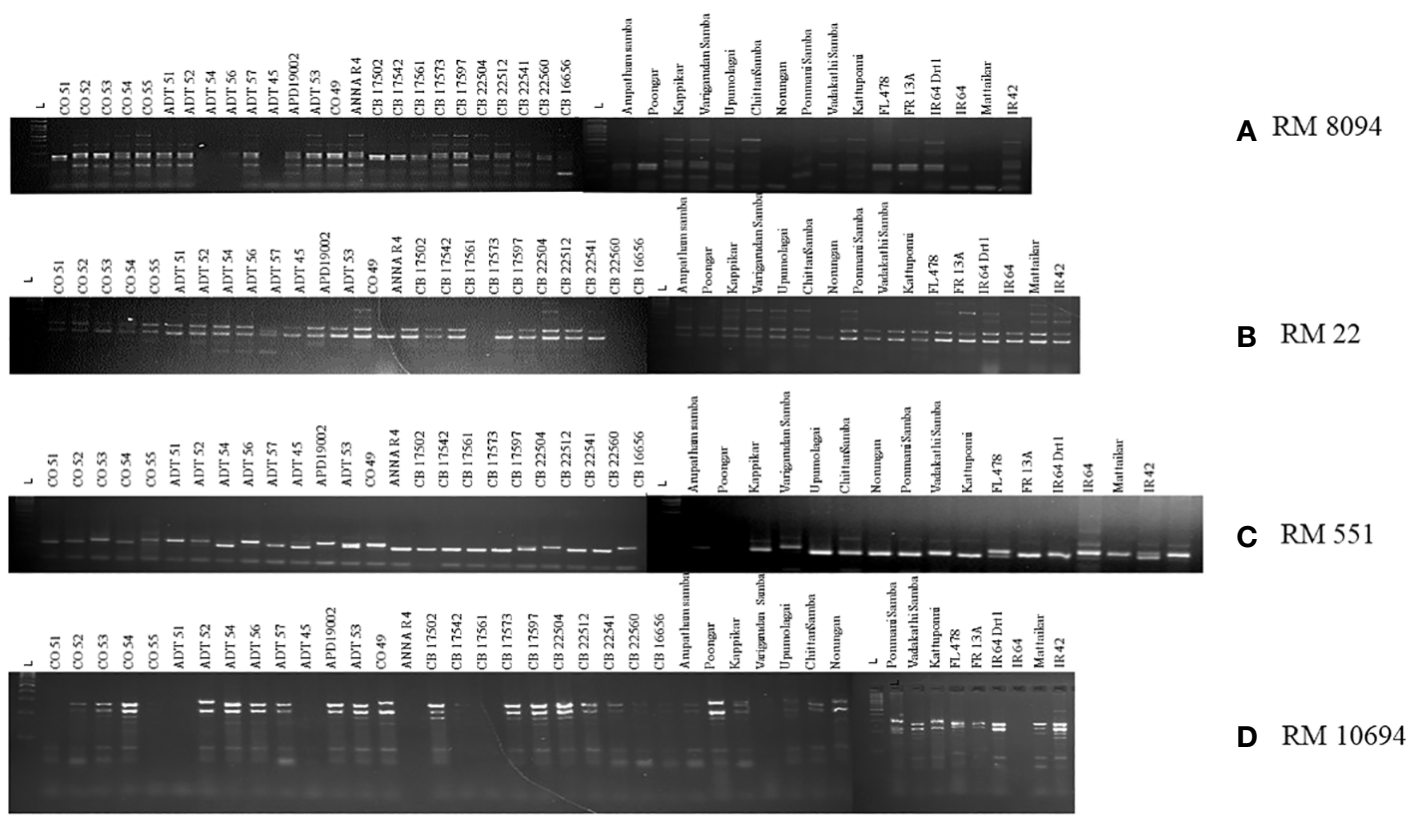
Representation of gel images of linked markers: **(A)** RM8094, **(B)** RM22, **(C)** RM551, and **(D)** RM10694.

SSR marker profiling employing 30 markers identified 28 markers as polymorphic, whereas RM7075 and RM7097 were found to be monomorphic. QTL-specific markers for drought, salinity, and submergence identified 76 loci across 41 rice germplasm with a mean of 2.37 polymorphic bands/marker (**Figure 6**). Maximum polymorphic bands were observed for RM8094 (six bands) followed by RM2634 and RM1287 (five bands). The marker attributes of individual SSR markers were assessed by calculating PIC, MI, RP, and HI (**Table 3**). PIC data varied from 0 to 0.785, with a mean value of 0.37 per primer. The maximum PIC value is obtained by 0.785 (RM8094) followed by RM1287 (0.713) and RM2634 (0.691), and the lowest value is obtained by RM212 (0.124). The HI ranged from 0 to 0.812. HI was found to be at a maximum in RM8094 (0.812), followed by RM1287 (0.755) and RM2634 (0.737), whereas it was lowest in RM316 (0.048) and RM3825 (0.048). The resolving power ranged from 1.85 to 7.805. The resolving power was found to be highest in RM8094 (7.805), followed by RM1287 (6.00) and RM22 (5.70), and the lowest value was observed in SUB_1_BC_3_ (1.561). The marker index ranged from 0 to 4.70. The marker RM8094 showed a higher marker index of 4.70, followed by the markers RM1287 (3.57) and RM2634 (3.45), and the lowest was observed in RM316 and RM3825 (0.047). Pairwise genetic similarity among 41 rice genotypes estimated using the Jaccard similarity coefficient ranged from 0.149 to 0.644 (**Supplementary Table 4**). A high genetic similarity was observed between IR64 and Upumolagai (Jc = 0.644). Likewise, the genetic similarity was observed to be low among the genotypes Kattuponni and CO49 (Jc = 0.149). The average genetic distance among the genotypes was found to be 0.443, indicating the considerable level of genetic diversity within the studied rice germplasm.

**Table 3:** SSR marker attributes of polymorphic information content (PIC), marker index (MI), resolving power (RP), and heterozygous index (HI) in studied rice genotypes.

S. No.	Marker	Total number of band	No. of polymorphic band	Polymorphism percentage	PIC	HI	MI	RP
1	RM211	2	1	50	0.175	0.194	0.175	2.244
2	RM431	2	2	100	0.265	0.314	0.530	2.000
3	RM250	2	1	50	0.198	0.223	0.198	2.293
4	RM212	2	2	100	0.124	0.133	0.248	4.000
5	RM3825	1	1	100	0.047	0.048	0.047	1.951
6	RM22	4	4	100	0.664	0.716	2.657	5.707
7	RM168	3	3	100	0.489	0.564	1.467	2.049
8	RM232	2	2	100	0.371	0.493	0.743	2.000
9	RM551	2	2	100	0.126	0.136	0.253	2.000
10	RM204	2	2	100	0.375	0.500	0.750	2.000
11	RM589	2	2	100	0.375	0.500	0.750	3.902
12	RM520	3	3	100	0.373	0.496	1.119	2.098
13	RM3412	2	2	100	0.371	0.493	0.743	2.000
14	RM11943	2	2	100	0.375	0.500	0.750	2.488
15	RM2634	5	5	100	0.691	0.737	3.457	3.024
16	RM11	2	2	100	0.280	0.336	0.560	2.049
17	RM8094	6	6	100	0.785	0.812	4.708	7.805
18	RM10694	4	4	100	0.637	0.697	2.549	4.878
19	RM140	4	4	100	0.608	0.674	2.434	2.488
20	RM7075	1	0	0	0.000	0.000	0.000	1.951
21	RM1287	5	5	100	0.713	0.755	3.563	6.000
22	RM7097	1	0	0	0.000	0.000	0.000	2.000
23	ART5	2	2	100	0.356	0.464	0.713	2.000
24	SUB1AB1	2	2	100	0.364	0.479	0.728	3.317
25	SUB1BC3	2	2	100	0.545	0.623	1.089	1.561
26	RM219	2	2	100	0.339	0.433	0.679	2.000
27	RM316	1	1	100	0.047	0.048	0.047	1.941
28	RM337	3	3	100	0.463	0.525	1.389	2.390
29	RM104	2	1	50	0.375	0.499	0.375	3.854
30	RM21	3	3	100	0.554	0.624	1.661	2.341
	**Total**	**76**	**71**	**2650**	**0.369**	**0.434**	**1.146**	**2.878**
	**Average**	**2.53**	**2.37**	**88.33**	**0.37**	**0.43**	**1.15**	**2.88**

### Principle coordinate analysis

**Figure 7:**
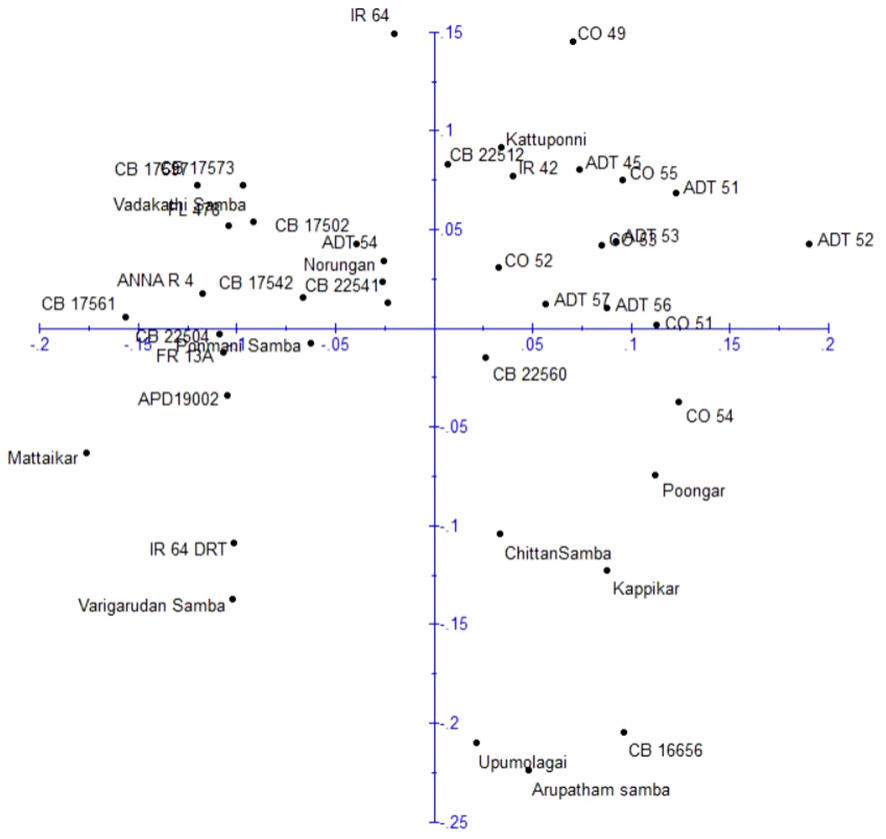
Principle coordinate analysis of rice genotypes based on DARwin analysis.

Principle coordinate analysis (PCoA) was conducted to assess the relationship among rice genotypes based on molecular data (**Figure 7**). The first two principal coordinates collectively accounted for 24.80% of the total variation among the genotypes, with 12.96% attributed to principal coordinate 1 and 11.84% by principal coordinate 2. The distribution of genotypes was analyzed, and they was grouped into four quarters. The first quarter, situated in the top-left region, contained 12 genotypes (IR64, Vadakathi samba, CB17502, CB17573, CB17597, CB17561, CB17542, CB22541, FL478, Anna (R) 4, Norungan, and ADT54). Likewise, the second quarter in the top-right held 14 genotypes (CO49, CO51, CO52, CO53, CO55, ADT45, ADT51, ADT52, ADT53, ADT56, ADT57, CB 22512, IR42, and Kattuponni), the third quarter in the bottom-right contained eight genotypes (CB22560, CO54, Poongar, Chittansamba, Kappikar, CB16656, Upumlagai, and Arupatham samba), and the remaining seven genotypes (CB22504, Ponmani samba, FR13A, APD19002, Mattaikar, IR64Drt1, and Varigarudan samba) were found in the fourth quarter in the bottom-left.

### Population structure analysis

**Figure 8:**
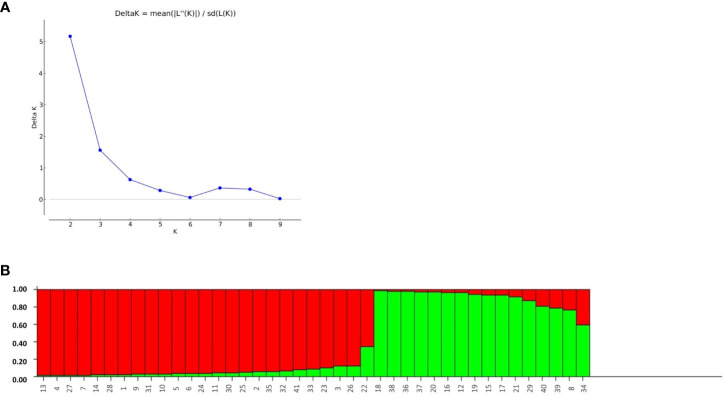
**(A)** Assessment of ad-hoc quantity (Δk) from calculated k and Ln p(D) and **(B)** Bargraph of rice genotypes constructed through population structure analysis.

The structure analysis depicted the classification of the 41 rice genotypes based on their SSR marker profile. The STRUCTURE software was applied with an expectation of *K* = 1, 2, 3…, 10. There were minor differences in consecutive LnP(*D*) values for the SSR marker data. The likelihood score LnP(*D*) progressively improved as *K* increased from 1 to 10, with no distinct peak indicating population assignment. Notably, no Delta-*K* peak was observed after *K* = 2 (**Figure 8A**). The optimal population partition (*K*) was determined to be two subgroups, with subgroup 1 comprising 26 genotypes, showing 23 of pure type and three of admixture. Similarly, subgroup 2 comprises 15 genotypes, with 12 being of pure type and three of admixture. The maximum likelihood score for the population was achieved under these conditions (**Figure 8B**).

### The cluster analysis using the neighbor Jaccard similarity coefficient

**Figure 9:**
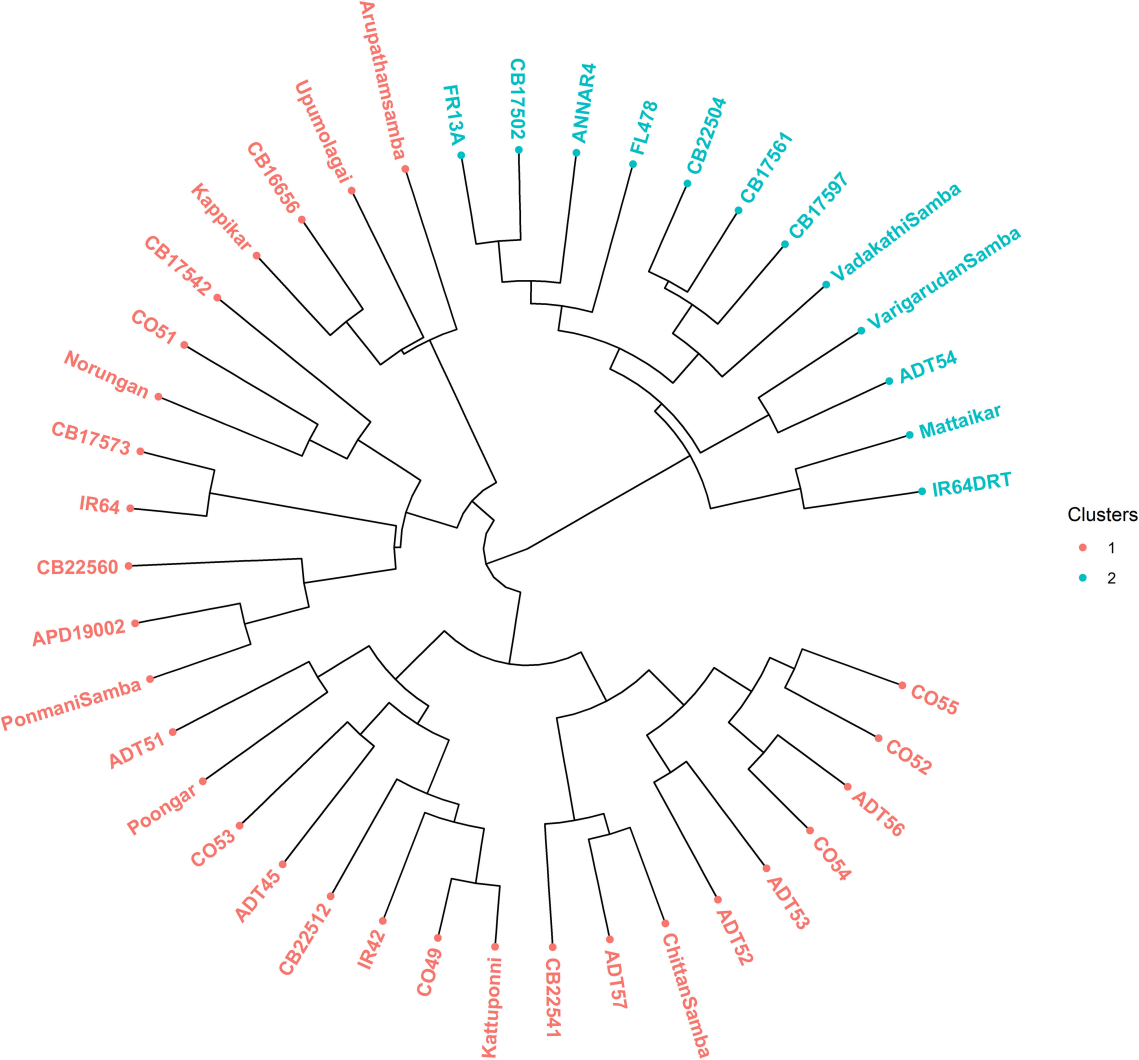
Cluster analysis of rice genotypes based on the Jaccard coefficient dendrogram.

The dendrogram illustrates the clustering of 41 rice genotypes based on their SSR marker profile into two major clusters and six subclusters, which correlated with the population count obtained from the STRUCTURE analysis (**Figure 9**). Major cluster II is the largest, which includes the 29 genotypes comprising susceptible checks IR64 and IR42, along with 12 cultivars, seven prerelease cultures, and eight landraces. Similarly, major cluster I contains 12 genotypes encompassing tolerant checks (IR64Drt1, FL478, and FR13A), two cultivars (ADT54 and Anna (R) 4), four prerelease cultures (CB17502, CB22504, CB17561, and CB17597), and three landraces (Mattaikar, Varigarudan samba, and Vadakathi samba).

### Marker trait association for drought, salinity, and submergence

The genetic associations among 28 polymorphic markers and two plant growth parameters, *viz*., plant biomass and relative shoot length, were analyzed using a single-marker analysis. The significant marker-trait association obtained based on *p*-value (< 0.05) along with their corresponding *R* ^2^ representing the total phenotypic variation accounted for by each marker for multiple abiotic stress tolerance are presented in **Table 4**. Markers associated with plant biomass (PB) and RSL under drought conditions were obtained using 14 drought-linked markers. The makers RM211 and RM212 were found highly associated with plant biomass with an *R* ^2^ value of 0.237 and 0.173, respectively. Likewise, the maker RM551 was found to be significantly associated with relative shoot length, with an *R* ^2^ value of 0.100. In order to find markers associated with traits, *viz*., PB and RSL, with respect to salinity, eight salinity-linked markers were utilized. The results revealed that the marker RM10694 was found to be significantly associated with both traits. Similarly, markers associated with PB and RSL in submergence were analyzed with eight submergence-linked markers, and it was found that the markers RM219 and RM21 were highly associated with plant biomass with *R* ^2^ values of 0.255 and 0.257, respectively, and the marker ART5 was found to significantly associated with relative shoot length with an *R* ^2^ value of 0.193.

**Table 4:** Marker-trait association of studied traits under drought, salinity, and submergence conditions.

Trait	Plant biomass			Relative shoot length		
	Marker	*p*-value	*R* ^2^	Marker	*p*-value	*R* ^2^
**Drought**	RM211	0.006	0.237	RM551	0.05	0.1
	RM212	0.007	0.173			
**Salinity**	RM10694	0.02	0.341	RM10694	0.032	0.319
**Submergence**	RM219	0.001	0.255	ART5	0.004	0.193
	RM21	0.055	0.257			

## Discussion

Rapid changes in climatic conditions have a significant impact on rice yield, and global food demand is increasingly affected by various abiotic stresses (Ramankutty et al., 2018). In the future development of crops, especially within the context of changing climatic conditions, the focus has shifted toward enhancing climate resilience in the creation of climate-smart crops to ensure food and nutritional security (Salgotra and Chauhan, 2023). Multifaceted abiotic stress tolerance takes on substantial importance in high-yield breeding initiatives. Consequently, there is a need to explore physiological variations and genetic diversity at earlier stages (Muthu et al., 2020). This effort aligns with the primary goal of enhancing the capacity for stress resilience and productivity, particularly given the dynamic shifts in environmental conditions. In this study, a diverse panel of rice genotypes, including prerelease lines, cultivars, and landraces, underwent screening under drought, salinity, and submergence conditions to evaluate their performance and identify potential donors for climate-resilient variety development.

**Figure 10:**
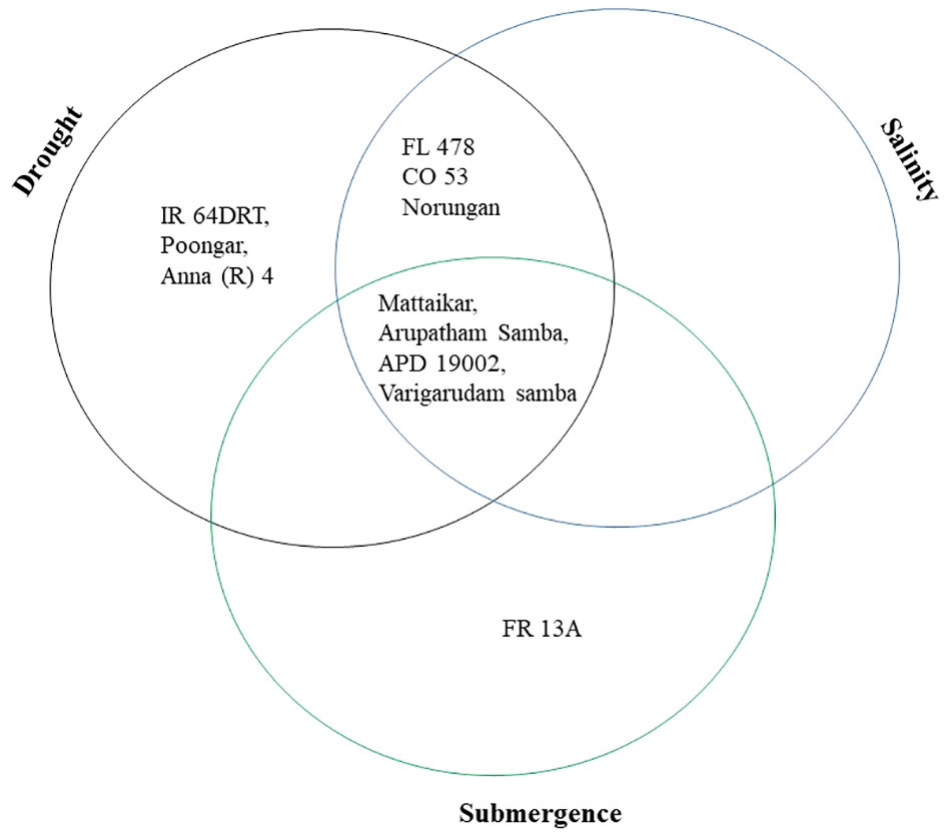
Selection of genotypes based on the stress tolerance index of various abiotic stresses under combined seasons.

The present study demonstrates that the resultant reduction in seedling growth parameters, *viz*., plant biomass and RGI, in both seasons leads to a depletion of dry matter content when the rice genotypes are exposed to subsequent stresses like drought, salinity, and submergence. Under all stress conditions, there was a significant reduction in plant biomass over the season of about 13.253% under drought, 16.466% under salinity, and 26.104% under submergence. Likewise, the relative growth index also showed reductions of 36.900%, 42.00%, and 75.600% under drought, salinity, and submergence, respectively. Similarly, Zhao et al. (2014) reported a 46.700% and 56.800% decline in biomass in FL478 and IR64 under salinity stress conditions. The degree of response of genotypes to stress-induced changes in plant growth varies due to a combination of factors, including genetic variations, duration of stress exposure, and developmental stages (Mundada et al., 2020). The seedling biomass obtained under various stress conditions was employed to derive stress-tolerant indices like DTI, STI, and FTI and was subsequently used for identifying tolerant genotypes. Significant divergence in the values of DTI, STI, and FTI indicted wide diversity among the studied genotypes. Generally, higher values of the stress-tolerant index imply their tolerance nature. As per the finding of Muthuramu and Ragavan (2020), the genotypes (*viz.*, Norungan, APD19002, Anna (R) 4, Arupatham samba, Varigarudan samba, Mattaikar, Ponmani samba, CO53, and Poongar) with a high DTI value and superiority over the tolerant check IR64Drt1 indicate their ability to grow under limited water conditions. Likewise, the genotypes *viz*., Arupatham samba, CO53, and Norungan exhibited superiority compared to the tolerant check FL478, whereas APD19002, Mattaikar, and Varigarudan samba were identified with STI values exceeding 1 in both seasons. High STI signifies their potential to be grown in salt-affected areas. Higher values of FTI in the genotypes, *viz*., Arupatham samba, Mattaikar, APD19002, and Varigarudan samba, were found to be on par with the tolerant check FR13A, indicating their ability to withstand flooding situations. The study revealed that the genotypes, *viz*., Mattaikar, APD19002, Varigarudan samba, and Arupatham samba, were found to be multiple stress tolerant (**Figure 10**). Hence, these genotypes can be employed as potential donors in the development of climate-resilient varieties.

Leaf photosynthesis is an important process in plants and is highly sensitive to various abiotic stresses (Sharma et al., 2020). The results revealed that a gradual reduction in PN was also accompanied by a decrease in *e* and gs in rice seedlings. Under stress conditions, on average, the PN decreased by about 16.394% in drought, 17.434% in salinity, and 50.322% in submergence. Likewise, gs and *e* were found to be drastically reduced under stress conditions. This suggests that stomatal closure upon stress imposition is the major limiting parameter, resulting in declining photosynthesis. This is in accordance with the previous findings of Yamori et al. (2020), where they showed how stomatal conductance affects photosynthesis in rice under changing light conditions using wild-type (WT) rice (*Oryza sativa* L. cv. Taichung 65) and the slac1 mutant (defective in the *OsSLAC1* gene) with a modified stomatal regulation gene (*SLAC1* knockout). Overall, the findings show a considerable impact of drought, salinity, and submergence on key leaf gas exchange parameters, indicating a compromised physiological performance of the plants under these stress conditions in both seasons. The RWC and CCI were highly affected under stress conditions. According to Polash et al. (2018), the accumulation of both organic (proline) and inorganic (K+) osmolytes may play a role in stress alleviation by retaining water in the cells. Fluorescence parameters, *viz*., Fm and Fv/Fm, are excellent ways to evaluate the potential of PSII (Faseela et al., 2020). Under all the stress conditions, we observed an increase in Fo values whereas the Fm and Fv/Fm showed a declining trend, suggesting the alteration of PSII activity and photo-inhibition under various stress conditions. Similar results were obtained by Muhammad et al. (2021) for drought, Tsai et al. (2019) for salinity, and Elanchezhian et al. (2015) for submergence. The ability of genotypes to uphold photosynthetic rate and PSII activity determines their tolerance potentiality (Sarkar and Ray, 2016; Pradhan et al., 2019). As a result, the genotypes Mattaikar, Arupatham samba, Varigarudan samba, and Vadakathi samba were identified with higher PSII activity under stress conditions, ultimately leading to better photosynthesis and growth. Based on our experiments, it appears that physiological efficiency is the most indicative parameter for distinguishing genotype responses. Genotypes exhibiting higher physiological efficiency are more likely to tolerate stress conditions, whereas those with lower physiological efficiency are more susceptible.

SSR markers tightly linked to QTLs are amazing molecular tools for the genetic profiling of rice accessions and the identification of tolerant genotypes under multiple abiotic stress conditions. In this study, the genetic diversity of 41 rice genotypes was evaluated by employing 30 markers associated with the target trait. A substantial level of polymorphism was identified in the majority of the SSR markers. A similar result was found in Ram et al. (2007), who assessed genetic diversity and revealed the considerable allelic variability among SSR markers encompassing rice germplasm into cultivars, landraces, and wild relatives, providing valuable insights into genetic variability for future utilization. Based on marker profiling, the SSR primer RM8094, RM1287, RM2634, RM22, RM10694, and RM140 displayed higher values for PIC, MI, and RP, suggesting the potential of further utilization of these markers in investigating genetic diversity of rice accessions. Likewise, Lokeshkumar et al. (2023) identified three landraces (Kuttimanja, Tulasimog, and IET-13713I) as salt-tolerant with strong correlations in morphological and physiological traits under various conditions. Similarly, based on molecular analysis of the *Salto*l region, the markers *viz.*, AP3206F, RM10793, and RM3412b, located close to the *SKC1* gene (11.23–12.55 Mb), displayed new alleles in tolerant lines like Kuttimanja, IET-13713I, and Tulasimog, suggesting their potential as candidates for novel genomic regions associated with salinity tolerance, whereas using high-yielding *indica* rice variety as a donor to developing multiple stress-tolerant rice variety through marker-assisted selection by Ali et al. (2017).

The average genetic distance among the genotypes was 0.443, suggesting an elevated magnitude of genetic diversity among the studied rice genotypes, and maximal genetic similarity was observed in the pairwise comparison between germplasm IR64 and Upumolagai. Whereas, the landraces are more genetically diverse than prerelease lines and cultivars. Despite diverse genotypes, rice landraces and cultivars exhibit tolerance to drought, salinity, and submergence. An earlier study of a diverse panel of 148 rice accessions, including 47 cultivars, 59 landraces from Taiwan, and 42 from other countries revealed five subpopulations. Genetic diversity ranked higher in wild rice than in landraces and cultivars. These landraces exhibited significant genetic diversification, offering a valuable reservoir for future rice breeding (Hour et al., 2020). All genotypes were grouped into two major and seven subclusters by the Jaccard cluster analysis (**Figure 9**). Furthermore, the identification of tolerant groups was done using their respective tolerant and susceptible checks. Eight rice genotypes, such as CB22504, CB17561, CB17597, CB17502, Vadakathi samba, Varigarudan samba, Mattaikar, and ADT54, were identified to be multiple abiotic stress tolerant as they are grouped together with all the tolerant checks, *viz*., Anna (R) 4 and IR64Drt1 (drought), FL478 (salinity), and FR13A (submergence). A similar grouping pattern was observed in aromatic rice landraces under multiple abiotic stress conditions by Behera et al. (2023). Genotypic diversity attributed to stress tolerance QTLs varies among varieties and is highly affected by the environment (Gaballah et al., 2021). Results from this study identified that the salt-tolerant check (FL478) and submergence-tolerant check (FR13A) are genetically close. It was also reported that FR13A can be a donor for novel alleles, and FR13A can be a donor for new alleles imparting salinity and drought resistance. An earlier study showed that *Porteresia coarctata*, a wild relative of rice, exhibits high salinity and submergence tolerance. Through transcriptome analysis encompassing 375 million reads, 152,367 unique transcripts, including stress-responsive genes and 2,749 transcription factors, were identified. Likewise, key pathways in amino acid and hormone biosynthesis, secondary metabolite biosynthesis, carbohydrate metabolism, and cell wall structures contribute to stress tolerance (Garg et al., 2014). These findings provide insight into the genetic mechanisms of *Porteresia’s* tolerance, offering potential strategies for engineering salinity and submergence tolerance in rice. An earlier study reported that genetic network induced in response to submergence and drought tolerance might share some common transcriptional factors at the gene expression level (Fukao et al., 2011), and engineering common transcription factors can ultimately lead to higher multiple stress tolerance (Manna et al., 2021). The genotypes that are identified as genetically close to IR64Drt1, FL478, and FR13A can be used as potential donors for developing climate resilience cultivars (Muthu et al., 2020).

STRUCTURE analysis revealed a very broad genetic base (*K* = 2) while employing different SSR markers linked to multiple abiotic stress-tolerant QTLs. The higher *K* value obtained in this study depicts the diverse nature of the population (**Figure 8A**). Our results were in comparison with the earlier reports of Kimwemwe et al. (2023), who assessed the genetic diversity and population structure of 94 rice genotypes using DArT-based SNP markers. We observed an average PIC of 0.25, identified five subpopulations (*K* = 5), and found a high average Euclidean genetic distance of 0.87, indicating the existence genetic diversity. The level of genetic diversity found in our study may help to select and conserve rice landraces. Thus, the rice landraces associated with multiple abiotic stress tolerance may be used as parental material in rice breeding to manage rice production in a changing climate.

Marker-assisted breeding programs emphasize the importance of establishing strong marker-trait associations in order to effectively utilize specific markers for trait enhancement (Lakshmi et al., 2021). The genetic associations among 13 polymorphic drought-linked markers and two plant growth parameters, *viz*., plant biomass and relative shoot length, analyzed using a single-marker analysis revealed RM211 and RM212 are closely associated with plant biomass under water-limiting conditions whereas RM551 was closely linked to relative shoot length under water-limiting conditions. Likewise, Salunkhe et al. (2011) reported that RM212, RM302, RM8085, and RM3825 exerted a substantial influence on drought-resistant traits. Similarly, the marker-trait association employing eight salinity-linked polymorphic markers identified RM10694 to be closely linked to plant biomass and relative shoot length under saline conditions. The results are in concordance with Kumari et al. (2019) and Volkova (2017), in which they stated the markers, *viz*., RM302, RM8094, RM10665, RM10694, RM10748, and RM10825, can be employed in validating QTLs for salinity tolerance. The single marker analysis employing seven submergence-linked markers identified RM219 and RM21 to be significantly associated with plant biomass under submergence, whereas ART5 was found to be associated with relative shoot length under submergence. It is worth noting that similar markers (RM219, RM21, and ART5) were reported to be closely linked to submergence, as per earlier findings of Islam et al. (2008); Biswas et al. (2013), and Muthu et al. (2020). Therefore, the abovementioned marker-trait associations could be employed in the identification of tolerant lines in future breeding programs aimed at developing multiple abiotic stress-tolerant varieties.

## Conclusion

In the face of climate change and global warming, developing and utilizing genotypes with tolerance to multiple abiotic stresses is of great significance, as it has the potential to boost food production and ensure the stability of rice cultivation. The findings of this study present an opportunity to improve rice cultivars with multiple abiotic stress tolerance, as these germplasms exhibit a wider genetic diversity related to traits enabling them to withstand challenges like drought, salinity, and submergence. Genotypic analysis involving 30 SSR markers revealed substantial genetic similarity among all the studied rice genotypes, indicating a significant level of genetic diversity within the population. STRUCTURE analysis revealed a broad genetic base (K = 2), further emphasizing the suitability of these rice genotypes for coping with environmental stresses. The marker-trait associations suggest that markers RM211, RM212, RM10694, RM219, RM21, and ART5 could be useful for evaluating trait-specific multiple abiotic stress tolerance. The genotypes APD19002, Mattaikar, Varigarudan samba, and Arupatham samba are considered important genetic resources as they exhibit multiple stress tolerance making them a potential donor to be employed in stress resilience breeding.

## Data availability statement

The original contributions presented in the study are included in the article/**Supplementary Material**. Further inquiries can be directed to the corresponding author.

## Author contributions

KP: Data curation, Methodology, Software, Visualization, Writing – original draft. RP: Conceptualization, Investigation, Methodology, Resources, Supervision, Writing – review & editing. SM: Investigation, Resources, Writing – review & editing. MR: Writing – review & editing. SS: Data curation, Software, Writing – review & editing. AS: Investigation, Writing – review & editing.
