# Supplementary material for: Enhancing stress resilience in rice (Oryza sativa L.) through profiling early-stage morpho-physiological and molecular responses to multiple abiotic stress tolerance
Source: Front Plant Sci. 2024 Feb 8;15:1342441. doi: 10.3389/fpls.2024.1342441 (PMC10882102; doi:10.3389/fpls.2024.1342441)
Supplement: Supplementary file 1 [file Table_1.docx]

**Supplementary table 1. List of genotypes**

| **S No** | **Genotype** | **Parentage** | **types** | **S No** | **Genotype** | **Parentage** | **types** |
| --- | --- | --- | --- | --- | --- | --- | --- |
| **1** | CO 51 | ADT 43/ RR 272-1745 | Cultivar | **22** | CB 22512 | CO 54 X CB 13805 | Pre-release culture |
| **2** | CO 52 | BPT 5204 and CO(R) 50 | Cultivar | **23** | CB 22541 | CO 54 X CB 13804 | Pre-release culture |
| **3** | CO 53 | PMK (R ) 3 x Norungan | Cultivar | **24** | CB 22560 | CO 54 X 13806 | Pre-release culture |
| **4** | CO 54 | CB 04110 / CB 05501 | Cultivar | **25** | CB 16656 | CB 08504 X 11T21588 | Pre-release culture |
| **5** | CO 55 | ADT 43 x GEB 24 | Cultivar | **26** | Arupatham samba | - | Landrace |
| **6** | ADT 51 | BPT 5204 / Improved White ponni | Cultivar | **27** | Poongar | - | Landrace |
| **7** | ADT 52 | CR 1009 / ADT 49 | Cultivar | **28** | Kappikar | - | Landrace |
| **8** | ADT 54 | I.W.Ponni / Banskathi | Cultivar | **29** | Varigarudan Samba | - | Landrace |
| **9** | ADT 56 | WGL 14377 / MDU 5 | Cultivar | **30** | Upumolagai | - | Landrace |
| **10** | ADT 57 | ADT (R) 45 / ACK 03002 | Cultivar | **31** | ChittanSamba | - | Landrace |
| **11** | ADT 45 | IR 50/ADT 37 | Cultivar | **32** | Norungan | - | Landrace |
| **12** | APD19002 | 36-27-2 X CBMAS 14065 | Pre-release culture | **33** | Ponmani Samba | - | Landrace |
| **13** | ADT 53 | ADT 43/JGL 384 | Cultivar | **34** | Vadakathi Samba | - | Landrace |
| **14** | CO 49 | C 20 / RNR 52147 | Cultivar | **35** | Kattuponni | - | Landrace |
| **15** | ANNA R 4 | Pantdhan 10 X IET 9911) | Cultivar | **36** | FL 478 (Saltol check) | IR 29 X Pokkali | Cultivar |
| **16** | CB 17502 | CO 51 X Luanheden | Pre-release culture | **37** | FR 13A (Sub check) | Landrace | Cultivar |
| **17** | CB 17542 | C0 41 XC0 51 | Pre-release culture | **38** | IR 64 DRT (drt check) | - | Cultivar |
| **18** | CB 17561 | CO 41 X CB 08504 | Pre-release culture | **39** | IR 64(suscep.check) | IR 5657-33-2-1/IR 2061-465-1-5-3 | Cultivar |
| **19** | CB 17573 | CO 1 X EC 725255 | Pre-release culture | **40** | Mattaikar | - | Landrace |
| **20** | CB 17597 | ARD 6 X CO 41 | Pre-release culture | **41** | IR 42(suscep.check) | - | Cultivar |
| **21** | CB 22504 | CO 51 X CB 13805 | Pre-release culture |  | | | |
