# Supplementary material for: Enhancing stress resilience in rice (Oryza sativa L.) through profiling early-stage morpho-physiological and molecular responses to multiple abiotic stress tolerance
Source: Front Plant Sci. 2024 Feb 8;15:1342441. doi: 10.3389/fpls.2024.1342441 (PMC10882102; doi:10.3389/fpls.2024.1342441)
Supplement: Supplementary file 2 [file Table_2.docx]

Supplementary table 2. Morphology traits

| **Traits** | **Plant Biomass** | | | | | | | | | | | |
| --- | --- | --- | --- | --- | --- | --- | --- | --- | --- | --- | --- | --- |
|  | **Control** | | | **Drought** | | | **Salinity** | | | **Submergence** | | |
| **Seasons** | **Kharif** | **Rabi** | **Pooled** | **Kharif** | **Rabi** | **Pooled** | **Kharif** | **Rabi** | **Pooled** | **Kharif** | **Rabi** | **Pooled** |
| ADT 45 | 0.189 | 0.181 | 0.185 | 0.149 | 0.185 | 0.167 | 0.156 | 0.192 | 0.174 | 0.161 | 0.016 | 0.088 |
| ADT 51 | 0.221 | 0.222 | 0.221 | 0.198 | 0.219 | 0.208 | 0.184 | 0.237 | 0.210 | 0.180 | 0.235 | 0.207 |
| ADT 52 | 0.240 | 0.244 | 0.242 | 0.171 | 0.037 | 0.104 | 0.204 | 0.251 | 0.227 | 0.197 | 0.248 | 0.223 |
| ADT 53 | 0.150 | 0.155 | 0.152 | 0.131 | 0.169 | 0.150 | 0.121 | 0.149 | 0.135 | 0.103 | 0.012 | 0.058 |
| ADT 54 | 0.218 | 0.209 | 0.214 | 0.161 | 0.220 | 0.191 | 0.172 | 0.238 | 0.205 | 0.156 | 0.033 | 0.095 |
| ADT 56 | 0.302 | 0.340 | 0.321 | 0.205 | 0.290 | 0.248 | 0.249 | 0.289 | 0.269 | 0.256 | 0.083 | 0.169 |
| ADT 57 | 0.291 | 0.300 | 0.295 | 0.207 | 0.321 | 0.264 | 0.235 | 0.281 | 0.258 | 0.217 | 0.294 | 0.256 |
| ANNA R 4 | 0.239 | 0.231 | 0.235 | 0.229 | 0.233 | 0.231 | 0.197 | 0.248 | 0.222 | 0.249 | 0.043 | 0.146 |
| APD19002 | 0.328 | 0.358 | 0.343 | 0.300 | 0.343 | 0.322 | 0.314 | 0.289 | 0.302 | 0.319 | 0.127 | 0.223 |
| Arupatham samba | 0.348 | 0.335 | 0.341 | 0.323 | 0.279 | 0.301 | 0.313 | 0.354 | 0.333 | 0.317 | 0.135 | 0.226 |
| CB 16656 | 0.231 | 0.254 | 0.243 | 0.214 | 0.244 | 0.229 | 0.205 | 0.165 | 0.185 | 0.160 | 0.239 | 0.200 |
| CB 17502 | 0.180 | 0.190 | 0.185 | 0.127 | 0.187 | 0.157 | 0.146 | 0.083 | 0.114 | 0.182 | 0.178 | 0.180 |
| CB 17542 | 0.099 | 0.109 | 0.104 | 0.089 | 0.072 | 0.081 | 0.093 | 0.105 | 0.099 | 0.075 | 0.103 | 0.089 |
| CB 17561 | 0.124 | 0.197 | 0.161 | 0.091 | 0.093 | 0.092 | 0.077 | 0.092 | 0.084 | 0.076 | 0.092 | 0.084 |
| CB 17573 | 0.152 | 0.157 | 0.154 | 0.131 | 0.163 | 0.147 | 0.117 | 0.052 | 0.085 | 0.107 | 0.167 | 0.137 |
| CB 17597 | 0.109 | 0.100 | 0.105 | 0.093 | 0.093 | 0.093 | 0.076 | 0.041 | 0.059 | 0.074 | 0.088 | 0.081 |
| CB 22504 | 0.229 | 0.238 | 0.233 | 0.215 | 0.254 | 0.234 | 0.191 | 0.232 | 0.211 | 0.160 | 0.252 | 0.206 |
| CB 22512 | 0.200 | 0.207 | 0.204 | 0.178 | 0.134 | 0.156 | 0.166 | 0.196 | 0.181 | 0.167 | 0.197 | 0.182 |
| CB 22541 | 0.193 | 0.193 | 0.193 | 0.164 | 0.115 | 0.139 | 0.159 | 0.189 | 0.174 | 0.135 | 0.212 | 0.173 |
| CB 22560 | 0.211 | 0.223 | 0.217 | 0.180 | 0.214 | 0.197 | 0.183 | 0.213 | 0.198 | 0.146 | 0.217 | 0.182 |
| ChittanSamba | 0.198 | 0.205 | 0.202 | 0.167 | 0.190 | 0.179 | 0.164 | 0.197 | 0.181 | 0.170 | 0.197 | 0.183 |
| CO 49 | 0.190 | 0.194 | 0.192 | 0.141 | 0.205 | 0.173 | 0.140 | 0.043 | 0.092 | 0.137 | 0.209 | 0.173 |
| CO 51 | 0.288 | 0.300 | 0.294 | 0.203 | 0.315 | 0.259 | 0.204 | 0.027 | 0.115 | 0.228 | 0.062 | 0.145 |
| CO 52 | 0.208 | 0.217 | 0.213 | 0.166 | 0.206 | 0.186 | 0.176 | 0.204 | 0.190 | 0.153 | 0.224 | 0.188 |
| CO 53 | 0.321 | 0.324 | 0.323 | 0.292 | 0.248 | 0.270 | 0.301 | 0.342 | 0.322 | 0.269 | 0.035 | 0.152 |
| CO 54 | 0.340 | 0.351 | 0.345 | 0.289 | 0.345 | 0.317 | 0.298 | 0.217 | 0.257 | 0.288 | 0.022 | 0.155 |
| CO 55 | 0.208 | 0.197 | 0.203 | 0.182 | 0.203 | 0.192 | 0.180 | 0.124 | 0.152 | 0.174 | 0.037 | 0.106 |
| FL 478 | 0.299 | 0.310 | 0.305 | 0.271 | 0.304 | 0.287 | 0.266 | 0.316 | 0.291 | 0.243 | 0.041 | 0.142 |
| FR 13A | 0.291 | 0.317 | 0.304 | 0.245 | 0.325 | 0.285 | 0.254 | 0.296 | 0.275 | 0.350 | 0.296 | 0.323 |
| IR 42 | 0.198 | 0.206 | 0.202 | 0.163 | 0.199 | 0.181 | 0.169 | 0.114 | 0.141 | 0.141 | 0.203 | 0.172 |
| IR 64 | 0.166 | 0.158 | 0.162 | 0.133 | 0.161 | 0.147 | 0.135 | 0.155 | 0.145 | 0.117 | 0.026 | 0.072 |
| IR 64 DRT | 0.296 | 0.306 | 0.301 | 0.265 | 0.276 | 0.271 | 0.261 | 0.196 | 0.229 | 0.205 | 0.325 | 0.265 |
| Kappikar | 0.323 | 0.306 | 0.314 | 0.277 | 0.196 | 0.237 | 0.272 | 0.354 | 0.313 | 0.239 | 0.320 | 0.280 |
| Kattuponni | 0.309 | 0.307 | 0.308 | 0.262 | 0.165 | 0.214 | 0.262 | 0.165 | 0.214 | 0.217 | 0.312 | 0.265 |
| Mattaikar | 0.350 | 0.358 | 0.354 | 0.334 | 0.320 | 0.327 | 0.329 | 0.343 | 0.336 | 0.345 | 0.336 | 0.341 |
| Norungan | 0.352 | 0.344 | 0.348 | 0.345 | 0.341 | 0.343 | 0.316 | 0.372 | 0.344 | 0.272 | 0.329 | 0.300 |
| Ponmani Samba | 0.260 | 0.269 | 0.264 | 0.232 | 0.250 | 0.241 | 0.216 | 0.273 | 0.244 | 0.186 | 0.258 | 0.222 |
| Poongar | 0.382 | 0.417 | 0.399 | 0.336 | 0.399 | 0.368 | 0.333 | 0.238 | 0.285 | 0.341 | 0.093 | 0.217 |
| Upumolagai | 0.260 | 0.271 | 0.266 | 0.222 | 0.145 | 0.183 | 0.211 | 0.114 | 0.162 | 0.230 | 0.252 | 0.241 |
| Vadakathi Samba | 0.258 | 0.286 | 0.272 | 0.237 | 0.196 | 0.217 | 0.212 | 0.287 | 0.249 | 0.184 | 0.032 | 0.108 |
| Varigarudan Samba | 0.282 | 0.289 | 0.285 | 0.267 | 0.286 | 0.277 | 0.263 | 0.238 | 0.250 | 0.273 | 0.308 | 0.291 |
| **Mean** | **0.245** | **0.253** | **0.249** | **0.209** | **0.223** | **0.216** | **0.208** | **0.208** | **0.208** | **0.200** | **0.168** | **0.184** |
| **LSD** | **0.027** | **0.010** | **0.026** | **0.116** | **0.011** | **0.078** | **0.111** | **0.011** | **0.086** | **0.147** | **0.010** | **0.172** |
| **CV** | **6.679** | **3.495** | **5.140** | **34.049** | **3.149** | **17.962** | **33.030** | **3.236** | **20.552** | **45.145** | **3.495** | **46.210** |
| **SE** | **0.009** | **0.005** | **0.009** | **0.041** | **0.004** | **0.028** | **0.040** | **0.004** | **0.030** | **0.052** | **0.003** | **0.060** |
| **Percent Reduction** | **0.000** | **0.000** | **0.000** | **14.458** | **11.892** | **13.253** | **15.105** | **17.944** | **16.466** | **18.272** | **33.587** | **26.104** |

| **Traits** | **Relative shoot length** | | | | | | | | | | | |
| --- | --- | --- | --- | --- | --- | --- | --- | --- | --- | --- | --- | --- |
|  | **Control** | | | **Drought** | | | **Salinity** | | | **Submergence** | | |
| **Seasons** | **Kharif** | **Rabi** | **Pooled** | **Kharif** | **Rabi** | **Pooled** | **Kharif** | **Rabi** | **Pooled** | **Kharif** | **Rabi** | **Pooled** |
| ADT 45 | 1.000 | 1.000 | 1.000 | 1.090 | 0.878 | 0.984 | 0.919 | 0.891 | 0.909 | 1.055 | 1.137 | 1.096 |
| ADT 51 | 1.000 | 1.000 | 1.000 | 1.004 | 0.992 | 0.998 | 1.204 | 0.868 | 1.036 | 1.003 | 1.023 | 1.013 |
| ADT 52 | 1.000 | 1.000 | 1.000 | 0.737 | 0.940 | 0.839 | 1.020 | 0.961 | 0.990 | 0.958 | 1.023 | 0.991 |
| ADT 53 | 1.000 | 1.000 | 1.000 | 1.044 | 1.002 | 1.023 | 1.123 | 0.940 | 1.032 | 1.046 | 0.982 | 1.014 |
| ADT 54 | 1.000 | 1.000 | 1.000 | 0.780 | 0.816 | 0.798 | 1.107 | 0.940 | 1.024 | 0.863 | 1.085 | 0.974 |
| ADT 56 | 1.000 | 1.000 | 1.000 | 0.950 | 0.982 | 0.966 | 1.027 | 0.971 | 0.999 | 1.006 | 1.013 | 1.009 |
| ADT 57 | 1.000 | 1.000 | 1.000 | 0.757 | 0.878 | 0.818 | 0.946 | 0.951 | 0.948 | 0.885 | 1.095 | 0.990 |
| ANNA R 4 | 1.000 | 1.000 | 1.000 | 0.836 | 1.054 | 0.945 | 1.050 | 1.147 | 1.098 | 0.976 | 0.971 | 0.974 |
| APD19002 | 1.000 | 1.000 | 1.000 | 0.916 | 1.044 | 0.980 | 0.918 | 1.044 | 0.981 | 0.877 | 0.889 | 0.883 |
| Arupatham samba | 1.000 | 1.000 | 1.000 | 0.706 | 1.054 | 0.880 | 0.790 | 0.909 | 0.850 | 0.866 | 0.878 | 0.872 |
| CB 16656 | 1.000 | 1.000 | 1.000 | 0.788 | 0.889 | 0.838 | 0.719 | 0.785 | 0.752 | 0.928 | 0.93 | 0.929 |
| CB 17502 | 1.000 | 1.000 | 1.000 | 0.961 | 0.909 | 0.935 | 0.963 | 0.964 | 0.967 | 1.129 | 0.992 | 1.060 |
| CB 17542 | 1.000 | 1.000 | 1.000 | 1.094 | 0.847 | 0.971 | 1.269 | 1.219 | 1.244 | 1.279 | 0.971 | 1.125 |
| CB 17561 | 1.000 | 1.000 | 1.000 | 0.760 | 0.765 | 0.762 | 1.177 | 1.147 | 1.162 | 0.843 | 0.951 | 0.897 |
| CB 17573 | 1.000 | 1.000 | 1.000 | 0.627 | 0.723 | 0.675 | 1.034 | 1.095 | 1.065 | 1.055 | 0.982 | 1.019 |
| CB 17597 | 1.000 | 1.000 | 1.000 | 0.976 | 1.116 | 1.046 | 1.289 | 1.436 | 1.363 | 1.218 | 0.961 | 1.089 |
| CB 22504 | 1.000 | 1.000 | 1.000 | 0.923 | 0.971 | 0.947 | 1.062 | 1.085 | 1.073 | 1.091 | 0.878 | 0.984 |
| CB 22512 | 1.000 | 1.000 | 1.000 | 0.846 | 0.868 | 0.857 | 1.177 | 1.178 | 1.178 | 1.066 | 0.94 | 1.003 |
| CB 22541 | 1.000 | 1.000 | 1.000 | 0.872 | 0.909 | 0.890 | 1.074 | 1.095 | 1.084 | 0.716 | 0.971 | 0.911 |
| CB 22560 | 1.000 | 1.000 | 1.000 | 0.920 | 1.033 | 0.976 | 0.894 | 0.971 | 0.933 | 1.137 | 0.92 | 1.028 |
| ChittanSamba | 1.000 | 1.000 | 1.000 | 1.033 | 1.064 | 1.049 | 1.059 | 1.064 | 1.062 | 1.184 | 0.94 | 1.062 |
| CO 49 | 1.000 | 1.000 | 1.000 | 0.960 | 0.971 | 0.966 | 1.189 | 0.909 | 1.049 | 0.650 | 0.94 | 1.012 |
| CO 51 | 1.000 | 1.000 | 1.000 | 0.845 | 0.982 | 0.913 | 0.937 | 0.920 | 0.928 | 1.039 | 1.054 | 1.046 |
| CO 52 | 1.000 | 1.000 | 1.000 | 0.886 | 0.930 | 0.908 | 0.944 | 0.940 | 0.942 | 0.710 | 1.064 | 0.970 |
| CO 53 | 1.000 | 1.000 | 1.000 | 0.840 | 1.044 | 0.942 | 1.094 | 1.168 | 1.131 | 1.030 | 0.909 | 0.969 |
| CO 54 | 1.000 | 1.000 | 1.000 | 1.053 | 0.982 | 1.018 | 0.934 | 0.741 | 0.932 | 1.030 | 0.951 | 0.990 |
| CO 55 | 1.000 | 1.000 | 1.000 | 0.984 | 0.992 | 0.988 | 0.807 | 0.764 | 0.848 | 1.078 | 0.971 | 1.025 |
| FL 478 | 1.000 | 1.000 | 1.000 | 0.973 | 1.106 | 1.039 | 0.946 | 1.044 | 0.995 | 1.161 | 0.961 | 1.061 |
| FR 13A | 1.000 | 1.000 | 1.000 | 0.672 | 0.930 | 0.801 | 0.836 | 1.116 | 0.976 | 0.768 | 0.827 | 0.797 |
| IR 42 | 1.000 | 1.000 | 1.000 | 0.914 | 0.982 | 0.948 | 0.913 | 0.971 | 0.942 | 0.654 | 1.002 | 1.061 |
| IR 64 | 1.000 | 1.000 | 1.000 | 0.722 | 0.806 | 0.764 | 0.863 | 0.940 | 0.902 | 0.710 | 1.002 | 1.123 |
| IR 64 DRT | 1.000 | 1.000 | 1.000 | 1.118 | 1.085 | 1.101 | 1.001 | 0.940 | 0.971 | 1.010 | 1.002 | 1.006 |
| Kappikar | 1.000 | 1.000 | 1.000 | 0.627 | 0.692 | 0.660 | 0.856 | 0.909 | 0.883 | 1.417 | 0.971 | 1.194 |
| Kattuponni | 1.000 | 1.000 | 1.000 | 0.807 | 0.878 | 0.843 | 1.152 | 1.219 | 1.186 | 1.052 | 1.013 | 1.032 |
| Mattaikar | 1.000 | 1.000 | 1.000 | 0.939 | 1.106 | 1.022 | 0.847 | 0.971 | 0.909 | 0.795 | 0.868 | 0.832 |
| Norungan | 1.000 | 1.000 | 1.000 | 0.806 | 1.013 | 0.909 | 0.876 | 0.971 | 0.924 | 1.108 | 0.951 | 1.029 |
| Ponmani Samba | 1.000 | 1.000 | 1.000 | 0.932 | 0.940 | 0.936 | 0.961 | 1.023 | 0.992 | 1.089 | 0.992 | 1.041 |
| Poongar | 1.000 | 1.000 | 1.000 | 1.001 | 1.054 | 1.027 | 0.731 | 0.796 | 0.764 | 1.148 | 1.013 | 1.081 |
| Upumolagai | 1.000 | 1.000 | 1.000 | 1.073 | 1.013 | 1.043 | 0.943 | 1.013 | 0.978 | 1.215 | 1.199 | 1.207 |
| Vadakathi Samba | 1.000 | 1.000 | 1.000 | 0.875 | 1.044 | 0.959 | 0.914 | 0.992 | 0.953 | 0.955 | 0.899 | 0.927 |
| Varigarudan Samba | 1.000 | 1.000 | 1.000 | 0.920 | 1.044 | 0.982 | 0.913 | 0.920 | 0.916 | 0.846 | 0.94 | 0.893 |
| **Mean** | **1.000** | **1.000** | **1.000** | **0.892** | **0.959** | **0.926** | **0.987** | **0.998** | **0.997** | **0.991** | **0.977** | **1.005** |
| **LSD** | **4.689** | **0.050** | **1.345** | **0.072** | **0.046** | **0.187** | **0.076** | **0.049** | **0.158** | **0.079** | **0.047** | **0.219** |
| **CV** | **2.990** | **2.957** | **6.661** | **4.994** | **2.973** | **10.020** | **4.748** | **2.979** | **7.860** | **4.682** | **2.964** | **10.780** |
| **SE** | **0.000** | **0.018** | **0.000** | **0.026** | **0.017** | **0.066** | **0.027** | **0.017** | **0.055** | **0.028** | **0.017** | **0.077** |
| **Percent Reduction** | **0.000** | **0.000** | **0.000** | **10.817** | **4.076** | **7.400** | **1.269** | **0.166** | **0.300** | **0.865** | **2.287** | **-0.500** |

| **Traits** | **Relative growth index** | | | | | | | | | | | |
| --- | --- | --- | --- | --- | --- | --- | --- | --- | --- | --- | --- | --- |
|  | **Control** | | | **Drought** | | | **Salinity** | | | **Submergence** | | |
| **Seasons** | **Kharif** | **Rabi** | **Pooled** | **Kharif** | **Rabi** | **Pooled** | **Kharif** | **Rabi** | **Pooled** | **Kharif** | **Rabi** | **Pooled** |
| ADT 45 | 1.000 | 1.000 | 1.000 | 0.153 | 0.382 | 0.267 | 0.947 | 0.486 | 0.717 | 0.079 | 0.083 | 0.081 |
| ADT 51 | 1.000 | 1.000 | 1.000 | 0.705 | 0.610 | 0.657 | 0.682 | 0.517 | 0.599 | 1.864 | 0.186 | 1.025 |
| ADT 52 | 1.000 | 1.000 | 1.000 | 0.150 | 0.145 | 0.147 | 0.667 | 0.579 | 0.623 | 0.108 | 0.103 | 0.106 |
| ADT 53 | 1.000 | 1.000 | 1.000 | 0.867 | 0.589 | 0.728 | 0.400 | 0.393 | 0.396 | 0.080 | 0.083 | 0.081 |
| ADT 54 | 1.000 | 1.000 | 1.000 | 0.186 | 0.186 | 0.186 | 0.364 | 0.403 | 0.383 | 0.145 | 0.145 | 0.145 |
| ADT 56 | 1.000 | 1.000 | 1.000 | 0.070 | 0.072 | 0.071 | 0.767 | 0.465 | 0.616 | 0.400 | 0.269 | 0.334 |
| ADT 57 | 1.000 | 1.000 | 1.000 | 0.124 | 0.124 | 0.124 | 0.414 | 0.413 | 0.414 | 0.552 | 0.238 | 0.395 |
| ANNA R 4 | 1.000 | 1.000 | 1.000 | 0.875 | 0.909 | 0.892 | 0.458 | 0.703 | 0.581 | 0.175 | 0.176 | 0.175 |
| APD19002 | 1.000 | 1.000 | 1.000 | 0.467 | 0.734 | 0.600 | 0.848 | 0.847 | 0.848 | 0.373 | 0.372 | 0.372 |
| Arupatham samba | 1.000 | 1.000 | 1.000 | 0.714 | 0.775 | 0.745 | 0.600 | 0.692 | 0.646 | 0.374 | 0.372 | 0.373 |
| CB 16656 | 1.000 | 1.000 | 1.000 | 0.783 | 0.806 | 0.794 | 0.696 | 0.723 | 0.709 | 0.091 | 0.103 | 0.097 |
| CB 17502 | 1.000 | 1.000 | 1.000 | 0.111 | 0.134 | 0.123 | 0.444 | 0.548 | 0.496 | 0.117 | 0.155 | 0.136 |
| CB 17542 | 1.000 | 1.000 | 1.000 | 0.700 | 0.723 | 0.712 | 0.980 | 0.930 | 0.955 | 0.320 | 0.258 | 0.289 |
| CB 17561 | 1.000 | 1.000 | 1.000 | 1.868 | 0.486 | 1.177 | 0.549 | 0.217 | 0.383 | 0.505 | 0.248 | 0.377 |
| CB 17573 | 1.000 | 1.000 | 1.000 | 1.184 | 0.610 | 0.897 | 0.329 | 0.341 | 0.335 | 0.138 | 0.145 | 0.141 |
| CB 17597 | 1.000 | 1.000 | 1.000 | 1.277 | 0.785 | 1.031 | 0.426 | 0.341 | 0.383 | 0.383 | 0.310 | 0.346 |
| CB 22504 | 1.000 | 1.000 | 1.000 | 1.043 | 0.889 | 0.966 | 0.478 | 0.537 | 0.508 | 0.104 | 0.114 | 0.109 |
| CB 22512 | 1.000 | 1.000 | 1.000 | 0.650 | 0.672 | 0.661 | 0.475 | 0.496 | 0.486 | 0.185 | 0.186 | 0.186 |
| CB 22541 | 1.000 | 1.000 | 1.000 | 1.094 | 0.599 | 0.847 | 0.469 | 0.372 | 0.420 | 0.130 | 0.134 | 0.132 |
| CB 22560 | 1.000 | 1.000 | 1.000 | 0.571 | 0.589 | 0.580 | 0.619 | 0.641 | 0.630 | 0.090 | 0.093 | 0.092 |
| ChittanSamba | 1.000 | 1.000 | 1.000 | 0.909 | 0.579 | 0.744 | 0.480 | 0.496 | 0.488 | 0.131 | 0.134 | 0.133 |
| CO 49 | 1.000 | 1.000 | 1.000 | 0.237 | 0.238 | 0.237 | 0.221 | 0.217 | 0.219 | 0.189 | 0.186 | 0.188 |
| CO 51 | 1.000 | 1.000 | 1.000 | 0.083 | 0.083 | 0.083 | 0.090 | 0.093 | 0.091 | 0.207 | 0.207 | 0.207 |
| CO 52 | 1.000 | 1.000 | 1.000 | 0.057 | 0.093 | 0.075 | 0.810 | 0.620 | 0.715 | 0.429 | 0.207 | 0.318 |
| CO 53 | 1.000 | 1.000 | 1.000 | 0.750 | 0.661 | 0.706 | 0.813 | 0.785 | 0.799 | 0.106 | 0.103 | 0.105 |
| CO 54 | 1.000 | 1.000 | 1.000 | 0.559 | 0.444 | 0.502 | 0.735 | 0.620 | 0.678 | 0.062 | 0.062 | 0.062 |
| CO 55 | 1.000 | 1.000 | 1.000 | 0.762 | 0.692 | 0.727 | 0.667 | 0.589 | 0.628 | 0.171 | 0.176 | 0.174 |
| FL 478 | 1.000 | 1.000 | 1.000 | 0.700 | 0.723 | 0.712 | 0.667 | 0.723 | 0.695 | 0.437 | 0.134 | 0.286 |
| FR 13A | 1.000 | 1.000 | 1.000 | 0.793 | 0.537 | 0.665 | 0.724 | 0.641 | 0.682 | 0.655 | 0.537 | 0.596 |
| IR 42 | 1.000 | 1.000 | 1.000 | 0.899 | 0.465 | 0.682 | 0.373 | 0.568 | 0.471 | 0.140 | 0.134 | 0.137 |
| IR 64 | 1.000 | 1.000 | 1.000 | 1.152 | 0.434 | 0.793 | 0.788 | 0.496 | 0.642 | 0.152 | 0.155 | 0.153 |
| IR 64 DRT | 1.000 | 1.000 | 1.000 | 0.709 | 0.734 | 0.722 | 0.709 | 0.661 | 0.685 | 0.101 | 0.103 | 0.102 |
| Kappikar | 1.000 | 1.000 | 1.000 | 0.594 | 0.610 | 0.602 | 0.813 | 0.517 | 0.665 | 0.563 | 0.258 | 0.410 |
| Kattuponni | 1.000 | 1.000 | 1.000 | 0.935 | 0.548 | 0.742 | 0.516 | 0.548 | 0.532 | 0.090 | 0.093 | 0.092 |
| Mattaikar | 1.000 | 1.000 | 1.000 | 0.886 | 0.889 | 0.887 | 0.743 | 0.837 | 0.790 | 0.657 | 0.403 | 0.530 |
| Norungan | 1.000 | 1.000 | 1.000 | 0.943 | 0.971 | 0.957 | 0.771 | 0.734 | 0.753 | 0.231 | 0.238 | 0.235 |
| Ponmani Samba | 1.000 | 1.000 | 1.000 | 0.962 | 0.703 | 0.832 | 0.692 | 0.537 | 0.615 | 0.138 | 0.145 | 0.142 |
| Poongar | 1.000 | 1.000 | 1.000 | 0.658 | 0.734 | 0.696 | 0.605 | 0.682 | 0.644 | 0.358 | 0.269 | 0.313 |
| Upumolagai | 1.000 | 1.000 | 1.000 | 0.808 | 0.558 | 0.683 | 0.808 | 0.434 | 0.621 | 0.081 | 0.083 | 0.082 |
| Vadakathi Samba | 1.000 | 1.000 | 1.000 | 0.769 | 0.837 | 0.803 | 0.423 | 0.496 | 0.460 | 0.119 | 0.537 | 0.328 |
| Varigarudan Samba | 1.000 | 1.000 | 1.000 | 0.857 | 0.775 | 0.816 | 0.821 | 0.744 | 0.783 | 0.329 | 0.486 | 0.407 |
| **Mean** | **1.000** | **1.000** | **1.000** | **0.698** | **0.564** | **0.631** | **0.607** | **0.553** | **0.580** | **0.282** | **0.205** | **0.244** |
| **LSD** | **4.689** | **0.050** | **1.345** | **0.065** | **0.030** | **0.456** | **0.064** | **0.028** | **0.226** | **0.030** | **0.011** | **0.419** |
| **CV** | **2.997** | **2.957** | **6.661** | **5.694** | **3.233** | **35.751** | **6.449** | **3.101** | **19.287** | **6.480** | **3.433** | **85.102** |
| **SE** | **0.000** | **0.018** | **0.000** | **0.022** | **0.011** | **0.160** | **0.023** | **0.010** | **0.079** | **0.011** | **0.004** | **0.147** |
| **Percent Reduction** | **0.000** | **0.000** | **0.000** | **30.213** | **43.595** | **36.900** | **39.316** | **44.679** | **42.000** | **71.801** | **79.459** | **75.600** |
