# Supplementary material for: Enhancing stress resilience in rice (Oryza sativa L.) through profiling early-stage morpho-physiological and molecular responses to multiple abiotic stress tolerance
Source: Front Plant Sci. 2024 Feb 8;15:1342441. doi: 10.3389/fpls.2024.1342441 (PMC10882102; doi:10.3389/fpls.2024.1342441)
Supplement: Supplementary file 3 [file Table_3.docx]

Supplementary table 3. Stress tolerance index for drought, salinity and submergence of rice genotypes

| **Indices** | **Drought tolerance index (DTI)** | | | **Salinity tolerance index (STI)** | | | **Submergence tolerance index (FTI)** | | |
| --- | --- | --- | --- | --- | --- | --- | --- | --- | --- |
| **Genotype Name** | **Kharif** | **Rabi** | **Pooled** | **Kharif** | **Rabi** | **Pooled** | **Kharif** | **Rabi** | **Pooled** |
| **CO 51** | 0.805 | 0.72 | 0.760 | 0.81 | 0.11 | 0.46 | 0.95 | 0.71 | 0.83 |
| **CO 52** | 0.909 | 1.08 | 0.994 | 0.96 | 1.16 | 1.06 | 0.88 | 0.98 | 0.93 |
| **CO 53** | 1.041 | 1.10 | 1.071 | 1.07 | 1.31 | 1.19 | 1.01 | 0.20 | 0.60 |
| **CO 54** | 0.974 | 1.12 | 1.046 | 1.00 | 0.77 | 0.88 | 1.02 | 0.52 | 0.77 |
| **CO 55** | 0.999 | 1.14 | 1.068 | 0.99 | 0.76 | 0.87 | 1.00 | 0.90 | 0.95 |
| **ADT 51** | 1.024 | 0.84 | 0.930 | 0.95 | 0.98 | 0.97 | 0.98 | 0.96 | 0.97 |
| **ADT 52** | 0.814 | 0.15 | 0.483 | 0.97 | 1.13 | 1.05 | 0.98 | 1.13 | 1.06 |
| **ADT 54** | 0.843 | 1.20 | 1.019 | 0.90 | 1.41 | 1.16 | 0.86 | 0.29 | 0.58 |
| **ADT 56** | 0.776 | 0.97 | 0.873 | 0.94 | 1.05 | 1.00 | 1.02 | 0.45 | 0.73 |
| **ADT 57** | 0.814 | 1.22 | 1.015 | 0.92 | 1.16 | 1.04 | 0.90 | 1.14 | 1.02 |
| **ADT 45** | 0.903 | 1.16 | 1.032 | 0.95 | 1.31 | 1.13 | 1.02 | 0.16 | 0.59 |
| **APD19002** | 1.045 | 1.09 | 1.067 | 1.09 | 1.00 | 1.05 | 1.17 | 1.68 | 1.42 |
| **ADT 53** | 1.003 | 0.90 | 0.952 | 0.92 | 1.03 | 0.97 | 0.82 | 0.13 | 0.48 |
| **CO 49** | 0.848 | 1.20 | 1.024 | 0.84 | 0.27 | 0.56 | 0.87 | 0.20 | 0.53 |
| **ANNA R 4** | 1.096 | 1.15 | 1.121 | 0.94 | 1.33 | 1.14 | 1.25 | 0.35 | 0.80 |
| **CB 17502** | 0.802 | 1.12 | 0.960 | 0.92 | 0.54 | 0.73 | 1.21 | 0.96 | 1.09 |
| **CB 17542** | 1.027 | 0.39 | 0.710 | 1.07 | 0.63 | 0.85 | 0.91 | 0.92 | 0.91 |
| **CB 17561** | 0.837 | 0.54 | 0.687 | 0.71 | 0.58 | 0.64 | 0.73 | 0.87 | 0.80 |
| **CB 17573** | 0.983 | 1.18 | 1.081 | 0.88 | 0.41 | 0.65 | 0.85 | 1.06 | 0.95 |
| **CB 17597** | 0.971 | 1.00 | 0.984 | 0.80 | 0.48 | 0.64 | 0.81 | 1.54 | 1.18 |
| **CB 22504** | 1.071 | 0.97 | 1.020 | 0.95 | 0.96 | 0.96 | 0.84 | 0.96 | 0.90 |
| **CB 22512** | 1.015 | 0.74 | 0.875 | 0.95 | 1.17 | 1.06 | 1.00 | 0.91 | 0.95 |
| **CB 22541** | 0.972 | 0.68 | 0.825 | 0.94 | 1.21 | 1.08 | 0.84 | 1.16 | 1.00 |
| **CB 22560** | 0.979 | 0.78 | 0.882 | 0.99 | 0.85 | 0.92 | 0.83 | 0.92 | 0.88 |
| **CB 16656** | 1.056 | 0.86 | 0.960 | 1.01 | 0.64 | 0.83 | 0.83 | 0.95 | 0.89 |
| **Arupatham samba** | 1.060 | 1.01 | 1.035 | 1.03 | 1.35 | 1.19 | 1.09 | 1.63 | 1.36 |
| **Poongar** | 1.005 | 1.09 | 1.046 | 1.00 | 0.71 | 0.85 | 1.07 | 0.41 | 0.74 |
| **Kappikar** | 0.980 | 0.68 | 0.832 | 0.96 | 1.34 | 1.15 | 0.89 | 0.94 | 0.91 |
| **Varigarudan Samba** | 1.083 | 1.12 | 1.104 | 1.07 | 1.02 | 1.04 | 1.16 | 1.42 | 1.29 |
| **Upumolagai** | 0.975 | 0.61 | 0.792 | 0.93 | 0.52 | 0.72 | 1.06 | 1.08 | 1.07 |
| **ChittanSamba** | 0.966 | 1.05 | 1.010 | 0.95 | 1.19 | 1.07 | 1.03 | 0.80 | 0.91 |
| **Norungan** | 1.119 | 1.13 | 1.123 | 1.02 | 1.34 | 1.18 | 0.93 | 1.09 | 1.01 |
| **Ponmani Samba** | 1.022 | 1.06 | 1.039 | 0.95 | 1.26 | 1.10 | 0.86 | 0.84 | 0.85 |
| **Vadakathi Samba** | 1.048 | 0.78 | 0.913 | 0.94 | 1.24 | 1.09 | 0.85 | 1.29 | 1.07 |
| **Kattuponni** | 0.969 | 0.62 | 0.795 | 0.97 | 0.68 | 0.82 | 0.84 | 1.04 | 0.94 |
| **FL 478** | 1.034 | 1.11 | 1.074 | 1.02 | 1.26 | 1.14 | 0.98 | 0.98 | 0.98 |
| **FR 13A** | 0.962 | 1.14 | 1.049 | 1.00 | 1.13 | 1.06 | 1.44 | 1.80 | 1.62 |
| **IR 64 DRT** | 1.023 | 1.02 | 1.024 | 1.01 | 0.79 | 0.90 | 0.83 | 1.49 | 1.16 |
| **IR 64** | 0.911 | 0.97 | 0.940 | 0.93 | 1.02 | 0.97 | 0.85 | 0.84 | 0.84 |
| **Mattaikar** | 1.089 | 1.02 | 1.052 | 1.07 | 1.19 | 1.13 | 1.18 | 1.74 | 1.46 |
| **IR 42** | 0.941 | 1.06 | 0.999 | 0.97 | 0.66 | 0.82 | 0.85 | 0.87 | 0.86 |
