# Supplementary material for: Enhancing stress resilience in rice (Oryza sativa L.) through profiling early-stage morpho-physiological and molecular responses to multiple abiotic stress tolerance
Source: Front Plant Sci. 2024 Feb 8;15:1342441. doi: 10.3389/fpls.2024.1342441 (PMC10882102; doi:10.3389/fpls.2024.1342441)
Supplement: Supplementary file 4 [file Table_4.docx]

Supplementary Table 4. Gas exchange parameters

| **Traits** | | **Transpiration rate** | | | | | | | | | | | | | | | | | | | | | | | |
| --- | --- | --- | --- | --- | --- | --- | --- | --- | --- | --- | --- | --- | --- | --- | --- | --- | --- | --- | --- | --- | --- | --- | --- | --- | --- |
|  |  | **Control** | | | | | | **Drought** | | | | | | **Salinity** | | | | | | **Submergence** | | | | | |
| **Seasons** | | **Kharif** | | **Rabi** | | **Pooled** | | **Kharif** | | **Rabi** | | **Pooled** | | **Kharif** | | **Rabi** | | **Pooled** | | **Kharif** | | **Rabi** | | **Pooled** | |
| ADT 45 | | 1.400 | | 1.470 | | 1.435 | | 0.030 | | 0.031 | | 0.031 | | 0.580 | | 0.543 | | 0.562 | | 0.226 | | 0.232 | | 0.229 | |
| ADT 51 | | 1.210 | | 1.236 | | 1.223 | | 0.296 | | 0.299 | | 0.297 | | 1.410 | | 1.493 | | 1.451 | | 0.260 | | 0.269 | | 0.265 | |
| ADT 52 | | 1.397 | | 1.426 | | 1.411 | | 0.792 | | 0.829 | | 0.811 | | 1.340 | | 1.387 | | 1.364 | | 0.476 | | 0.464 | | 0.470 | |
| ADT 53 | | 1.340 | | 1.362 | | 1.351 | | 0.494 | | 0.523 | | 0.509 | | 0.760 | | 0.794 | | 0.777 | | 0.036 | | 0.036 | | 0.036 | |
| ADT 54 | | 1.420 | | 1.465 | | 1.443 | | 0.766 | | 0.853 | | 0.810 | | 1.750 | | 1.790 | | 1.770 | | 0.174 | | 0.189 | | 0.182 | |
| ADT 56 | | 1.853 | | 1.874 | | 1.864 | | 0.378 | | 0.389 | | 0.384 | | 1.855 | | 1.829 | | 1.842 | | 0.522 | | 0.547 | | 0.535 | |
| ADT 57 | | 1.177 | | 1.244 | | 1.210 | | 0.096 | | 0.097 | | 0.097 | | 1.670 | | 1.747 | | 1.708 | | 0.414 | | 0.438 | | 0.426 | |
| ANNA R 4 | | 2.830 | | 2.988 | | 2.909 | | 0.250 | | 0.260 | | 0.255 | | 2.960 | | 3.113 | | 3.036 | | 0.172 | | 0.172 | | 0.172 | |
| APD19002 | | 1.540 | | 1.554 | | 1.547 | | 0.980 | | 1.081 | | 1.030 | | 0.620 | | 0.636 | | 0.628 | | 1.620 | | 1.647 | | 1.633 | |
| Arupatham samba | | 1.920 | | 1.938 | | 1.929 | | 1.304 | | 1.343 | | 1.324 | | 1.330 | | 1.466 | | 1.398 | | 1.352 | | 1.427 | | 1.389 | |
| CB 16656 | | 1.900 | | 1.844 | | 1.872 | | 0.954 | | 0.987 | | 0.971 | | 0.870 | | 0.820 | | 0.845 | | 0.054 | | 0.053 | | 0.053 | |
| CB 17502 | | 2.963 | | 2.936 | | 2.950 | | 0.534 | | 0.588 | | 0.561 | | 2.890 | | 2.817 | | 2.854 | | 0.594 | | 0.630 | | 0.612 | |
| CB 17542 | | 2.857 | | 3.138 | | 2.997 | | 0.180 | | 0.190 | | 0.185 | | 2.740 | | 2.737 | | 2.738 | | 0.330 | | 0.334 | | 0.332 | |
| CB 17561 | | 2.647 | | 2.744 | | 2.695 | | 0.112 | | 0.115 | | 0.114 | | 2.560 | | 2.682 | | 2.621 | | 0.258 | | 0.273 | | 0.265 | |
| CB 17573 | | 2.467 | | 2.512 | | 2.489 | | 0.036 | | 0.034 | | 0.035 | | 2.415 | | 2.489 | | 2.452 | | 0.220 | | 0.221 | | 0.221 | |
| CB 17597 | | 1.127 | | 1.241 | | 1.184 | | 0.328 | | 0.358 | | 0.343 | | 0.060 | | 0.062 | | 0.061 | | 0.084 | | 0.093 | | 0.088 | |
| CB 22504 | | 1.873 | | 1.814 | | 1.844 | | 0.540 | | 0.598 | | 0.569 | | 1.595 | | 1.686 | | 1.641 | | 0.450 | | 0.446 | | 0.448 | |
| CB 22512 | | 1.310 | | 1.266 | | 1.288 | | 0.636 | | 0.669 | | 0.653 | | 0.150 | | 0.164 | | 0.157 | | 0.048 | | 0.050 | | 0.049 | |
| CB 22541 | | 1.917 | | 1.955 | | 1.936 | | 0.434 | | 0.452 | | 0.443 | | 2.385 | | 2.492 | | 2.439 | | 0.026 | | 0.028 | | 0.027 | |
| CB 22560 | | 1.530 | | 1.593 | | 1.562 | | 0.842 | | 0.914 | | 0.878 | | 0.895 | | 0.895 | | 0.895 | | 0.062 | | 0.063 | | 0.062 | |
| ChittanSamba | | 1.513 | | 1.599 | | 1.556 | | 0.232 | | 0.251 | | 0.242 | | 0.982 | | 0.942 | | 0.962 | | 1.240 | | 1.261 | | 1.251 | |
| CO 49 | | 1.300 | | 1.316 | | 1.308 | | 0.124 | | 0.128 | | 0.126 | | 0.615 | | 0.631 | | 0.623 | | 0.032 | | 0.033 | | 0.032 | |
| CO 51 | | 1.483 | | 1.448 | | 1.466 | | 0.572 | | 0.644 | | 0.608 | | 1.140 | | 1.064 | | 1.102 | | 0.020 | | 0.020 | | 0.020 | |
| CO 52 | | 1.140 | | 1.142 | | 1.141 | | 0.438 | | 0.425 | | 0.431 | | 1.100 | | 1.137 | | 1.119 | | 0.220 | | 0.215 | | 0.217 | |
| CO 53 | | 3.010 | | 3.171 | | 3.091 | | 0.620 | | 0.620 | | 0.620 | | 2.950 | | 3.147 | | 3.049 | | 0.048 | | 0.052 | | 0.050 | |
| CO 54 | | 2.907 | | 3.115 | | 3.011 | | 0.342 | | 0.348 | | 0.345 | | 2.900 | | 2.928 | | 2.914 | | 0.422 | | 0.436 | | 0.429 | |
| CO 55 | | 1.440 | | 1.461 | | 1.450 | | 0.284 | | 0.293 | | 0.288 | | 1.490 | | 1.664 | | 1.577 | | 0.484 | | 0.453 | | 0.468 | |
| FL 478 | | 1.420 | | 1.333 | | 1.376 | | 1.392 | | 1.566 | | 1.479 | | 2.970 | | 3.045 | | 3.007 | | 0.230 | | 0.237 | | 0.234 | |
| FR 13A | | 3.097 | | 3.416 | | 3.256 | | 1.712 | | 1.825 | | 1.768 | | 1.210 | | 1.256 | | 1.233 | | 1.492 | | 1.545 | | 1.518 | |
| IR 42 | | 1.529 | | 1.615 | | 1.572 | | 0.371 | | 0.238 | | 0.304 | | 0.840 | | 0.568 | | 0.704 | | 0.163 | | 0.238 | | 0.201 | |
| IR 64 | | 1.421 | | 1.409 | | 1.415 | | 0.596 | | 0.612 | | 0.604 | | 1.545 | | 1.746 | | 1.645 | | 0.380 | | 0.392 | | 0.386 | |
| IR 64 DRT | | 1.527 | | 1.516 | | 1.521 | | 0.568 | | 0.540 | | 0.554 | | 1.060 | | 1.100 | | 1.080 | | 0.226 | | 0.232 | | 0.229 | |
| Kappikar | | 2.203 | | 2.295 | | 2.249 | | 0.694 | | 0.728 | | 0.711 | | 2.710 | | 2.948 | | 2.829 | | 0.484 | | 0.489 | | 0.486 | |
| Kattuponni | | 1.103 | | 1.162 | | 1.132 | | 1.278 | | 1.345 | | 1.312 | | 3.100 | | 3.060 | | 3.080 | | 0.114 | | 0.123 | | 0.118 | |
| Mattaikar | | 1.967 | | 1.965 | | 1.966 | | 1.484 | | 1.524 | | 1.504 | | 1.615 | | 1.561 | | 1.588 | | 1.122 | | 1.151 | | 1.136 | |
| Norungan | | 2.640 | | 2.748 | | 2.694 | | 1.532 | | 1.595 | | 1.564 | | 2.795 | | 2.948 | | 2.871 | | 1.302 | | 1.215 | | 1.258 | |
| Ponmani Samba | | 1.040 | | 1.114 | | 1.077 | | 1.924 | | 2.169 | | 2.047 | | 0.810 | | 0.855 | | 0.833 | | 0.086 | | 0.088 | | 0.087 | |
| Poongar | | 1.340 | | 1.388 | | 1.364 | | 0.772 | | 0.810 | | 0.791 | | 2.810 | | 2.974 | | 2.892 | | 0.360 | | 0.401 | | 0.380 | |
| Upumolagai | | 1.273 | | 1.287 | | 1.280 | | 0.584 | | 0.615 | | 0.599 | | 2.565 | | 2.684 | | 2.625 | | 0.490 | | 0.460 | | 0.475 | |
| Vadakathi Samba | | 1.129 | | 1.078 | | 1.103 | | 0.562 | | 0.550 | | 0.556 | | 0.860 | | 0.874 | | 0.867 | | 0.052 | | 0.054 | | 0.053 | |
| Varigarudan Samba | | 2.160 | | 2.183 | | 2.171 | | 0.046 | | 0.047 | | 0.046 | | 2.270 | | 2.519 | | 2.394 | | 1.594 | | 1.654 | | 1.624 | |
| **Mean** | | **1.788** | | **1.838** | | **1.813** | | **0.637** | | **0.670** | | **0.654** | | **1.687** | | **1.739** | | **1.713** | | **0.438** | | **0.448** | | **0.443** | |
| **LSD** | | **0.149** | | **0.094** | | **0.139** | | **0.078** | | **0.041** | | **0.094** | | **0.173** | | **0.095** | | **0.158** | | **0.052** | | **0.031** | | **0.041** | |
| **CV** | | **5.119** | | **3.148** | | **3.794** | | **7.559** | | **3.731** | | **7.139** | | **6.325** | | **3.356** | | **4.568** | | **7.312** | | **4.303** | | **4.633** | |
| **SE** | | **0.052** | | **0.033** | | **0.049** | | **0.028** | | **0.014** | | **0.033** | | **0.062** | | **0.034** | | **0.055** | | **0.019** | | **0.011** | | **0.015** | |
| **Percent Reduction** | | **0.000** | | **0.000** | | **0.000** | | **64.390** | | **63.526** | | **63.927** | | **5.656** | | **5.393** | | **5.516** | | **75.533** | | **75.640** | | **75.565** | |
| **Traits** | **Stomatal conducatnce** | | | | | | | | | | | | | | | | | | | | | | | |  |
|  | **Control** | | | | | | **Drought** | | | | | | **Salinity** | | | | | | **Submergence** | | | | | |  |
| **Seasons** | **Kharif** | | **Rabi** | | **Pooled** | | **Kharif** | | **Rabi** | | **Pooled** | | **Kharif** | | **Rabi** | | **Pooled** | | **Kharif** | | **Rabi** | | **Pooled** | |  |
| ADT 45 | 0.010 | | 0.010 | | 0.010 | | 0.010 | | 0.010 | | 0.010 | | 0.010 | | 0.009 | | 0.010 | | 0.003 | | 0.003 | | 0.003 | |  |
| ADT 51 | 0.020 | | 0.020 | | 0.020 | | 0.013 | | 0.014 | | 0.013 | | 0.010 | | 0.010 | | 0.010 | | 0.001 | | 0.001 | | 0.001 | |  |
| ADT 52 | 0.020 | | 0.020 | | 0.020 | | 0.020 | | 0.020 | | 0.020 | | 0.020 | | 0.021 | | 0.020 | | 0.002 | | 0.002 | | 0.002 | |  |
| ADT 53 | 0.010 | | 0.010 | | 0.010 | | 0.010 | | 0.010 | | 0.010 | | 0.010 | | 0.009 | | 0.010 | | 0.002 | | 0.002 | | 0.002 | |  |
| ADT 54 | 0.030 | | 0.028 | | 0.029 | | 0.027 | | 0.025 | | 0.026 | | 0.026 | | 0.026 | | 0.026 | | 0.001 | | 0.001 | | 0.001 | |  |
| ADT 56 | 0.030 | | 0.031 | | 0.030 | | 0.030 | | 0.032 | | 0.031 | | 0.010 | | 0.010 | | 0.010 | | 0.001 | | 0.001 | | 0.001 | |  |
| ADT 57 | 0.030 | | 0.032 | | 0.031 | | 0.023 | | 0.024 | | 0.023 | | 0.010 | | 0.011 | | 0.011 | | 0.001 | | 0.001 | | 0.001 | |  |
| ANNA R 4 | 0.050 | | 0.052 | | 0.051 | | 0.050 | | 0.050 | | 0.050 | | 0.040 | | 0.042 | | 0.041 | | 0.002 | | 0.002 | | 0.002 | |  |
| APD19002 | 0.010 | | 0.010 | | 0.010 | | 0.010 | | 0.010 | | 0.010 | | 0.060 | | 0.061 | | 0.060 | | 0.020 | | 0.020 | | 0.020 | |  |
| Arupatham samba | 0.020 | | 0.021 | | 0.020 | | 0.003 | | 0.003 | | 0.003 | | 0.070 | | 0.070 | | 0.070 | | 0.003 | | 0.003 | | 0.003 | |  |
| CB 16656 | 0.010 | | 0.011 | | 0.010 | | 0.010 | | 0.010 | | 0.010 | | 0.020 | | 0.019 | | 0.019 | | 0.005 | | 0.005 | | 0.005 | |  |
| CB 17502 | 0.050 | | 0.048 | | 0.049 | | 0.050 | | 0.052 | | 0.051 | | 0.010 | | 0.010 | | 0.010 | | 0.010 | | 0.010 | | 0.010 | |  |
| CB 17542 | 0.050 | | 0.050 | | 0.050 | | 0.050 | | 0.048 | | 0.049 | | 0.010 | | 0.010 | | 0.010 | | 0.006 | | 0.006 | | 0.006 | |  |
| CB 17561 | 0.040 | | 0.041 | | 0.041 | | 0.047 | | 0.044 | | 0.045 | | 0.010 | | 0.011 | | 0.010 | | 0.004 | | 0.004 | | 0.004 | |  |
| CB 17573 | 0.040 | | 0.042 | | 0.041 | | 0.040 | | 0.043 | | 0.041 | | 0.010 | | 0.010 | | 0.010 | | 0.002 | | 0.002 | | 0.002 | |  |
| CB 17597 | 0.030 | | 0.031 | | 0.031 | | 0.010 | | 0.010 | | 0.010 | | 0.010 | | 0.010 | | 0.010 | | 0.003 | | 0.003 | | 0.003 | |  |
| CB 22504 | 0.025 | | 0.025 | | 0.025 | | 0.030 | | 0.032 | | 0.031 | | 0.010 | | 0.010 | | 0.010 | | 0.002 | | 0.002 | | 0.002 | |  |
| CB 22512 | 0.020 | | 0.021 | | 0.020 | | 0.020 | | 0.020 | | 0.020 | | 0.010 | | 0.010 | | 0.010 | | 0.004 | | 0.004 | | 0.004 | |  |
| CB 22541 | 0.040 | | 0.042 | | 0.041 | | 0.033 | | 0.037 | | 0.035 | | 0.012 | | 0.013 | | 0.012 | | 0.003 | | 0.003 | | 0.003 | |  |
| CB 22560 | 0.010 | | 0.011 | | 0.010 | | 0.010 | | 0.010 | | 0.010 | | 0.016 | | 0.016 | | 0.016 | | 0.003 | | 0.003 | | 0.003 | |  |
| ChittanSamba | 0.010 | | 0.010 | | 0.010 | | 0.007 | | 0.007 | | 0.007 | | 0.010 | | 0.010 | | 0.010 | | 0.002 | | 0.002 | | 0.002 | |  |
| CO 49 | 0.010 | | 0.011 | | 0.011 | | 0.010 | | 0.010 | | 0.010 | | 0.010 | | 0.010 | | 0.010 | | 0.002 | | 0.002 | | 0.002 | |  |
| CO 51 | 0.020 | | 0.021 | | 0.020 | | 0.027 | | 0.025 | | 0.026 | | 0.020 | | 0.021 | | 0.020 | | 0.003 | | 0.003 | | 0.003 | |  |
| CO 52 | 0.020 | | 0.021 | | 0.020 | | 0.020 | | 0.021 | | 0.020 | | 0.010 | | 0.010 | | 0.010 | | 0.002 | | 0.002 | | 0.002 | |  |
| CO 53 | 0.050 | | 0.055 | | 0.053 | | 0.053 | | 0.050 | | 0.052 | | 0.063 | | 0.065 | | 0.064 | | 0.070 | | 0.074 | | 0.072 | |  |
| CO 54 | 0.050 | | 0.051 | | 0.051 | | 0.050 | | 0.052 | | 0.051 | | 0.008 | | 0.008 | | 0.008 | | 0.002 | | 0.002 | | 0.002 | |  |
| CO 55 | 0.030 | | 0.031 | | 0.031 | | 0.020 | | 0.020 | | 0.020 | | 0.010 | | 0.010 | | 0.010 | | 0.003 | | 0.003 | | 0.003 | |  |
| FL 478 | 0.010 | | 0.010 | | 0.010 | | 0.010 | | 0.011 | | 0.011 | | 0.022 | | 0.023 | | 0.022 | | 0.020 | | 0.020 | | 0.020 | |  |
| FR 13A | 0.050 | | 0.054 | | 0.052 | | 0.050 | | 0.053 | | 0.052 | | 0.030 | | 0.034 | | 0.032 | | 0.062 | | 0.058 | | 0.060 | |  |
| IR 42 | 0.017 | | 0.020 | | 0.018 | | 0.002 | | 0.003 | | 0.002 | | 0.008 | | 0.008 | | 0.008 | | 0.011 | | 0.012 | | 0.012 | |  |
| IR 64 | 0.025 | | 0.026 | | 0.026 | | 0.020 | | 0.022 | | 0.021 | | 0.010 | | 0.011 | | 0.010 | | 0.020 | | 0.021 | | 0.021 | |  |
| IR 64 DRT | 0.020 | | 0.020 | | 0.020 | | 0.003 | | 0.003 | | 0.003 | | 0.008 | | 0.008 | | 0.008 | | 0.030 | | 0.031 | | 0.031 | |  |
| Kappikar | 0.040 | | 0.044 | | 0.042 | | 0.033 | | 0.035 | | 0.034 | | 0.010 | | 0.010 | | 0.010 | | 0.001 | | 0.001 | | 0.001 | |  |
| Kattuponni | 0.050 | | 0.050 | | 0.050 | | 0.013 | | 0.014 | | 0.014 | | 0.024 | | 0.027 | | 0.025 | | 0.035 | | 0.033 | | 0.034 | |  |
| Mattaikar | 0.040 | | 0.041 | | 0.041 | | 0.033 | | 0.037 | | 0.035 | | 0.064 | | 0.066 | | 0.065 | | 0.040 | | 0.042 | | 0.041 | |  |
| Norungan | 0.040 | | 0.039 | | 0.039 | | 0.040 | | 0.042 | | 0.041 | | 0.026 | | 0.026 | | 0.026 | | 0.031 | | 0.031 | | 0.031 | |  |
| Ponmani Samba | 0.010 | | 0.010 | | 0.010 | | 0.013 | | 0.014 | | 0.014 | | 0.040 | | 0.042 | | 0.041 | | 0.017 | | 0.019 | | 0.018 | |  |
| Poongar | 0.040 | | 0.041 | | 0.041 | | 0.030 | | 0.031 | | 0.030 | | 0.016 | | 0.017 | | 0.017 | | 0.003 | | 0.003 | | 0.003 | |  |
| Upumolagai | 0.040 | | 0.042 | | 0.041 | | 0.017 | | 0.017 | | 0.017 | | 0.010 | | 0.010 | | 0.010 | | 0.001 | | 0.001 | | 0.001 | |  |
| Vadakathi Samba | 0.010 | | 0.011 | | 0.010 | | 0.010 | | 0.011 | | 0.010 | | 0.018 | | 0.017 | | 0.018 | | 0.008 | | 0.006 | | 0.007 | |  |
| Varigarudan Samba | 0.035 | | 0.034 | | 0.035 | | 0.033 | | 0.035 | | 0.034 | | 0.010 | | 0.010 | | 0.010 | | 0.001 | | 0.001 | | 0.001 | |  |
| **Mean** | **0.028** | | **0.029** | | **0.029** | | **0.024** | | **0.025** | | **0.024** | | **0.020** | | **0.020** | | **0.020** | | **0.011** | | **0.011** | | **0.011** | |  |
| **LSD** | **0.003** | | **0.002** | | **0.002** | | **0.003** | | **0.001** | | **0.002** | | **0.002** | | **0.001** | | **0.002** | | **0.005** | | **0.001** | | **0.002** | |  |
| **CV** | **6.926** | | **3.314** | | **4.041** | | **6.410** | | **3.484** | | **4.586** | | **6.160** | | **3.875** | | **3.999** | | **25.920** | | **5.305** | | **7.478** | |  |
| **SE** | **0.001** | | **0.006** | | **0.001** | | **0.001** | | **0.005** | | **0.001** | | **0.001** | | **0.005** | | **0.001** | | **0.002** | | **0.003** | | **0.001** | |  |
| **Percent Reduction** | **0.000** | | **0.000** | | **0.000** | | **14.683** | | **15.332** | | **17.241** | | **30.255** | | **30.800** | | **31.034** | | **62.013** | | **62.660** | | **62.069** | |  |

| **Traits** | **Photosynthetic rate** | | | | | | | | | | | |
| --- | --- | --- | --- | --- | --- | --- | --- | --- | --- | --- | --- | --- |
|  | **Control** | | | **Drought** | | | **Salinity** | | | **Submergence** | | |
| **Seasons** | **Kharif** | **Rabi** | **Pooled** | **Kharif** | **Rabi** | **Pooled** | **Kharif** | **Rabi** | **Pooled** | **Kharif** | **Rabi** | **Pooled** |
| ADT 45 | 1.178 | 1.224 | 1.201 | 0.477 | 0.489 | 0.483 | 0.515 | 0.526 | 0.520 | 0.428 | 0.434 | 0.431 |
| ADT 51 | 1.844 | 1.886 | 1.865 | 0.257 | 0.263 | 0.260 | 0.550 | 0.555 | 0.552 | 0.428 | 0.421 | 0.425 |
| ADT 52 | 2.136 | 2.089 | 2.112 | 0.750 | 0.781 | 0.766 | 0.700 | 0.662 | 0.681 | 0.604 | 0.597 | 0.601 |
| ADT 53 | 1.412 | 1.469 | 1.440 | 0.887 | 0.904 | 0.895 | 1.515 | 1.551 | 1.533 | 0.068 | 0.071 | 0.070 |
| ADT 54 | 2.556 | 2.738 | 2.647 | 1.553 | 1.636 | 1.595 | 1.920 | 1.885 | 1.902 | 2.116 | 2.156 | 2.136 |
| ADT 56 | 1.776 | 1.868 | 1.822 | 1.647 | 1.703 | 1.675 | 1.415 | 1.395 | 1.405 | 0.700 | 0.713 | 0.706 |
| ADT 57 | 3.540 | 3.631 | 3.585 | 0.790 | 0.807 | 0.799 | 0.860 | 0.871 | 0.866 | 2.290 | 2.505 | 2.397 |
| ANNA R 4 | 1.925 | 1.944 | 1.934 | 1.223 | 1.286 | 1.255 | 1.200 | 1.222 | 1.211 | 0.336 | 0.370 | 0.353 |
| APD19002 | 1.686 | 1.835 | 1.761 | 1.507 | 1.642 | 1.574 | 1.500 | 1.471 | 1.485 | 1.644 | 1.686 | 1.665 |
| Arupatham samba | 1.220 | 1.315 | 1.267 | 2.533 | 2.722 | 2.628 | 1.950 | 1.950 | 1.950 | 1.772 | 1.796 | 1.784 |
| CB 16656 | 1.244 | 1.265 | 1.254 | 0.487 | 0.550 | 0.519 | 0.300 | 0.311 | 0.306 | 2.250 | 2.381 | 2.315 |
| CB 17502 | 1.348 | 1.352 | 1.350 | 2.253 | 2.402 | 2.328 | 1.700 | 1.759 | 1.729 | 1.418 | 1.494 | 1.456 |
| CB 17542 | 3.564 | 3.744 | 3.654 | 1.710 | 1.740 | 1.725 | 2.240 | 2.177 | 2.208 | 0.956 | 0.911 | 0.934 |
| CB 17561 | 1.212 | 1.130 | 1.171 | 2.090 | 2.128 | 2.109 | 2.220 | 2.246 | 2.233 | 0.904 | 1.025 | 0.964 |
| CB 17573 | 3.400 | 3.554 | 3.477 | 1.890 | 2.103 | 1.996 | 1.855 | 1.935 | 1.895 | 0.744 | 0.693 | 0.718 |
| CB 17597 | 2.840 | 3.086 | 2.963 | 0.387 | 0.425 | 0.406 | 0.520 | 0.531 | 0.525 | 1.108 | 1.135 | 1.122 |
| CB 22504 | 1.680 | 1.734 | 1.707 | 1.857 | 1.913 | 1.885 | 1.630 | 1.648 | 1.639 | 0.840 | 0.856 | 0.848 |
| CB 22512 | 1.302 | 1.340 | 1.321 | 0.937 | 0.959 | 0.948 | 0.690 | 0.708 | 0.699 | 0.804 | 0.797 | 0.800 |
| CB 22541 | 3.842 | 4.002 | 3.922 | 3.143 | 3.190 | 3.167 | 2.860 | 2.686 | 2.773 | 0.768 | 0.835 | 0.801 |
| CB 22560 | 1.596 | 1.643 | 1.620 | 1.627 | 1.709 | 1.668 | 0.955 | 0.976 | 0.966 | 0.836 | 0.900 | 0.868 |
| ChittanSamba | 1.852 | 1.788 | 1.820 | 0.973 | 0.995 | 0.984 | 0.675 | 0.672 | 0.673 | 0.862 | 0.893 | 0.878 |
| CO 49 | 2.582 | 2.744 | 2.663 | 0.607 | 0.637 | 0.622 | 0.600 | 0.659 | 0.629 | 0.002 | 0.002 | 0.002 |
| CO 51 | 2.096 | 2.150 | 2.123 | 1.630 | 1.784 | 1.707 | 2.020 | 1.922 | 1.971 | 1.376 | 1.400 | 1.388 |
| CO 52 | 2.136 | 2.200 | 2.168 | 1.207 | 1.209 | 1.208 | 0.945 | 0.992 | 0.968 | 0.916 | 0.921 | 0.919 |
| CO 53 | 1.910 | 2.112 | 2.011 | 3.813 | 4.296 | 4.055 | 2.620 | 2.560 | 2.590 | 1.222 | 1.283 | 1.253 |
| CO 54 | 1.748 | 1.823 | 1.785 | 2.100 | 2.210 | 2.155 | 1.860 | 2.114 | 1.987 | 0.372 | 0.361 | 0.367 |
| CO 55 | 1.798 | 1.907 | 1.852 | 1.560 | 1.670 | 1.615 | 1.300 | 1.422 | 1.361 | 0.278 | 0.277 | 0.277 |
| FL 478 | 1.144 | 1.299 | 1.221 | 1.580 | 1.662 | 1.621 | 1.210 | 1.325 | 1.268 | 0.722 | 0.673 | 0.698 |
| FR 13A | 3.080 | 3.171 | 3.125 | 2.953 | 3.182 | 3.067 | 2.312 | 2.447 | 2.379 | 2.580 | 2.623 | 2.601 |
| IR 42 | 0.795 | 0.880 | 0.838 | 1.770 | 1.261 | 1.515 | 1.257 | 1.271 | 1.264 | 0.537 | 0.620 | 0.578 |
| IR 64 | 1.632 | 1.655 | 1.644 | 1.180 | 1.258 | 1.219 | 1.940 | 2.100 | 2.020 | 0.528 | 0.542 | 0.535 |
| IR 64 DRT | 2.383 | 2.518 | 2.450 | 1.634 | 1.705 | 1.669 | 1.520 | 1.448 | 1.484 | 0.618 | 0.636 | 0.627 |
| Kappikar | 2.114 | 2.205 | 2.160 | 2.373 | 2.281 | 2.327 | 2.600 | 2.786 | 2.693 | 0.870 | 0.925 | 0.897 |
| Kattuponni | 2.118 | 2.012 | 2.065 | 1.537 | 1.532 | 1.534 | 2.900 | 3.054 | 2.977 | 1.248 | 1.379 | 1.314 |
| Mattaikar | 1.102 | 1.125 | 1.114 | 1.980 | 2.042 | 2.011 | 2.420 | 2.411 | 2.415 | 2.956 | 2.972 | 2.964 |
| Norungan | 2.438 | 2.561 | 2.500 | 3.490 | 3.874 | 3.682 | 3.935 | 4.151 | 4.043 | 0.754 | 0.785 | 0.770 |
| Ponmani Samba | 1.982 | 2.039 | 2.011 | 0.750 | 0.777 | 0.764 | 0.540 | 0.549 | 0.544 | 1.652 | 1.817 | 1.734 |
| Poongar | 1.546 | 1.750 | 1.648 | 2.910 | 3.098 | 3.004 | 3.410 | 3.746 | 3.578 | 0.162 | 0.168 | 0.165 |
| Upumolagai | 1.582 | 1.528 | 1.555 | 1.853 | 1.870 | 1.862 | 2.715 | 2.811 | 2.763 | 0.334 | 0.351 | 0.343 |
| Vadakathi Samba | 1.670 | 1.724 | 1.697 | 0.697 | 0.707 | 0.702 | 1.685 | 1.576 | 1.630 | 1.032 | 1.054 | 1.043 |
| Varigarudan Samba | 2.218 | 2.270 | 2.244 | 3.110 | 3.280 | 3.195 | 2.025 | 2.062 | 2.044 | 0.374 | 0.367 | 0.370 |
| **Mean** | **1.981** | **2.056** | **2.019** | **1.651** | **1.724** | **1.688** | **1.648** | **1.686** | **1.667** | **0.986** | **1.020** | **1.003** |
| **LSD** | **0.156** | **0.105** | **0.156** | **0.212** | **0.094** | **0.222** | **0.167** | **0.092** | **0.150** | **0.100** | **0.060** | **0.092** |
| **CV** | **4.863** | **3.149** | **3.827** | **7.917** | **3.371** | **6.525** | **6.253** | **3.343** | **4.458** | **6.266** | **3.609** | **4.540** |
| **SE** | **0.056** | **0.037** | **0.055** | **0.076** | **0.034** | **0.078** | **0.060** | **0.033** | **0.053** | **0.036** | **0.021** | **0.032** |
| **Percent Reduction** | **0.000** | **0.000** | **0.000** | **16.640** | **16.163** | **16.394** | **16.796** | **17.991** | **17.434** | **50.252** | **50.389** | **50.322** |

| **Traits** | **Chlorophyll content index** | | | | | | | | | | | |
| --- | --- | --- | --- | --- | --- | --- | --- | --- | --- | --- | --- | --- |
|  | **Control** | | | **Drought** | | | **Salinity** | | | **Submergence** | | |
| **Seasons** | **Kharif** | **Rabi** | **Pooled** | **Kharif** | **Rabi** | **Pooled** | **Kharif** | **Rabi** | **Pooled** | **Kharif** | **Rabi** | **Pooled** |
| ADT 45 | 1.100 | 1.138 | 1.119 | 1.000 | 1.026 | 1.013 | 1.200 | 1.304 | 1.252 | 1.200 | 1.225 | 1.212 |
| ADT 51 | 1.100 | 1.181 | 1.140 | 1.300 | 1.344 | 1.322 | 0.900 | 0.964 | 0.932 | 1.300 | 1.313 | 1.307 |
| ADT 52 | 1.100 | 1.131 | 1.115 | 1.400 | 1.447 | 1.424 | 1.000 | 1.037 | 1.018 | 1.200 | 1.249 | 1.224 |
| ADT 53 | 1.500 | 1.574 | 1.537 | 1.000 | 0.963 | 0.981 | 1.000 | 1.020 | 1.010 | 2.100 | 2.213 | 2.157 |
| ADT 54 | 1.100 | 1.193 | 1.146 | 1.000 | 1.067 | 1.033 | 1.300 | 1.441 | 1.371 | 1.600 | 1.623 | 1.612 |
| ADT 56 | 1.000 | 0.982 | 0.991 | 1.200 | 1.253 | 1.226 | 1.000 | 0.984 | 0.992 | 1.900 | 1.834 | 1.867 |
| ADT 57 | 1.300 | 1.393 | 1.347 | 1.000 | 1.074 | 1.037 | 1.800 | 1.819 | 1.809 | 1.400 | 1.521 | 1.460 |
| ANNA R 4 | 1.200 | 1.271 | 1.235 | 1.300 | 1.315 | 1.308 | 1.100 | 1.058 | 1.079 | 1.800 | 2.004 | 1.902 |
| APD19002 | 1.600 | 1.656 | 1.628 | 1.800 | 1.780 | 1.790 | 1.400 | 1.468 | 1.434 | 1.300 | 1.306 | 1.303 |
| Arupatham samba | 1.200 | 1.240 | 1.220 | 1.400 | 1.542 | 1.471 | 1.000 | 1.036 | 1.018 | 1.900 | 2.157 | 2.028 |
| CB 16656 | 1.200 | 1.214 | 1.207 | 1.300 | 1.312 | 1.306 | 1.000 | 1.039 | 1.020 | 1.500 | 1.440 | 1.470 |
| CB 17502 | 1.300 | 1.423 | 1.362 | 1.700 | 1.737 | 1.719 | 1.000 | 0.992 | 0.996 | 1.700 | 1.805 | 1.753 |
| CB 17542 | 1.300 | 1.291 | 1.295 | 1.700 | 1.726 | 1.713 | 1.000 | 1.106 | 1.053 | 1.500 | 1.586 | 1.543 |
| CB 17561 | 1.300 | 1.331 | 1.316 | 1.300 | 1.232 | 1.266 | 1.000 | 1.038 | 1.019 | 1.400 | 1.434 | 1.417 |
| CB 17573 | 1.500 | 1.638 | 1.569 | 1.200 | 1.245 | 1.222 | 1.400 | 1.343 | 1.371 | 1.400 | 1.484 | 1.442 |
| CB 17597 | 1.000 | 0.959 | 0.980 | 1.100 | 1.107 | 1.103 | 1.300 | 1.372 | 1.336 | 1.200 | 1.263 | 1.231 |
| CB 22504 | 1.000 | 1.055 | 1.027 | 1.400 | 1.439 | 1.419 | 1.200 | 1.125 | 1.162 | 1.800 | 1.836 | 1.818 |
| CB 22512 | 1.000 | 1.008 | 1.004 | 1.100 | 1.116 | 1.108 | 1.000 | 1.026 | 1.013 | 1.500 | 1.399 | 1.449 |
| CB 22541 | 1.400 | 1.467 | 1.434 | 1.000 | 1.003 | 1.001 | 1.100 | 1.119 | 1.110 | 1.400 | 1.424 | 1.412 |
| CB 22560 | 1.300 | 1.332 | 1.316 | 2.200 | 2.289 | 2.245 | 1.000 | 1.033 | 1.017 | 1.300 | 1.370 | 1.335 |
| ChittanSamba | 1.500 | 1.566 | 1.533 | 1.400 | 1.471 | 1.435 | 1.400 | 1.372 | 1.386 | 1.100 | 1.113 | 1.106 |
| CO 49 | 1.600 | 1.630 | 1.615 | 1.200 | 1.228 | 1.214 | 1.200 | 1.337 | 1.268 | 1.200 | 1.327 | 1.264 |
| CO 51 | 1.400 | 1.405 | 1.402 | 1.200 | 1.152 | 1.176 | 1.100 | 1.118 | 1.109 | 1.200 | 1.232 | 1.216 |
| CO 52 | 1.500 | 1.534 | 1.517 | 1.400 | 1.426 | 1.413 | 1.200 | 1.143 | 1.172 | 2.000 | 2.071 | 2.035 |
| CO 53 | 1.400 | 1.462 | 1.431 | 3.200 | 3.288 | 3.244 | 2.100 | 2.008 | 2.054 | 1.500 | 1.583 | 1.542 |
| CO 54 | 1.100 | 1.123 | 1.112 | 1.200 | 1.347 | 1.273 | 1.100 | 1.068 | 1.084 | 1.500 | 1.576 | 1.538 |
| CO 55 | 1.000 | 0.930 | 0.965 | 1.200 | 1.243 | 1.221 | 1.100 | 1.141 | 1.121 | 1.700 | 1.729 | 1.714 |
| FL 478 | 1.200 | 1.234 | 1.217 | 1.300 | 1.287 | 1.294 | 1.400 | 1.487 | 1.443 | 1.400 | 1.493 | 1.446 |
| FR 13A | 1.100 | 1.212 | 1.156 | 1.461 | 1.596 | 1.529 | 1.200 | 1.525 | 1.363 | 2.300 | 2.576 | 2.438 |
| IR 42 | 1.069 | 1.155 | 1.112 | 1.009 | 1.019 | 1.014 | 0.800 | 0.890 | 0.845 | 0.983 | 1.019 | 1.001 |
| IR 64 | 1.100 | 1.155 | 1.128 | 1.100 | 1.167 | 1.133 | 0.900 | 0.890 | 0.895 | 1.000 | 1.071 | 1.035 |
| IR 64 DRT | 1.400 | 1.551 | 1.476 | 1.800 | 1.816 | 1.808 | 1.100 | 1.143 | 1.121 | 1.300 | 1.338 | 1.319 |
| Kappikar | 1.100 | 1.137 | 1.118 | 1.100 | 1.142 | 1.121 | 1.000 | 1.052 | 1.026 | 1.100 | 1.110 | 1.105 |
| Kattuponni | 1.300 | 1.347 | 1.323 | 1.700 | 1.789 | 1.744 | 1.100 | 1.150 | 1.125 | 1.800 | 1.963 | 1.881 |
| Mattaikar | 1.300 | 1.391 | 1.345 | 1.600 | 1.713 | 1.657 | 1.300 | 1.275 | 1.288 | 2.100 | 2.212 | 2.156 |
| Norungan | 1.400 | 1.544 | 1.472 | 2.900 | 3.091 | 2.996 | 1.300 | 1.425 | 1.363 | 2.100 | 1.992 | 2.046 |
| Ponmani Samba | 1.200 | 1.137 | 1.169 | 1.200 | 1.177 | 1.189 | 1.800 | 1.831 | 1.816 | 1.500 | 1.444 | 1.472 |
| Poongar | 1.800 | 1.683 | 1.742 | 1.300 | 1.320 | 1.310 | 1.100 | 1.110 | 1.105 | 1.800 | 1.828 | 1.814 |
| Upumolagai | 1.300 | 1.279 | 1.289 | 1.300 | 1.353 | 1.326 | 1.000 | 1.031 | 1.016 | 1.300 | 1.291 | 1.295 |
| Vadakathi Samba | 1.200 | 1.211 | 1.205 | 1.300 | 1.264 | 1.282 | 1.500 | 1.632 | 1.566 | 1.400 | 1.380 | 1.390 |
| Varigarudan Samba | 1.300 | 1.427 | 1.364 | 1.300 | 1.423 | 1.361 | 1.100 | 1.113 | 1.106 | 1.300 | 1.354 | 1.327 |
| **Mean** | **1.263** | **1.307** | **1.285** | **1.404** | **1.447** | **1.426** | **1.183** | **1.221** | **1.202** | **1.512** | **1.566** | **1.539** |
| **LSD** | **0.110** | **0.064** | **0.104** | **0.113** | **0.087** | **0.100** | **0.113** | **0.079** | **0.117** | **0.143** | **0.077** | **0.138** |
| **CV** | **5.343** | **2.992** | **4.005** | **4.966** | **3.689** | **3.473** | **5.900** | **4.005** | **4.805** | **5.836** | **3.032** | **4.429** |
| **SE** | **0.039** | **0.023** | **0.036** | **0.043** | **0.045** | **0.035** | **0.040** | **0.021** | **0.041** | **0.051** | **0.027** | **0.048** |
| **Percent Reduction** | **0.000** | **0.000** | **0.000** | **-11.205** | **-10.711** | **-10.973** | **6.315** | **6.573** | **6.459** | **-19.730** | **-19.773** | **-19.767** |

| **Traits** | **Relative water content** | | | | | | | | | | | |
| --- | --- | --- | --- | --- | --- | --- | --- | --- | --- | --- | --- | --- |
|  | **Control** | | | **Drought** | | | **Salinity** | | | **Submergence** | | |
| **Seasons** | **Kharif** | **Rabi** | **Pooled** | **Kharif** | **Rabi** | **Pooled** | **Kharif** | **Rabi** | **Pooled** | **Kharif** | **Rabi** | **Pooled** |
| ADT 45 | 53.333 | 57.047 | 55.190 | 16.500 | 17.021 | 16.760 | 22.500 | 23.390 | 22.945 | 15.300 | 16.083 | 15.691 |
| ADT 51 | 35.294 | 37.250 | 36.272 | 26.800 | 27.890 | 27.345 | 18.600 | 20.034 | 19.317 | 20.500 | 22.642 | 21.571 |
| ADT 52 | 50.472 | 55.675 | 53.074 | 28.300 | 28.797 | 28.548 | 22.300 | 24.130 | 23.215 | 28.300 | 29.351 | 28.826 |
| ADT 53 | 42.105 | 42.238 | 42.172 | 14.800 | 14.952 | 14.876 | 15.977 | 25.583 | 20.780 | 15.300 | 16.536 | 15.918 |
| ADT 54 | 49.303 | 52.766 | 51.035 | 33.900 | 35.760 | 34.830 | 25.300 | 26.248 | 25.774 | 26.300 | 27.529 | 26.915 |
| ADT 56 | 51.333 | 53.231 | 52.282 | 25.600 | 26.218 | 25.909 | 26.300 | 27.177 | 26.738 | 16.500 | 15.476 | 15.988 |
| ADT 57 | 47.111 | 50.866 | 48.989 | 18.500 | 18.759 | 18.630 | 20.600 | 22.890 | 21.745 | 24.300 | 25.628 | 24.964 |
| ANNA R 4 | 52.857 | 55.478 | 54.167 | 38.500 | 41.955 | 40.227 | 35.600 | 36.396 | 35.998 | 26.200 | 27.306 | 26.753 |
| APD19002 | 64.724 | 67.277 | 66.000 | 50.300 | 49.163 | 49.731 | 45.800 | 45.213 | 45.506 | 48.600 | 54.438 | 51.519 |
| Arupatham samba | 69.494 | 72.073 | 70.783 | 40.320 | 43.345 | 41.833 | 43.900 | 43.694 | 43.797 | 41.300 | 42.015 | 41.657 |
| CB 16656 | 61.389 | 63.684 | 62.536 | 37.500 | 37.971 | 37.735 | 33.800 | 35.629 | 34.715 | 29.300 | 31.227 | 30.264 |
| CB 17502 | 50.600 | 54.642 | 52.621 | 25.300 | 25.749 | 25.525 | 21.300 | 22.491 | 21.895 | 20.600 | 21.356 | 20.978 |
| CB 17542 | 46.667 | 49.098 | 47.883 | 18.300 | 19.022 | 18.661 | 16.300 | 17.620 | 16.960 | 26.500 | 25.610 | 26.055 |
| CB 17561 | 41.053 | 43.322 | 42.188 | 17.600 | 19.767 | 18.684 | 20.300 | 21.777 | 21.039 | 24.300 | 25.494 | 24.897 |
| CB 17573 | 45.926 | 50.508 | 48.217 | 16.500 | 16.459 | 16.479 | 12.300 | 12.760 | 12.530 | 15.600 | 17.162 | 16.381 |
| CB 17597 | 42.162 | 43.892 | 43.027 | 20.300 | 19.471 | 19.885 | 15.600 | 15.160 | 15.380 | 16.800 | 17.426 | 17.113 |
| CB 22504 | 60.000 | 62.262 | 61.131 | 38.200 | 39.416 | 38.808 | 32.900 | 33.356 | 33.128 | 33.600 | 34.568 | 34.084 |
| CB 22512 | 36.000 | 35.728 | 35.864 | 12.300 | 12.443 | 12.371 | 11.800 | 12.012 | 11.906 | 15.600 | 15.455 | 15.528 |
| CB 22541 | 66.667 | 73.368 | 70.017 | 14.500 | 14.712 | 14.606 | 25.600 | 25.871 | 25.736 | 35.600 | 38.691 | 37.145 |
| CB 22560 | 63.636 | 68.785 | 66.211 | 31.800 | 34.786 | 33.293 | 28.600 | 30.203 | 29.402 | 30.500 | 31.984 | 31.242 |
| ChittanSamba | 51.667 | 51.871 | 51.769 | 14.300 | 15.044 | 14.672 | 13.500 | 14.273 | 13.886 | 19.600 | 21.383 | 20.492 |
| CO 49 | 32.099 | 33.137 | 32.618 | 19.600 | 21.306 | 20.453 | 20.500 | 22.538 | 21.519 | 22.500 | 24.240 | 23.370 |
| CO 51 | 56.667 | 54.983 | 55.825 | 18.300 | 17.668 | 17.984 | 26.300 | 28.363 | 27.331 | 32.100 | 32.227 | 32.163 |
| CO 52 | 53.333 | 54.940 | 54.137 | 33.200 | 35.417 | 34.308 | 23.500 | 23.795 | 23.648 | 28.600 | 28.267 | 28.433 |
| CO 53 | 57.265 | 59.510 | 58.387 | 46.500 | 47.972 | 47.236 | 42.800 | 47.791 | 45.296 | 31.300 | 32.179 | 31.739 |
| CO 54 | 53.846 | 55.541 | 54.694 | 18.170 | 18.514 | 18.342 | 24.300 | 22.649 | 23.475 | 28.500 | 28.744 | 28.622 |
| CO 55 | 45.455 | 47.524 | 46.490 | 21.300 | 21.559 | 21.429 | 21.900 | 23.559 | 22.729 | 24.200 | 24.729 | 24.464 |
| FL 478 | 55.846 | 59.012 | 57.429 | 46.800 | 46.502 | 46.651 | 45.600 | 46.003 | 45.801 | 38.300 | 36.409 | 37.355 |
| FR 13A | 60.333 | 58.233 | 59.283 | 44.300 | 45.733 | 45.017 | 35.500 | 36.141 | 35.821 | 53.100 | 53.866 | 53.483 |
| IR 42 | 45.015 | 45.798 | 45.407 | 16.671 | 18.606 | 17.639 | 19.221 | 20.491 | 19.856 | 25.005 | 26.874 | 25.940 |
| IR 64 | 23.529 | 24.138 | 23.834 | 21.100 | 22.301 | 21.700 | 22.400 | 23.913 | 23.157 | 33.500 | 35.877 | 34.689 |
| IR 64 DRT | 57.143 | 59.496 | 58.319 | 42.300 | 43.726 | 43.013 | 38.600 | 35.971 | 37.285 | 35.200 | 35.765 | 35.482 |
| Kappikar | 47.500 | 48.079 | 47.790 | 15.600 | 15.858 | 15.729 | 12.900 | 12.780 | 12.840 | 13.200 | 13.343 | 13.272 |
| Kattuponni | 38.919 | 40.949 | 39.934 | 16.500 | 17.408 | 16.954 | 20.300 | 21.355 | 20.827 | 25.600 | 26.439 | 26.020 |
| Mattaikar | 67.143 | 66.917 | 67.030 | 48.600 | 50.466 | 49.533 | 44.600 | 44.176 | 44.388 | 48.320 | 49.136 | 48.728 |
| Norungan | 57.143 | 54.381 | 55.762 | 39.900 | 40.394 | 40.147 | 36.800 | 37.101 | 36.951 | 34.800 | 34.101 | 34.450 |
| Ponmani Samba | 41.739 | 43.329 | 42.534 | 16.300 | 15.952 | 16.126 | 20.300 | 20.466 | 20.383 | 21.300 | 20.577 | 20.939 |
| Poongar | 55.000 | 56.958 | 55.979 | 12.600 | 12.978 | 12.789 | 15.300 | 16.422 | 15.861 | 20.300 | 19.585 | 19.943 |
| Upumolagai | 43.500 | 45.012 | 44.256 | 10.600 | 10.299 | 10.449 | 10.800 | 11.341 | 11.071 | 20.800 | 22.193 | 21.496 |
| Vadakathi Samba | 37.500 | 41.435 | 39.467 | 12.600 | 12.872 | 12.736 | 18.900 | 19.132 | 19.016 | 20.100 | 22.144 | 21.122 |
| Varigarudan Samba | 44.000 | 49.288 | 46.644 | 12.300 | 12.830 | 12.565 | 12.800 | 13.464 | 13.132 | 10.600 | 10.497 | 10.549 |
| **Mean** | **50.116** | **52.188** | **51.152** | **25.692** | **26.514** | **26.103** | **24.932** | **25.936** | **25.434** | **26.779** | **27.672** | **27.225** |
| **LSD** | **3.804** | **2.556** | **4.061** | **1.785** | **1.405** | **1.874** | **2.432** | **1.424** | **2.931** | **2.425** | **1.414** | **2.232** |
| **CV** | **4.673** | **3.015** | **3.932** | **4.277** | **3.262** | **3.555** | **6.005** | **3.381** | **5.707** | **5.575** | **3.146** | **4.059** |
| **SE** | **1.352** | **0.908** | **1.422** | **0.634** | **0.499** | **0.656** | **0.864** | **0.735** | **1.026** | **0.862** | **0.503** | **0.784** |
| **Percent Reduction** | **0.000** | **0.000** | **0.000** | **48.736** | **49.196** | **48.970** | **50.252** | **50.304** | **50.278** | **46.567** | **46.977** | **46.776** |
