# Supplementary material for: Enhancing stress resilience in rice (Oryza sativa L.) through profiling early-stage morpho-physiological and molecular responses to multiple abiotic stress tolerance
Source: Front Plant Sci. 2024 Feb 8;15:1342441. doi: 10.3389/fpls.2024.1342441 (PMC10882102; doi:10.3389/fpls.2024.1342441)
Supplement: Supplementary file 5 [file Table_5.docx]

Supplementary table 5. Chlorophyll fluorescence parameters

| **Traits** | **Fo** | | | | | | | | | | | |
| --- | --- | --- | --- | --- | --- | --- | --- | --- | --- | --- | --- | --- |
|  | **Control** | | | **Drought** | | | **Salinity** | | | **Submergence** | | |
| **Seasons** | **Kharif** | **Rabi** | **Pooled** | **Kharif** | **Rabi** | **Pooled** | **Kharif** | **Rabi** | **Pooled** | **Kharif** | **Rabi** | **Pooled** |
| ADT 45 | 66.000 | 63.539 | 64.769 | 60.000 | 62.192 | 61.096 | 62.000 | 60.123 | 61.062 | 41.000 | 43.393 | 42.196 |
| ADT 51 | 46.000 | 50.596 | 48.298 | 65.000 | 67.825 | 66.412 | 55.000 | 56.427 | 55.714 | 54.000 | 57.493 | 55.746 |
| ADT 52 | 56.000 | 57.490 | 56.745 | 66.000 | 72.016 | 69.008 | 68.000 | 63.906 | 65.953 | 44.000 | 45.574 | 44.787 |
| ADT 53 | 47.000 | 49.496 | 48.248 | 37.000 | 35.684 | 36.342 | 35.000 | 38.089 | 36.545 | 72.000 | 76.046 | 74.023 |
| ADT 54 | 92.000 | 95.828 | 93.914 | 79.000 | 82.913 | 80.956 | 75.000 | 79.392 | 77.196 | 45.000 | 44.079 | 44.540 |
| ADT 56 | 49.000 | 50.152 | 49.576 | 74.000 | 76.027 | 75.013 | 70.000 | 69.283 | 69.642 | 48.000 | 50.110 | 49.055 |
| ADT 57 | 54.000 | 54.189 | 54.095 | 81.000 | 89.558 | 85.279 | 82.000 | 85.986 | 83.993 | 74.000 | 76.130 | 75.065 |
| ANNA R 4 | 39.000 | 39.879 | 39.439 | 52.000 | 52.146 | 52.073 | 56.000 | 57.664 | 56.832 | 60.000 | 59.464 | 59.732 |
| APD19002 | 69.000 | 69.951 | 69.475 | 74.000 | 71.441 | 72.721 | 74.000 | 76.723 | 75.361 | 53.000 | 57.808 | 55.404 |
| Arupatham samba | 59.000 | 66.340 | 62.670 | 69.000 | 68.077 | 68.539 | 65.000 | 62.716 | 63.858 | 51.000 | 54.441 | 52.720 |
| CB 16656 | 57.000 | 59.962 | 58.481 | 72.000 | 70.939 | 71.469 | 70.000 | 78.067 | 74.034 | 42.000 | 45.006 | 43.503 |
| CB 17502 | 43.000 | 47.676 | 45.338 | 59.000 | 59.950 | 59.475 | 52.000 | 49.886 | 50.943 | 49.000 | 50.861 | 49.931 |
| CB 17542 | 39.000 | 38.311 | 38.655 | 93.000 | 90.973 | 91.987 | 95.000 | 96.762 | 95.881 | 40.000 | 42.965 | 41.483 |
| CB 17561 | 43.000 | 45.020 | 44.010 | 48.000 | 49.812 | 48.906 | 46.000 | 46.590 | 46.295 | 38.000 | 36.760 | 37.380 |
| CB 17573 | 38.000 | 39.785 | 38.893 | 67.000 | 68.130 | 67.565 | 66.000 | 67.596 | 66.798 | 63.000 | 67.282 | 65.141 |
| CB 17597 | 71.000 | 72.908 | 71.954 | 94.000 | 96.151 | 95.075 | 93.000 | 96.737 | 94.869 | 45.000 | 46.034 | 45.517 |
| CB 22504 | 43.000 | 48.078 | 45.539 | 51.000 | 55.638 | 53.319 | 50.000 | 48.250 | 49.125 | 38.000 | 36.822 | 37.411 |
| CB 22512 | 53.000 | 57.725 | 55.362 | 57.000 | 53.703 | 55.351 | 55.000 | 55.101 | 55.050 | 43.000 | 46.374 | 44.687 |
| CB 22541 | 48.000 | 49.015 | 48.508 | 63.000 | 60.090 | 61.545 | 61.000 | 64.073 | 62.537 | 45.000 | 47.369 | 46.184 |
| CB 22560 | 50.000 | 51.504 | 50.752 | 43.000 | 43.373 | 43.187 | 40.000 | 39.607 | 39.803 | 45.000 | 48.338 | 46.669 |
| ChittanSamba | 71.000 | 74.706 | 72.853 | 48.000 | 53.173 | 50.587 | 48.000 | 49.920 | 48.960 | 43.000 | 46.823 | 44.911 |
| CO 49 | 44.000 | 44.200 | 44.100 | 47.000 | 49.457 | 48.229 | 50.000 | 52.015 | 51.007 | 49.000 | 54.358 | 51.679 |
| CO 51 | 107.000 | 111.976 | 109.488 | 50.000 | 52.340 | 51.170 | 48.000 | 48.436 | 48.218 | 42.000 | 42.376 | 42.188 |
| CO 52 | 54.000 | 55.180 | 54.590 | 62.000 | 58.879 | 60.439 | 68.000 | 73.950 | 70.975 | 39.000 | 39.545 | 39.273 |
| CO 53 | 97.000 | 109.102 | 103.051 | 41.000 | 46.374 | 43.687 | 50.000 | 51.951 | 50.975 | 46.000 | 49.605 | 47.803 |
| CO 54 | 48.000 | 48.457 | 48.228 | 54.000 | 55.727 | 54.863 | 58.000 | 58.727 | 58.364 | 45.000 | 46.386 | 45.693 |
| CO 55 | 59.000 | 59.624 | 59.312 | 64.000 | 60.659 | 62.329 | 60.000 | 62.669 | 61.335 | 45.000 | 47.175 | 46.088 |
| FL 478 | 50.000 | 54.423 | 52.212 | 106.000 | 106.959 | 106.479 | 113.000 | 111.062 | 112.031 | 63.000 | 64.641 | 63.821 |
| FR 13A | 56.000 | 57.363 | 56.682 | 53.000 | 59.460 | 56.230 | 109.000 | 111.650 | 110.325 | 51.000 | 56.165 | 53.583 |
| IR 42 | 51.273 | 52.510 | 51.891 | 51.273 | 52.557 | 51.915 | 50.290 | 51.825 | 51.058 | 52.742 | 53.880 | 53.311 |
| IR 64 | 54.000 | 52.510 | 53.255 | 52.000 | 52.557 | 52.279 | 50.000 | 51.825 | 50.912 | 52.000 | 53.880 | 52.940 |
| IR 64 DRT | 55.000 | 57.344 | 56.172 | 64.000 | 64.996 | 64.498 | 65.000 | 63.045 | 64.022 | 49.000 | 52.919 | 50.959 |
| Kappikar | 78.000 | 78.863 | 78.431 | 64.000 | 63.749 | 63.874 | 63.000 | 66.117 | 64.559 | 64.000 | 62.280 | 63.140 |
| Kattuponni | 63.000 | 60.820 | 61.910 | 107.000 | 114.959 | 110.979 | 95.000 | 90.380 | 92.690 | 62.000 | 62.994 | 62.497 |
| Mattaikar | 61.000 | 66.166 | 63.583 | 78.000 | 83.644 | 80.822 | 80.000 | 76.881 | 78.440 | 44.000 | 44.046 | 44.023 |
| Norungan | 93.000 | 98.611 | 95.805 | 58.000 | 62.711 | 60.355 | 56.000 | 55.437 | 55.718 | 61.000 | 63.621 | 62.310 |
| Ponmani Samba | 58.000 | 60.457 | 59.228 | 59.000 | 60.986 | 59.993 | 55.000 | 51.376 | 53.188 | 73.000 | 82.921 | 77.961 |
| Poongar | 64.000 | 64.931 | 64.465 | 80.000 | 86.587 | 83.294 | 80.000 | 86.825 | 83.412 | 58.000 | 58.779 | 58.389 |
| Upumolagai | 52.000 | 58.899 | 55.449 | 46.000 | 45.551 | 45.776 | 45.000 | 49.179 | 47.090 | 54.000 | 58.942 | 56.471 |
| Vadakathi Samba | 45.000 | 49.218 | 47.109 | 81.000 | 83.348 | 82.174 | 75.000 | 76.281 | 75.641 | 50.000 | 48.606 | 49.303 |
| Varigarudan Samba | 66.000 | 67.169 | 66.584 | 69.000 | 70.371 | 69.686 | 65.000 | 64.711 | 64.856 | 55.000 | 56.590 | 55.795 |
| **Mean** | **58.251** | **60.731** | **59.491** | **64.348** | **66.139** | **65.243** | **64.714** | **65.786** | **65.250** | **50.921** | **53.143** | **52.032** |
| **LSD** | **4.893** | **3.029** | **5.257** | **5.403** | **3.281** | **5.067** | **5.360** | **3.273** | **4.349** | **4.278** | **2.600** | **4.490** |
| **CV** | **5.172** | **3.071** | **4.375** | **5.169** | **3.054** | **3.845** | **5.100** | **3.063** | **3.300** | **5.172** | **3.012** | **4.273** |
| **SE** | **1.734** | **1.077** | **1.841** | **1.920** | **1.166** | **1.774** | **1.905** | **1.163** | **1.523** | **1.520** | **0.924** | **1.572** |
| **Percent Reduction** | **0.000** | **0.000** | **0.000** | **-10.468** | **-8.904** | **-9.669** | **-11.097** | **-8.324** | **-9.680** | **12.584** | **12.494** | **12.538** |

| **Traits** | **FM** | | | | | | | | | | | | | | | | | | | | | | |
| --- | --- | --- | --- | --- | --- | --- | --- | --- | --- | --- | --- | --- | --- | --- | --- | --- | --- | --- | --- | --- | --- | --- | --- |
|  | **Control** | | | | | **Drought** | | | | | | **Salinity** | | | | | | **Submergence** | | | | | |
| **Seasons** | **Kharif** | **Rabi** | | | **Pooled** | **Kharif** | | **Rabi** | | **Pooled** | | **Kharif** | | **Rabi** | | **Pooled** | | **Kharif** | | **Rabi** | | **Pooled** | |
| ADT 45 | 143.000 | 157.094 | | | 150.047 | 212.000 | | 209.907 | | 210.953 | | 202.000 | | 199.222 | | 200.611 | | 91.000 | | 94.784 | | 92.892 | |
| ADT 51 | 121.000 | 126.671 | | | 123.836 | 159.000 | | 169.282 | | 164.141 | | 155.000 | | 159.175 | | 157.087 | | 147.000 | | 153.939 | | 150.470 | |
| ADT 52 | 189.000 | 195.735 | | | 192.367 | 140.000 | | 142.752 | | 141.376 | | 138.000 | | 141.180 | | 139.590 | | 160.000 | | 153.058 | | 156.529 | |
| ADT 53 | 140.000 | 144.721 | | | 142.360 | 101.000 | | 100.146 | | 100.573 | | 108.000 | | 111.745 | | 109.873 | | 165.000 | | 180.370 | | 172.685 | |
| ADT 54 | 226.000 | 226.503 | | | 226.251 | 138.000 | | 144.416 | | 141.208 | | 152.000 | | 154.712 | | 153.356 | | 105.000 | | 107.076 | | 106.038 | |
| ADT 56 | 101.000 | 106.266 | | | 103.633 | 136.000 | | 142.436 | | 139.218 | | 143.000 | | 148.949 | | 145.974 | | 147.000 | | 155.082 | | 151.041 | |
| ADT 57 | 136.000 | 138.308 | | | 137.154 | 133.000 | | 129.473 | | 131.236 | | 123.000 | | 138.176 | | 130.588 | | 177.000 | | 179.721 | | 178.361 | |
| ANNA R 4 | 46.000 | 47.101 | | | 46.551 | 144.000 | | 152.764 | | 148.382 | | 145.000 | | 150.883 | | 147.941 | | 180.000 | | 189.101 | | 184.551 | |
| APD19002 | 148.000 | 143.905 | | | 145.953 | 141.000 | | 153.281 | | 147.141 | | 148.000 | | 151.328 | | 149.664 | | 128.000 | | 141.023 | | 134.512 | |
| Arupatham samba | 198.000 | 185.607 | | | 191.803 | 205.000 | | 223.581 | | 214.291 | | 204.000 | | 220.764 | | 212.382 | | 192.000 | | 216.081 | | 204.041 | |
| CB 16656 | 168.000 | 171.525 | | | 169.763 | 215.000 | | 222.935 | | 218.968 | | 225.000 | | 239.280 | | 232.140 | | 113.000 | | 117.015 | | 115.007 | |
| CB 17502 | 139.000 | 141.892 | | | 140.446 | 141.000 | | 160.086 | | 150.543 | | 146.000 | | 144.947 | | 145.473 | | 159.000 | | 161.092 | | 160.046 | |
| CB 17542 | 142.000 | 142.161 | | | 142.081 | 263.000 | | 293.104 | | 278.052 | | 278.000 | | 283.946 | | 280.973 | | 176.000 | | 186.061 | | 181.031 | |
| CB 17561 | 151.000 | 152.396 | | | 151.698 | 122.000 | | 130.760 | | 126.380 | | 135.000 | | 137.634 | | 136.317 | | 142.000 | | 146.860 | | 144.430 | |
| CB 17573 | 140.000 | 153.418 | | | 146.709 | 177.000 | | 182.179 | | 179.589 | | 180.000 | | 177.357 | | 178.678 | | 141.000 | | 138.292 | | 139.646 | |
| CB 17597 | 191.000 | 181.307 | | | 186.154 | 174.000 | | 183.985 | | 178.992 | | 178.000 | | 193.182 | | 185.591 | | 107.000 | | 109.386 | | 108.193 | |
| CB 22504 | 153.000 | 156.641 | | | 154.820 | 147.000 | | 143.672 | | 145.336 | | 150.000 | | 157.567 | | 153.784 | | 157.000 | | 158.440 | | 157.720 | |
| CB 22512 | 161.000 | 168.495 | | | 164.747 | 142.000 | | 147.635 | | 144.817 | | 138.000 | | 144.692 | | 141.346 | | 129.000 | | 132.710 | | 130.855 | |
| CB 22541 | 157.000 | 155.289 | | | 156.145 | 142.000 | | 145.792 | | 143.896 | | 140.000 | | 143.391 | | 141.695 | | 165.000 | | 161.803 | | 163.401 | |
| CB 22560 | 177.000 | 194.954 | | | 185.977 | 127.000 | | 134.110 | | 130.555 | | 132.000 | | 139.006 | | 135.503 | | 169.000 | | 176.012 | | 172.506 | |
| ChittanSamba | 207.000 | 201.269 | | | 204.134 | 153.000 | | 161.328 | | 157.164 | | 136.000 | | 143.722 | | 139.861 | | 148.000 | | 155.569 | | 151.784 | |
| CO 49 | 116.000 | 117.502 | | | 116.751 | 100.000 | | 107.054 | | 103.527 | | 105.000 | | 107.604 | | 106.302 | | 130.000 | | 143.190 | | 136.595 | |
| CO 51 | 195.000 | 202.619 | | | 198.810 | 165.000 | | 166.318 | | 165.659 | | 170.000 | | 185.004 | | 177.502 | | 111.000 | | 115.029 | | 113.014 | |
| CO 52 | 165.000 | 172.154 | | | 168.577 | 162.000 | | 166.915 | | 164.457 | | 155.000 | | 160.759 | | 157.879 | | 178.000 | | 180.276 | | 179.138 | |
| CO 53 | 159.000 | 163.391 | | | 161.196 | 88.000 | | 90.529 | | 89.265 | | 105.000 | | 110.533 | | 107.767 | | 163.000 | | 179.498 | | 171.249 | |
| CO 54 | 161.000 | 165.286 | | | 163.143 | 164.000 | | 168.166 | | 166.083 | | 160.000 | | 165.862 | | 162.931 | | 113.000 | | 107.655 | | 110.328 | |
| CO 55 | 147.000 | 151.489 | | | 149.244 | 164.000 | | 178.677 | | 171.338 | | 160.000 | | 162.321 | | 161.161 | | 124.000 | | 119.396 | | 121.698 | |
| FL 478 | 192.000 | 204.801 | | | 198.400 | 265.000 | | 273.781 | | 269.390 | | 256.000 | | 248.430 | | 252.215 | | 173.000 | | 174.332 | | 173.666 | |
| FR 13A | 162.000 | 165.596 | | | 163.798 | 182.000 | | 189.794 | | 185.897 | | 192.000 | | 198.173 | | 195.086 | | 150.000 | | 140.929 | | 145.465 | |
| IR 42 | 192.056 | 203.857 | | | 197.957 | 127.941 | | 153.437 | | 140.689 | | 134.711 | | 142.931 | | 138.821 | | 177.677 | | 176.927 | | 177.302 | |
| IR 64 | 200.000 | 203.857 | | | 201.929 | 140.000 | | 153.437 | | 146.719 | | 135.000 | | 143.826 | | 139.413 | | 176.000 | | 170.845 | | 173.423 | |
| IR 64 DRT | 220.000 | 224.895 | | | 222.447 | 139.000 | | 147.476 | | 143.238 | | 140.000 | | 142.156 | | 141.078 | | 177.000 | | 181.310 | | 179.155 | |
| Kappikar | 216.000 | 239.108 | | | 227.554 | 203.000 | | 215.631 | | 209.316 | | 204.000 | | 217.352 | | 210.676 | | 185.000 | | 182.714 | | 183.857 | |
| Kattuponni | 171.000 | 178.731 | | | 174.865 | 271.000 | | 267.640 | | 269.320 | | 252.000 | | 253.501 | | 252.751 | | 216.000 | | 228.349 | | 222.174 | |
| Mattaikar | 191.000 | 183.058 | | | 187.029 | 151.000 | | 142.283 | | 146.642 | | 152.000 | | 169.432 | | 160.716 | | 216.000 | | 206.618 | | 211.309 | |
| Norungan | 207.000 | 199.275 | | | 203.138 | 153.000 | | 158.127 | | 155.563 | | 149.000 | | 156.144 | | 152.572 | | 279.000 | | 292.214 | | 285.607 | |
| Ponmani Samba | 168.000 | 190.096 | | | 179.048 | 148.000 | | 153.137 | | 150.569 | | 148.000 | | 155.390 | | 151.695 | | 247.000 | | 249.440 | | 248.220 | |
| Poongar | 187.000 | 181.898 | | | 184.449 | 203.000 | | 211.005 | | 207.003 | | 202.000 | | 216.916 | | 209.458 | | 190.000 | | 184.096 | | 187.048 | |
| Upumolagai | 145.000 | 142.559 | | | 143.779 | 138.000 | | 136.645 | | 137.322 | | 150.000 | | 160.982 | | 155.491 | | 202.000 | | 218.857 | | 210.429 | |
| Vadakathi Samba | 126.000 | 120.487 | | | 123.243 | 272.000 | | 287.805 | | 279.902 | | 260.000 | | 271.310 | | 265.655 | | 176.000 | | 186.215 | | 181.108 | |
| Varigarudan Samba | 183.000 | 203.031 | | | 193.015 | 201.000 | | 202.541 | | 201.771 | | 196.000 | | 201.386 | | 198.693 | | 198.000 | | 196.706 | | 197.353 | |
| **Mean** | **164.318** | **168.413** | | | **166.366** | **164.608** | | **171.805** | | **168.207** | | **165.408** | | **171.972** | | **168.690** | | **162.919** | | **167.026** | | **164.973** | |
| **LSD** | **14.650** | **8.271** | | | **13.043** | **13.369** | | **8.551** | | **14.916** | | **12.021** | | **8.504** | | **12.270** | | **17.083** | | **8.245** | | **12.308** | |
| **CV** | **5.489** | **3.023** | | | **3.882** | **5.000** | | **3.064** | | **4.391** | | **4.474** | | **3.045** | | **3.602** | | **6.456** | | **3.039** | | **3.694** | |
| **SE** | **5.207** | **1.940** | | | **4.566** | **4.752** | | **3.039** | | **5.222** | | **4.273** | | **3.022** | | **4.296** | | **6.072** | | **2.931** | | **4.309** | |
| **Percent Reduction** | **0.000** | **0.000** | | | **0.000** | **-0.176** | | **-2.014** | | **-1.107** | | **-0.663** | | **-2.113** | | **-1.397** | | **0.852** | | **0.824** | | **0.837** | |
| **Traits** | **FV FM** | | | | | | | | | | | | | | | | | | | | | |  |
|  | **Control** | | | | | **Drought** | | | | | **Salinity** | | | | | | **Submergence** | | | | | |  |
| **Seasons** | **Kharif** | | **Rabi** | **Pooled** | | **Kharif** | **Rabi** | | **Pooled** | | **Kharif** | | **Rabi** | | **Pooled** | | **Kharif** | | **Rabi** | | **Pooled** | |  |
| ADT 45 | 0.538 | | 0.552 | 0.545 | | 0.665 | 0.693 | | 0.705 | | 0.702 | | 0.660 | | 0.690 | | 0.581 | | 0.577 | | 0.579 | |  |
| ADT 51 | 0.619 | | 0.632 | 0.626 | | 0.591 | 0.571 | | 0.581 | | 0.597 | | 0.519 | | 0.558 | | 0.637 | | 0.644 | | 0.640 | |  |
| ADT 52 | 0.703 | | 0.727 | 0.715 | | 0.528 | 0.557 | | 0.542 | | 0.531 | | 0.633 | | 0.582 | | 0.632 | | 0.642 | | 0.637 | |  |
| ADT 53 | 0.664 | | 0.696 | 0.680 | | 0.633 | 0.641 | | 0.637 | | 0.621 | | 0.409 | | 0.515 | | 0.585 | | 0.613 | | 0.599 | |  |
| ADT 54 | 0.592 | | 0.588 | 0.590 | | 0.427 | 0.422 | | 0.424 | | 0.421 | | 0.442 | | 0.432 | | 0.725 | | 0.734 | | 0.730 | |  |
| ADT 56 | 0.514 | | 0.531 | 0.523 | | 0.455 | 0.464 | | 0.460 | | 0.457 | | 0.339 | | 0.398 | | 0.571 | | 0.556 | | 0.564 | |  |
| ADT 57 | 0.602 | | 0.584 | 0.593 | | 0.390 | 0.442 | | 0.416 | | 0.340 | | 0.635 | | 0.487 | | 0.673 | | 0.685 | | 0.679 | |  |
| ANNA R 4 | 0.152 | | 0.157 | 0.155 | | 0.638 | 0.661 | | 0.649 | | 0.613 | | 0.621 | | 0.617 | | 0.623 | | 0.704 | | 0.663 | |  |
| APD19002 | 0.533 | | 0.584 | 0.558 | | 0.475 | 0.500 | | 0.488 | | 0.435 | | 0.418 | | 0.426 | | 0.549 | | 0.558 | | 0.553 | |  |
| Arupatham samba | 0.702 | | 0.671 | 0.686 | | 0716 | 0.750 | | 0.708 | | 0.632 | | 0.633 | | 0.632 | | 0.628 | | 0.658 | | 0.643 | |  |
| CB 16656 | 0.660 | | 0.672 | 0.666 | | 0.661 | 0.642 | | 0.652 | | 0.661 | | 0.682 | | 0.671 | | 0.733 | | 0.754 | | 0.744 | |  |
| CB 17502 | 0.690 | | 0.718 | 0.704 | | 0.581 | 0.612 | | 0.597 | | 0.592 | | 0.625 | | 0.608 | | 0.666 | | 0.739 | | 0.702 | |  |
| CB 17542 | 0.725 | | 0.739 | 0.732 | | 0.646 | 0.666 | | 0.656 | | 0.634 | | 0.662 | | 0.648 | | 0.691 | | 0.668 | | 0.679 | |  |
| CB 17561 | 0.715 | | 0.693 | 0.704 | | 0.606 | 0.652 | | 0.629 | | 0.616 | | 0.698 | | 0.657 | | 0.772 | | 0.718 | | 0.745 | |  |
| CB 17573 | 0.728 | | 0.701 | 0.715 | | 0.621 | 0.635 | | 0.628 | | 0.632 | | 0.665 | | 0.648 | | 0.732 | | 0.719 | | 0.726 | |  |
| CB 17597 | 0.628 | | 0.628 | 0.628 | | 0.459 | 0.463 | | 0.461 | | 0.492 | | 0.501 | | 0.497 | | 0.553 | | 0.580 | | 0.566 | |  |
| CB 22504 | 0.718 | | 0.738 | 0.728 | | 0.658 | 0.664 | | 0.661 | | 0.624 | | 0.647 | | 0.636 | | 0.579 | | 0.586 | | 0.582 | |  |
| CB 22512 | 0.670 | | 0.693 | 0.681 | | 0.598 | 0.615 | | 0.607 | | 0.592 | | 0.614 | | 0.603 | | 0.757 | | 0.830 | | 0.794 | |  |
| CB 22541 | 0.694 | | 0.787 | 0.741 | | 0.556 | 0.544 | | 0.550 | | 0.516 | | 0.529 | | 0.522 | | 0.666 | | 0.684 | | 0.675 | |  |
| CB 22560 | 0.717 | | 0.722 | 0.720 | | 0.661 | 0.690 | | 0.676 | | 0.652 | | 0.612 | | 0.632 | | 0.727 | | 0.709 | | 0.718 | |  |
| ChittanSamba | 0.657 | | 0.721 | 0.689 | | 0.686 | 0.684 | | 0.685 | | 0.659 | | 0.686 | | 0.672 | | 0.732 | | 0.768 | | 0.750 | |  |
| CO 49 | 0.620 | | 0.633 | 0.626 | | 0.530 | 0.556 | | 0.543 | | 0.526 | | 0.519 | | 0.522 | | 0.563 | | 0.614 | | 0.589 | |  |
| CO 51 | 0.451 | | 0.450 | 0.451 | | 0.696 | 0.753 | | 0.724 | | 0.646 | | 0.695 | | 0.670 | | 0.646 | | 0.716 | | 0.681 | |  |
| CO 52 | 0.672 | | 0.704 | 0.688 | | 0.617 | 0.669 | | 0.643 | | 0.597 | | 0.631 | | 0.614 | | 0.621 | | 0.629 | | 0.625 | |  |
| CO 53 | 0.389 | | 0.429 | 0.409 | | 0.534 | 0.554 | | 0.544 | | 0.625 | | 0.669 | | 0.647 | | 0.780 | | 0.762 | | 0.771 | |  |
| CO 54 | 0.701 | | 0.779 | 0.740 | | 0.670 | 0.691 | | 0.681 | | 0.652 | | 0.640 | | 0.646 | | 0.717 | | 0.738 | | 0.727 | |  |
| CO 55 | 0.598 | | 0.581 | 0.589 | | 0.609 | 0.614 | | 0.612 | | 0.612 | | 0.634 | | 0.623 | | 0.601 | | 0.629 | | 0.615 | |  |
| FL 478 | 0.739 | | 0.763 | 0.751 | | 0.600 | 0.576 | | 0.588 | | 0.612 | | 0.628 | | 0.620 | | 0.712 | | 0.681 | | 0.696 | |  |
| FR 13A | 0.654 | | 0.660 | 0.657 | | 0.708 | 0.741 | | 0.724 | | 0.715 | | 0.741 | | 0.728 | | 0.635 | | 0.834 | | 0.734 | |  |
| IR 42 | 0.517 | | 0.786 | 0.652 | | 0.441 | 0.624 | | 0.533 | | 0.602 | | 0.575 | | 0.588 | | 0.590 | | 0.072 | | 0.331 | |  |
| IR 64 | 0.730 | | 0.786 | 0.758 | | 0.628 | 0.624 | | 0.626 | | 0.602 | | 0.575 | | 0.588 | | 0.723 | | 0.724 | | 0.723 | |  |
| IR 64 DRT1 | 0.750 | | 0.786 | 0.768 | | 0.539 | 0.557 | | 0.548 | | 0.469 | | 0.485 | | 0.477 | | 0.660 | | 0.692 | | 0.676 | |  |
| Kappikar | 0.638 | | 0.647 | 0.643 | | 0.684 | 0.682 | | 0.683 | | 0.635 | | 0.632 | | 0.633 | | 0.694 | | 0.753 | | 0.723 | |  |
| Kattuponni | 0.631 | | 0.668 | 0.650 | | 0.605 | 0.633 | | 0.619 | | 0.615 | | 0.631 | | 0.623 | | 0.715 | | 0.738 | | 0.727 | |  |
| Mattaikar | 0.680 | | 0.662 | 0.671 | | 0.483 | 0.503 | | 0.493 | | 0.453 | | 0.476 | | 0.465 | | 0.781 | | 0.824 | | 0.764 | |  |
| Norungan | 0.550 | | 0.573 | 0.561 | | 0.620 | 0.703 | | 0.662 | | 0.630 | | 0.657 | | 0.643 | | 0.709 | | 0.726 | | 0.717 | |  |
| Ponmani Samba | 0.654 | | 0.671 | 0.663 | | 0.601 | 0.592 | | 0.597 | | 0.612 | | 0.590 | | 0.601 | | 0.701 | | 0.734 | | 0.758 | |  |
| Poongar | 0.657 | | 0.702 | 0.679 | | 0.605 | 0.666 | | 0.636 | | 0.621 | | 0.634 | | 0.628 | | 0.734 | | 0.709 | | 0.722 | |  |
| Upumolagai | 0.641 | | 0.675 | 0.658 | | 0.666 | 0.667 | | 0.667 | | 0.686 | | 0.695 | | 0.690 | | 0.722 | | 0.750 | | 0.736 | |  |
| Vadakathi Samba | 0.642 | | 0.671 | 0.657 | | 0.702 | 0.690 | | 0.696 | | 0.720 | | 0.692 | | 0.697 | | 0.704 | | 0.768 | | 0.736 | |  |
| Varigarudan Samba | 0.639 | | 0.658 | 0.649 | | 0.656 | 0.674 | | 0.665 | | 0.639 | | 0.753 | | 0.696 | | 0.654 | | 0.695 | | 0.674 | |  |
| **Mean** | **0.629** | | **0.654** | **0.641** | | **0.596** | **0.618** | | **0.607** | | **0.592** | | **0.604** | | **0.598** | | **0.670** | | **0.681** | | **0.675** | |  |
| **LSD** | **0.111** | | **0.032** | **0.076** | | **0.093** | **0.030** | | **0.060** | | **0.047** | | **0.029** | | **0.101** | | **0.134** | | **0.033** | | **0.136** | |  |
| **CV** | **10.879** | | **3.001** | **5.863** | | **9.645** | **2.983** | | **4.932** | | **4.848** | | **2.991** | | **8.350** | | **12.337** | | **3.001** | | **9.992** | |  |
| **SE** | **0.040** | | **0.011** | **0.027** | | **0.033** | **0.011** | | **0.021** | | **0.016** | | **0.010** | | **0.035** | | **0.047** | | **0.012** | | **0.048** | |  |
| **Percent Reduction** | **0.000** | | **0.000** | **0.000** | | **5.157** | **5.533** | | **5.304** | | **5.767** | | **7.619** | | **6.708** | | **-6.607** | | **-4.059** | | **-5.304** | |  |

| **Traits** | **Fv Fo** | | | | | | | | | | | |
| --- | --- | --- | --- | --- | --- | --- | --- | --- | --- | --- | --- | --- |
|  | **Control** | | | **Drought** | | | **Salinity** | | | **Submergence** | | |
| **Seasons** | **Kharif** | **Rabi** | **Pooled** | **Kharif** | **Rabi** | **Pooled** | **Kharif** | **Rabi** | **Pooled** | **Kharif** | **Rabi** | **Pooled** |
| ADT 45 | 1.166 | 1.200 | 1.183 | 2.533 | 2.605 | 2.569 | 2.140 | 2.371 | 2.256 | 1.219 | 1.331 | 1.275 |
| ADT 51 | 1.630 | 1.662 | 1.646 | 1.446 | 1.588 | 1.517 | 1.412 | 1.477 | 1.444 | 1.722 | 1.736 | 1.729 |
| ADT 52 | 2.375 | 2.581 | 2.478 | 1.121 | 1.182 | 1.151 | 1.124 | 1.105 | 1.115 | 2.636 | 2.518 | 2.577 |
| ADT 53 | 1.978 | 2.226 | 2.102 | 1.290 | 1.432 | 1.361 | 1.210 | 1.274 | 1.242 | 1.291 | 1.318 | 1.304 |
| ADT 54 | 1.456 | 1.536 | 1.496 | 0.746 | 0.799 | 0.772 | 0.795 | 0.853 | 0.824 | 1.333 | 1.383 | 1.358 |
| ADT 56 | 1.061 | 1.044 | 1.053 | 0.837 | 0.940 | 0.888 | 0.832 | 0.839 | 0.835 | 2.062 | 2.108 | 2.085 |
| ADT 57 | 1.518 | 1.661 | 1.589 | 0.641 | 0.645 | 0.643 | 0.635 | 0.685 | 0.660 | 1.391 | 1.348 | 1.370 |
| ANNA R 4 | 0.179 | 0.187 | 0.183 | 1.769 | 1.811 | 1.790 | 1.652 | 1.692 | 1.672 | 2.000 | 1.926 | 1.963 |
| APD19002 | 1.144 | 1.106 | 1.125 | 0.905 | 0.949 | 0.927 | 0.915 | 0.947 | 0.931 | 1.415 | 1.378 | 1.396 |
| Arupatham samba | 2.355 | 2.420 | 2.388 | 1.986 | 2.038 | 2.012 | 1.862 | 1.954 | 1.908 | 2.764 | 2.877 | 2.820 |
| CB 16656 | 1.947 | 2.107 | 2.027 | 1.953 | 1.863 | 1.908 | 2.350 | 2.350 | 2.350 | 1.690 | 1.776 | 1.733 |
| CB 17502 | 2.232 | 2.329 | 2.281 | 1.389 | 1.503 | 1.446 | 1.298 | 1.289 | 1.293 | 2.244 | 2.305 | 2.275 |
| CB 17542 | 2.641 | 2.687 | 2.664 | 1.827 | 1.889 | 1.858 | 1.852 | 1.831 | 1.842 | 3.400 | 3.485 | 3.443 |
| CB 17561 | 2.511 | 2.353 | 2.432 | 1.541 | 1.621 | 1.581 | 1.532 | 1.592 | 1.562 | 2.736 | 2.832 | 2.784 |
| CB 17573 | 2.684 | 2.888 | 2.786 | 1.641 | 1.849 | 1.745 | 1.498 | 1.433 | 1.466 | 1.238 | 1.330 | 1.284 |
| CB 17597 | 1.690 | 1.722 | 1.706 | 0.851 | 0.849 | 0.850 | 0.758 | 0.772 | 0.765 | 1.377 | 1.417 | 1.397 |
| CB 22504 | 2.558 | 2.704 | 2.631 | 1.882 | 1.923 | 1.903 | 1.961 | 2.175 | 2.068 | 3.131 | 3.443 | 3.287 |
| CB 22512 | 2.037 | 2.120 | 2.079 | 1.491 | 1.577 | 1.534 | 1.261 | 1.388 | 1.325 | 2.000 | 2.074 | 2.037 |
| CB 22541 | 2.270 | 2.298 | 2.284 | 1.253 | 1.376 | 1.314 | 1.421 | 1.380 | 1.400 | 2.666 | 2.800 | 2.733 |
| CB 22560 | 2.540 | 2.657 | 2.599 | 1.959 | 2.069 | 2.014 | 1.856 | 1.928 | 1.892 | 2.755 | 2.908 | 2.831 |
| ChittanSamba | 1.915 | 2.126 | 2.021 | 2.187 | 2.271 | 2.229 | 2.020 | 1.887 | 1.953 | 2.441 | 2.476 | 2.459 |
| CO 49 | 1.636 | 1.546 | 1.591 | 1.127 | 1.088 | 1.107 | 1.112 | 1.235 | 1.173 | 1.653 | 1.718 | 1.686 |
| CO 51 | 0.822 | 0.849 | 0.835 | 2.300 | 2.231 | 2.266 | 2.100 | 2.024 | 2.062 | 1.642 | 1.675 | 1.658 |
| CO 52 | 0.055 | 0.056 | 0.056 | 1.612 | 1.673 | 1.642 | 1.421 | 1.415 | 1.418 | 3.564 | 3.578 | 3.571 |
| CO 53 | 0.639 | 0.658 | 0.648 | 1.146 | 1.108 | 1.127 | 1.325 | 1.356 | 1.340 | 2.543 | 2.622 | 2.582 |
| CO 54 | 2.354 | 2.405 | 2.379 | 2.307 | 2.361 | 2.334 | 2.203 | 2.281 | 2.242 | 1.511 | 1.605 | 1.558 |
| CO 55 | 1.491 | 1.587 | 1.539 | 1.562 | 1.723 | 1.642 | 1.521 | 1.707 | 1.614 | 1.755 | 1.680 | 1.718 |
| FL 478 | 2.840 | 2.895 | 2.868 | 1.500 | 1.504 | 1.502 | 1.986 | 1.949 | 1.968 | 1.746 | 1.937 | 1.842 |
| FR 13A | 1.892 | 2.047 | 1.970 | 2.433 | 2.562 | 2.498 | 2.312 | 2.608 | 2.460 | 1.941 | 2.071 | 2.006 |
| IR 42 | 2.308 | 2.186 | 2.247 | 0.641 | 0.381 | 0.511 | 1.050 | 1.271 | 1.160 | 3.347 | 3.968 | 3.658 |
| IR 64 | 2.703 | 2.595 | 2.649 | 1.692 | 1.712 | 1.702 | 1.652 | 1.702 | 1.677 | 2.384 | 2.503 | 2.444 |
| IR 64 DRT | 3.000 | 3.155 | 3.077 | 1.171 | 1.217 | 1.194 | 1.980 | 1.978 | 1.979 | 2.612 | 2.643 | 2.628 |
| Kappikar | 1.769 | 1.823 | 1.796 | 2.110 | 2.148 | 2.129 | 2.050 | 2.322 | 2.186 | 1.890 | 1.929 | 1.910 |
| Kattuponni | 1.714 | 1.795 | 1.755 | 1.532 | 1.715 | 1.624 | 1.230 | 1.294 | 1.262 | 2.483 | 2.494 | 2.488 |
| Mattaikar | 2.131 | 2.092 | 2.111 | 0.935 | 0.966 | 0.950 | 0.895 | 0.912 | 0.903 | 3.909 | 3.978 | 3.943 |
| Norungan | 1.225 | 1.338 | 1.281 | 1.687 | 1.754 | 1.720 | 1.986 | 2.026 | 2.006 | 3.573 | 3.658 | 3.616 |
| Ponmani Samba | 1.896 | 1.914 | 1.905 | 1.508 | 1.408 | 1.458 | 1.402 | 1.450 | 1.426 | 2.383 | 2.489 | 2.436 |
| Poongar | 1.921 | 1.925 | 1.923 | 1.597 | 1.686 | 1.641 | 1.423 | 1.507 | 1.465 | 2.275 | 2.354 | 2.314 |
| Upumolagai | 1.788 | 1.947 | 1.867 | 1.300 | 1.453 | 1.377 | 1.410 | 1.379 | 1.394 | 2.740 | 2.825 | 2.782 |
| Vadakathi Samba | 1.800 | 1.802 | 1.801 | 2.358 | 2.370 | 2.364 | 2.124 | 2.401 | 2.262 | 2.520 | 2.433 | 2.477 |
| Varigarudan Samba | 1.772 | 1.871 | 1.821 | 1.930 | 1.821 | 1.875 | 1.962 | 2.001 | 1.981 | 2.600 | 2.699 | 2.649 |
| **Mean** | **1.845** | **1.905** | **1.875** | **1.554** | **1.601** | **1.577** | **1.549** | **1.611** | **1.580** | **2.258** | **2.332** | **2.295** |
| **LSD** | **0.188** | **0.097** | **0.156** | **0.163** | **0.081** | **0.142** | **0.163** | **0.081** | **0.165** | **0.194** | **0.118** | **0.196** |
| **CV** | **6.275** | **3.144** | **4.117** | **6.475** | **3.107** | **4.469** | **6.477** | **3.092** | **5.160** | **5.280** | **3.104** | **4.228** |
| **SE** | **0.066** | **0.035** | **0.055** | **0.058** | **0.029** | **0.050** | **0.058** | **0.029** | **0.058** | **0.069** | **0.042** | **0.069** |
| **Percent Reduction** | **0.000** | **0.000** | **0.000** | **15.793** | **15.930** | **15.893** | **16.016** | **15.444** | **15.733** | **-22.393** | **-22.445** | **-22.400** |
