# Supplementary material for: Enhancing stress resilience in rice (Oryza sativa L.) through profiling early-stage morpho-physiological and molecular responses to multiple abiotic stress tolerance
Source: Front Plant Sci. 2024 Feb 8;15:1342441. doi: 10.3389/fpls.2024.1342441 (PMC10882102; doi:10.3389/fpls.2024.1342441)
Supplement: Supplementary file 6 [file Table_6.docx]

**Supplementary Table 6. Jaccard similarity distance of forty-one rice genotypes using linked marker**

| **Genotypes** | **2** | **3** | **4** | **5** | **6** | **7** | **8** | **9** | **10** | **11** | **12** | **13** | **14** | **15** | **16** | **17** | **18** | **19** | **20** | **21** | **22** |
| --- | --- | --- | --- | --- | --- | --- | --- | --- | --- | --- | --- | --- | --- | --- | --- | --- | --- | --- | --- | --- | --- |
| **1** | 0.353 | 0.473 | 0.500 | 0.491 | 0.519 | 0.509 | 0.472 | 0.434 | 0.415 | 0.412 | 0.536 | 0.434 | 0.373 | 0.526 | 0.455 | 0.469 | 0.518 | 0.567 | 0.567 | 0.500 | 0.463 |
| **2** |  | 0.340 | 0.300 | 0.260 | 0.415 | 0.404 | 0.365 | 0.224 | 0.275 | 0.396 | 0.407 | 0.294 | 0.389 | 0.456 | 0.411 | 0.537 | 0.418 | 0.475 | 0.333 | 0.375 | 0.418 |
| **3** |  |  | 0.455 | 0.294 | 0.385 | 0.404 | 0.396 | 0.327 | 0.400 | 0.365 | 0.436 | 0.418 | 0.327 | 0.370 | 0.352 | 0.589 | 0.500 | 0.548 | 0.448 | 0.458 | 0.418 |
| **4** |  |  |  | 0.353 | 0.412 | 0.431 | 0.453 | 0.213 | 0.300 | 0.453 | 0.491 | 0.353 | 0.444 | 0.534 | 0.464 | 0.510 | 0.526 | 0.446 | 0.500 | 0.483 | 0.415 |
| **5** |  |  |  |  | 0.271 | 0.327 | 0.415 | 0.245 | 0.327 | 0.444 | 0.482 | 0.377 | 0.377 | 0.500 | 0.400 | 0.607 | 0.464 | 0.492 | 0.382 | 0.421 | 0.377 |
| **6** |  |  |  |  |  | 0.354 | 0.500 | 0.404 | 0.320 | 0.412 | 0.453 | 0.434 | 0.340 | 0.526 | 0.396 | 0.611 | 0.463 | 0.542 | 0.436 | 0.446 | 0.434 |
| **7** |  |  |  |  |  |  | 0.462 | 0.360 | 0.404 | 0.462 | 0.579 | 0.327 | 0.392 | 0.544 | 0.473 | 0.604 | 0.536 | 0.534 | 0.509 | 0.542 | 0.453 |
| **8** |  |  |  |  |  |  |  | 0.286 | 0.365 | 0.360 | 0.491 | 0.415 | 0.415 | 0.396 | 0.346 | 0.480 | 0.353 | 0.474 | 0.389 | 0.370 | 0.415 |
| **9** |  |  |  |  |  |  |  |  | 0.260 | 0.385 | 0.482 | 0.245 | 0.346 | 0.418 | 0.308 | 0.471 | 0.436 | 0.439 | 0.411 | 0.421 | 0.346 |
| **10** |  |  |  |  |  |  |  |  |  | 0.265 | 0.377 | 0.260 | 0.358 | 0.456 | 0.382 | 0.509 | 0.418 | 0.448 | 0.364 | 0.345 | 0.327 |
| **11** |  |  |  |  |  |  |  |  |  |  | 0.463 | 0.385 | 0.353 | 0.509 | 0.407 | 0.417 | 0.500 | 0.525 | 0.446 | 0.429 | 0.385 |
| **12** |  |  |  |  |  |  |  |  |  |  |  | 0.426 | 0.396 | 0.377 | 0.446 | 0.519 | 0.365 | 0.429 | 0.370 | 0.352 | 0.396 |
| **13** |  |  |  |  |  |  |  |  |  |  |  |  | 0.314 | 0.500 | 0.429 | 0.528 | 0.491 | 0.466 | 0.466 | 0.500 | 0.407 |
| **14** |  |  |  |  |  |  |  |  |  |  |  |  |  | 0.358 | 0.340 | 0.528 | 0.436 | 0.439 | 0.411 | 0.448 | 0.314 |
| **15** |  |  |  |  |  |  |  |  |  |  |  |  |  |  | 0.321 | 0.509 | 0.294 | 0.333 | 0.333 | 0.375 | 0.389 |
| **16** |  |  |  |  |  |  |  |  |  |  |  |  |  |  |  | 0.400 | 0.240 | 0.375 | 0.345 | 0.264 | 0.370 |
| **17** |  |  |  |  |  |  |  |  |  |  |  |  |  |  |  |  | 0.440 | 0.472 | 0.500 | 0.481 | 0.408 |
| **18** |  |  |  |  |  |  |  |  |  |  |  |  |  |  |  |  |  | 0.411 | 0.255 | 0.235 | 0.436 |
| **19** |  |  |  |  |  |  |  |  |  |  |  |  |  |  |  |  |  |  | 0.386 | 0.424 | 0.321 |
| **20** |  |  |  |  |  |  |  |  |  |  |  |  |  |  |  |  |  |  |  | 0.245 | 0.288 |
| **21** |  |  |  |  |  |  |  |  |  |  |  |  |  |  |  |  |  |  |  |  | 0.393 |

**Jaccard similarity distance of forty-one rice genotypes continue…**

| **Genotypes** | **23** | **24** | **25** | **26** | **27** | **28** | **29** | **30** | **31** | **32** | **33** | **34** | **35** | **36** | **37** | **38** | **39** | **40** | **41** |
| --- | --- | --- | --- | --- | --- | --- | --- | --- | --- | --- | --- | --- | --- | --- | --- | --- | --- | --- | --- |
| **1** | 0.412 | 0.518 | 0.519 | 0.491 | 0.471 | 0.482 | 0.500 | 0.509 | 0.462 | 0.380 | 0.471 | 0.550 | 0.415 | 0.500 | 0.526 | 0.589 | 0.554 | 0.593 | 0.456 |
| **2** | 0.265 | 0.500 | 0.473 | 0.500 | 0.561 | 0.492 | 0.400 | 0.544 | 0.385 | 0.482 | 0.509 | 0.431 | 0.400 | 0.392 | 0.370 | 0.373 | 0.509 | 0.418 | 0.386 |
| **3** | 0.482 | 0.574 | 0.526 | 0.500 | 0.423 | 0.466 | 0.483 | 0.544 | 0.473 | 0.482 | 0.536 | 0.508 | 0.370 | 0.453 | 0.370 | 0.491 | 0.559 | 0.500 | 0.386 |
| **4** | 0.423 | 0.500 | 0.277 | 0.444 | 0.564 | 0.377 | 0.509 | 0.491 | 0.472 | 0.481 | 0.564 | 0.508 | 0.482 | 0.481 | 0.455 | 0.400 | 0.509 | 0.576 | 0.411 |
| **5** | 0.385 | 0.517 | 0.518 | 0.517 | 0.500 | 0.456 | 0.474 | 0.586 | 0.434 | 0.526 | 0.527 | 0.393 | 0.358 | 0.472 | 0.389 | 0.481 | 0.500 | 0.517 | 0.375 |
| **6** | 0.472 | 0.491 | 0.571 | 0.463 | 0.375 | 0.509 | 0.552 | 0.589 | 0.431 | 0.579 | 0.500 | 0.500 | 0.385 | 0.471 | 0.415 | 0.537 | 0.527 | 0.569 | 0.400 |
| **7** | 0.491 | 0.481 | 0.481 | 0.481 | 0.396 | 0.500 | 0.569 | 0.556 | 0.481 | 0.545 | 0.547 | 0.436 | 0.373 | 0.547 | 0.569 | 0.500 | 0.491 | 0.586 | 0.446 |
| **8** | 0.423 | 0.552 | 0.554 | 0.500 | 0.537 | 0.436 | 0.300 | 0.491 | 0.527 | 0.509 | 0.481 | 0.429 | 0.426 | 0.388 | 0.426 | 0.400 | 0.453 | 0.473 | 0.352 |
| **9** | 0.353 | 0.464 | 0.373 | 0.491 | 0.500 | 0.340 | 0.418 | 0.453 | 0.434 | 0.473 | 0.472 | 0.421 | 0.358 | 0.442 | 0.389 | 0.392 | 0.500 | 0.517 | 0.283 |
| **10** | 0.265 | 0.418 | 0.500 | 0.474 | 0.509 | 0.382 | 0.400 | 0.434 | 0.250 | 0.482 | 0.453 | 0.345 | 0.370 | 0.392 | 0.370 | 0.434 | 0.534 | 0.500 | 0.296 |
| **11** | 0.392 | 0.473 | 0.579 | 0.576 | 0.481 | 0.491 | 0.509 | 0.491 | 0.442 | 0.481 | 0.509 | 0.508 | 0.396 | 0.420 | 0.455 | 0.519 | 0.509 | 0.552 | 0.382 |
| **12** | 0.340 | 0.365 | 0.481 | 0.426 | 0.545 | 0.474 | 0.377 | 0.527 | 0.392 | 0.463 | 0.261 | 0.439 | 0.377 | 0.333 | 0.314 | 0.380 | 0.544 | 0.300 | 0.421 |
| **13** | 0.353 | 0.346 | 0.434 | 0.517 | 0.554 | 0.429 | 0.525 | 0.561 | 0.434 | 0.500 | 0.442 | 0.364 | 0.389 | 0.442 | 0.474 | 0.423 | 0.526 | 0.464 | 0.315 |
| **14** | 0.415 | 0.436 | 0.569 | 0.567 | 0.412 | 0.483 | 0.525 | 0.561 | 0.518 | 0.353 | 0.412 | 0.448 | 0.149 | 0.412 | 0.418 | 0.561 | 0.473 | 0.542 | 0.250 |
| **15** | 0.482 | 0.525 | 0.526 | 0.525 | 0.481 | 0.466 | 0.340 | 0.491 | 0.500 | 0.365 | 0.481 | 0.404 | 0.308 | 0.423 | 0.275 | 0.373 | 0.482 | 0.418 | 0.386 |
| **16** | 0.436 | 0.483 | 0.534 | 0.483 | 0.373 | 0.421 | 0.439 | 0.444 | 0.455 | 0.407 | 0.404 | 0.357 | 0.321 | 0.306 | 0.220 | 0.444 | 0.436 | 0.456 | 0.368 |
| **17** | 0.480 | 0.582 | 0.558 | 0.556 | 0.569 | 0.545 | 0.537 | 0.549 | 0.558 | 0.449 | 0.479 | 0.561 | 0.509 | 0.413 | 0.537 | 0.520 | 0.510 | 0.582 | 0.544 |
| **18** | 0.444 | 0.491 | 0.593 | 0.464 | 0.472 | 0.533 | 0.294 | 0.481 | 0.491 | 0.415 | 0.412 | 0.333 | 0.389 | 0.313 | 0.260 | 0.360 | 0.526 | 0.346 | 0.458 |
| **19** | 0.446 | 0.382 | 0.464 | 0.587 | 0.600 | 0.458 | 0.475 | 0.559 | 0.542 | 0.389 | 0.473 | 0.368 | 0.393 | 0.415 | 0.393 | 0.426 | 0.188 | 0.517 | 0.433 |
| **20** | 0.358 | 0.492 | 0.590 | 0.565 | 0.526 | 0.508 | 0.333 | 0.534 | 0.436 | 0.500 | 0.444 | 0.278 | 0.333 | 0.415 | 0.302 | 0.426 | 0.500 | 0.321 | 0.379 |
| **21** | 0.400 | 0.448 | 0.574 | 0.475 | 0.455 | 0.467 | 0.345 | 0.436 | 0.389 | 0.483 | 0.365 | 0.321 | 0.375 | 0.396 | 0.283 | 0.377 | 0.508 | 0.333 | 0.273 |
| **22** | 0.353 | 0.407 | 0.491 | 0.567 | 0.472 | 0.400 | 0.446 | 0.509 | 0.434 | 0.353 | 0.380 | 0.364 | 0.224 | 0.412 | 0.418 | 0.509 | 0.444 | 0.517 | 0.345 |
| **23** |  | 0.415 | 0.472 | 0.500 | 0.614 | 0.464 | 0.396 | 0.519 | 0.313 | 0.453 | 0.420 | 0.340 | 0.426 | 0.319 | 0.396 | 0.431 | 0.509 | 0.444 | 0.439 |
| **24** |  |  | 0.434 | 0.464 | 0.500 | 0.456 | 0.525 | 0.561 | 0.518 | 0.552 | 0.412 | 0.448 | 0.418 | 0.527 | 0.525 | 0.481 | 0.500 | 0.491 | 0.431 |
| **25** |  |  |  | 0.340 | 0.528 | 0.265 | 0.444 | 0.388 | 0.462 | 0.500 | 0.528 | 0.550 | 0.552 | 0.556 | 0.500 | 0.388 | 0.554 | 0.544 | 0.533 |
| **26** |  |  |  |  | 0.412 | 0.400 | 0.389 | 0.423 | 0.404 | 0.552 | 0.472 | 0.548 | 0.525 | 0.500 | 0.525 | 0.423 | 0.623 | 0.464 | 0.532 |
| **27** |  |  |  |  |  | 0.434 | 0.509 | 0.396 | 0.471 | 0.509 | 0.510 | 0.559 | 0.327 | 0.566 | 0.481 | 0.547 | 0.614 | 0.579 | 0.491 |
| **28** |  |  |  |  |  |  | 0.352 | 0.353 | 0.333 | 0.464 | 0.463 | 0.492 | 0.411 | 0.544 | 0.439 | 0.500 | 0.517 | 0.557 | 0.368 |
| **29** |  |  |  |  |  |  |  | 0.340 | 0.385 | 0.509 | 0.481 | 0.404 | 0.456 | 0.423 | 0.340 | 0.340 | 0.583 | 0.418 | 0.467 |
| **30** |  |  |  |  |  |  |  |  | 0.388 | 0.462 | 0.490 | 0.542 | 0.491 | 0.547 | 0.434 | 0.500 | 0.644 | 0.586 | 0.525 |
| **31** |  |  |  |  |  |  |  |  |  | 0.472 | 0.375 | 0.418 | 0.415 | 0.471 | 0.415 | 0.481 | 0.579 | 0.463 | 0.429 |
| **32** |  |  |  |  |  |  |  |  |  |  | 0.388 | 0.483 | 0.300 | 0.420 | 0.426 | 0.545 | 0.481 | 0.552 | 0.492 |
| **33** |  |  |  |  |  |  |  |  |  |  |  | 0.426 | 0.327 | 0.417 | 0.481 | 0.547 | 0.509 | 0.412 | 0.377 |
| **34** |  |  |  |  |  |  |  |  |  |  |  |  | 0.375 | 0.396 | 0.345 | 0.377 | 0.456 | 0.393 | 0.362 |
| **35** |  |  |  |  |  |  |  |  |  |  |  |  |  | 0.423 | 0.400 | 0.544 | 0.455 | 0.525 | 0.264 |
| **36** |  |  |  |  |  |  |  |  |  |  |  |  |  |  | 0.292 | 0.396 | 0.451 | 0.472 | 0.464 |
| **37** |  |  |  |  |  |  |  |  |  |  |  |  |  |  |  | 0.306 | 0.509 | 0.358 | 0.441 |
| **38** |  |  |  |  |  |  |  |  |  |  |  |  |  |  |  |  | 0.545 | 0.292 | 0.500 |
| **39** |  |  |  |  |  |  |  |  |  |  |  |  |  |  |  |  |  | 0.600 | 0.466 |
| **40** |  |  |  |  |  |  |  |  |  |  |  |  |  |  |  |  |  |  | 0.483 |
|  |  |  |  |  |  |  |  |  |  |  |  |  |  |  |  |  |  | Average | 0.443 |

*1 to 41 indicates the genotypes listed in Table 1
